# A systematic review of the natural history and biomarkers of primary lecithin:cholesterol acyltransferase deficiency

**DOI:** 10.1016/j.jlr.2022.100169

**Published:** 2022-01-20

**Authors:** Cecilia Vitali, Archna Bajaj, Christina Nguyen, Jill Schnall, Jinbo Chen, Kostas Stylianou, Daniel J. Rader, Marina Cuchel

**Affiliations:** 1 Division of Translational Medicine and Human Genetics, Department of Medicine, Perelman School of Medicine, University of Pennsylvania, Philadelphia, PA, USA; 2 Department of Biostatistics, Epidemiology and Informatics, University of Pennsylvania, Philadelphia, PA, USA; 3 Department of Nephrology, Heraklion University Hospital, Crete, Greece; 4 Department of Genetics, Perelman School of Medicine, University of Pennsylvania, Philadelphia, PA, USA

**Keywords:** lecithin:cholesterol acyltransferase, familial LCAT deficiency, fish-eye disease, nephrotic syndrome, systematic review, chronic kidney disease, proteinuria/hematuria, HDL, esterified cholesterol, human data, apoB, apolipoprotein B, ASCVD, atherosclerotic cardiovascular disease, CKD, chronic kidney disease, EC, esterified cholesterol, eGFR, estimated glomerular filtration rate, ESRD, end-stage renal disease, FED, fish-eye disease, FLD, familial LCAT deficiency, IQR, interquartile range, LpX, lipoprotein X, TC, total cholesterol, TG, triglyceride, UC, unesterified cholesterol

## Abstract

Syndromes associated with LCAT deficiency, a rare autosomal recessive condition, include fish-eye disease (FED) and familial LCAT deficiency (FLD). FLD is more severe and characterized by early and progressive chronic kidney disease (CKD). No treatment is currently available for FLD, but novel therapeutics are under development. Furthermore, although biomarkers of LCAT deficiency have been identified, their suitability to monitor disease progression and therapeutic efficacy is unclear, as little data exist on the rate of progression of renal disease. Here, we systematically review observational studies of FLD, FED, and heterozygous subjects, which summarize available evidence on the natural history and biomarkers of LCAT deficiency, in order to guide the development of novel therapeutics. We identified 146 FLD and 53 FED patients from 219 publications, showing that both syndromes are characterized by early corneal opacity and markedly reduced HDL-C levels. Proteinuria/hematuria were the first signs of renal impairment in FLD, followed by rapid decline of renal function. Furthermore, LCAT activity toward endogenous substrates and the percentage of circulating esterified cholesterol (EC%) were the best discriminators between these two syndromes. In FLD, higher levels of total, non-HDL, and unesterified cholesterol were associated with severe CKD. We reveal a nonlinear association between LCAT activity and EC% levels, in which subnormal levels of LCAT activity were associated with normal EC%. This review provides the first step toward the identification of disease biomarkers to be used in clinical trials and suggests that restoring LCAT activity to subnormal levels may be sufficient to prevent renal disease progression.

Familial LCAT deficiency (FLD) is an ultrarare autosomal recessive disorder that causes progressive chronic kidney disease (CKD) and early end-stage renal disease (ESRD), usually by the fourth decade of life (1). It is caused by pathogenic variants in the gene encoding for LCAT, an enzyme mainly secreted by the liver that is solely responsible for the esterification of cholesterol in plasma lipoproteins (2). Its preferential substrate is HDL (2), and LCAT activity targeted toward HDL (alpha activity) is especially important for the normal maturation and metabolism of HDL; however, it can also esterify cholesterol in apolipoprotein B (apoB)-containing lipoprotein (beta activity) (2).

Biallelic mutations in the *LCAT* gene that reduce LCAT secretion or function result in LCAT deficiency, which manifests as two related but different syndromes: FLD and fish-eye disease (FED) (Orphanet: an online database of rare diseases and orphan drugs; http://www.orpha.net; accession numbers: ORPHA: 79293 and ORPHA: 79292). Both are characterized by very low levels of HDL-C and corneal opacities because of deposition of unesterified cholesterol (UC) in the cornea. Notably, FLD patients, but not FED patients, develop early and progressive CKD leading to ESRD, which is the main cause of morbidity and mortality in this population (1). Heterozygous subjects display a semidominant lipid phenotype and no ocular or renal manifestations. Risk of developing atherosclerotic cardiovascular disease (ASCVD) is variable and still debated (3, 4, 5, 6).

The difference in phenotype between FLD and FED has been attributed to the extent of residual LCAT activity, with variants associated with complete (alpha and beta) loss of activity causing FLD and variants associated with partial loss of alpha and beta activity, or complete loss of only alpha activity, but with preserved beta activity causing FED (7).

Although the molecular bases of FLD are well understood (7), the etiology of renal damage is still unclear. An abnormal lipoprotein (lipoprotein X [LpX]) has been frequently observed in FLD, and some evidence suggests that this particle enriched in UC is directly nephrotoxic (1, 8). However, little data are available to exclude its presence in FED. Clinical presentation of the renal disease is that of nephrotic syndrome, and histological characteristics include segmental glomerular sclerosis, together with mesangial deposits and thickening of the glomerular basement membrane (1).

Current treatment for FLD is limited to preserving residual renal function, with no treatment specifically targeting the underlying disease process. However, this approach is not sufficient, and FLD patients often progress to ESRD requiring renal replacement therapy, namely dialysis and kidney transplantation, by the third or fourth decade of life. In recent years, several potential therapeutic approaches have been investigated and provided promising results. These include enzyme replacement therapy, liver-directed *LCAT* gene therapy, engineered-cell therapy, and LCAT activators (9).

Pivotal to the further development of such treatments is the knowledge of the natural history of the disease and the evaluation of diagnostic and prognostic biomarkers to be used in clinical trials (10). In the context of FLD, more information on the progression of renal disease and the identification of biomarkers that can be used to assess treatment efficacy beyond an effect on lipid levels are particularly needed.

Here, we present a comprehensive, up-to-date, systematic review of the published literature on LCAT deficiency disorders. The primary motivation for this review was to summarize evidence on proposed biomarkers that can be used to diagnose FLD or FED, assess renal disease progression, and identify possible disease modulators. The results of this study provide an important step in gathering the necessary information for the further development of novel therapeutics.

## Materials and methods

### Search strategy

We searched the PubMed database from the date of the first case description (1967) to April 22, 2020 for records in English language, describing cases of LCAT deficiency and the biochemical characterization of causative variants. The search was conducted following the guidelines of the Preferred Reporting Items for Systematic Reviews and Meta-Analyses statement (11). Records were first screened based on titles and abstracts. At this level, we excluded records unrelated to LCAT deficiency, not in English, animal-only studies, studies on secondary LCAT deficiency, books, abstracts, webpages, dissertations, articles in press, and personal communications. Records that passed the first screening were retrieved in full text. At this stage, in addition to the criteria listed previously, we also excluded records that reported outcomes not captured by this study (noneligible outcomes), presented in a nonextractable form, or records for which we were unable to retrieve the full text. In order to maximize our ability to capture potentially relevant studies, we also screened all the records cited in the reference section of articles included in the analysis. Additional information, including the electronic search strategy and outcome definitions, are provided in the supplemental methods section.

### Assessment of quality and risk of bias

Given the rarity of LCAT deficiency, there are few published reports; most of them are case reports and family studies. The selected studies showed a high degree of heterogeneity in the data described. However, all studies that met our screening criteria were included in the study, to maximize the limited data available. A formal quality assessment of the studies was not possible.

### Data synthesis and analysis

Patients with a clinical diagnosis of FLD and FED, and heterozygous carriers of variants identified in homozygous FLD and FED patients (FLD-Het and FED-Het, respectively), were analyzed using a quantitative method. Heterozygous carriers of variants that could not be conclusively identified as FLD or FED causing were not included in the analysis. Subjects who presented with atypical clinical and genetic features are presented separately in supplemental Table S4 and were not included in the quantitative analysis. Subjects with no FLD or FED clinical phenotype and unknown genotype were excluded from analysis. The detailed criteria for the classification of subjects into categories are presented in the supplemental methods section. Data on the biochemical characteristics of variants identified in FLD and FED patients were summarized descriptively.

### Statistics

For continuous variables, medians and interquartile ranges (IQRs) were calculated, and differences between groups were assessed using Wilcoxon rank sum test. For categorical variables, data were expressed as absolute numbers of observations/category and as percentage of total entries. Statistically significant differences between the expected frequencies and the observed frequencies were assessed with Chi-squared (sample size >5) or Fisher's exact tests (sample size <5), as appropriate.

Change of annual rate of estimated glomerular filtration rate (eGFR) refers to the slope that was obtained for patients with two or more eGFR values, by linear regression analysis of eGFR and age.

Effect of biological covariables on clinical outcomes was assessed using multivariate linear regression (continuous dependent variables) or logistic regression (categorical dependent variables) models. Additional information on the regression analyses performed are reported in the supplemental methods section. Analyses were performed using SAS 9.4 (SAS Institute, Inc). An alpha of 0.05 determined statistical significance.

## Results

### Search and study selection

**Fig. 1:**
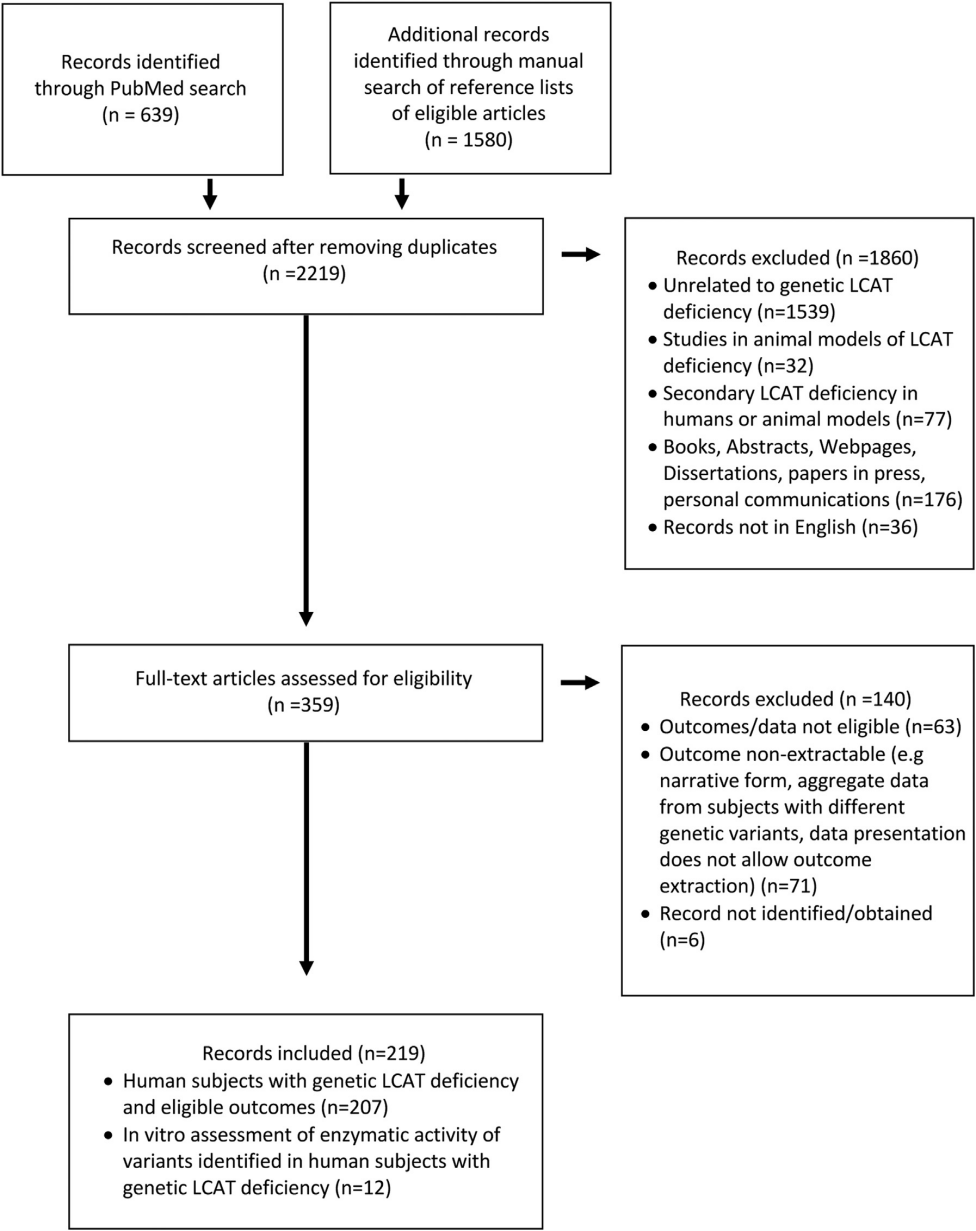
Flowchart of record screening and selection. The flowchart has been compiled according to the guidelines of the PRISMA statement (11). A list of eligible publications and their characteristics is presented in supplemental Table S1.

We searched the PubMed database for records related to LCAT deficiency. We identified 639 unique records; 1,580 additional records were identified through a manual search of reference lists of all included articles. Of the 219 articles that met the inclusion criteria for our review, 207 studies reported information on human patients with primary LCAT deficiency and 12 studies reported information on the biochemical properties of FED-causing and FLD-causing variants (Fig. 1 and supplemental Table S1).

### Classification of studied subjects

The article review returned a total of 564 subjects. Because one of the main goals of this systematic review was the evaluation of diagnostic and prognostic biomarkers, we based our primary classification solely on clinical and genetic characteristics and retrospectively analyzed the relationship between biochemical measurements and clinical classification.

Subjects were classified as FED patients if they had corneal opacity and HDL-C levels below the reference threshold or, in the absence of HDL-C levels, alpha-LCAT enzymatic activity <60% of the value in healthy controls described in the same report. Subjects were classified as FLD patients if, in addition to the criteria for FED, they also had renal abnormalities, were homozygous carriers of a variant previously reported to be FLD causing, or were identified within the same family of an FLD patient. A diagram summarizing the classification of subjects into categories is presented in the supplemental methods section. About 146 patients met the criteria for FLD, and 53 patients met the criteria for FED classification (Table 1). The clinical diagnosis was complemented by genetic testing in 153 of the 199 FLD and FED cases. Collectively, FLD and FED patients carried 89 different variants (supplemental Tables S2 and S3).

**Table 1:** Demographic characteristics of FED and FLD patients

Characteristic	Patient's Clinical Phenotype		*P*^a^
	FLD	FED	
Total number of subjects, n (%)^b^	146	53	
Sex, n (%)^b^			0.0447
Male	92 (63.0)	25 (47.2)	
Region of provenience, n (%)^b^			0.0021
Asia	22 (15.1)	6 (11.3)	
Australia	2 (1.4)	1 (1.9)	
Canada/USA	22 (15.1)	6 (11.3)	
Europe	79 (54.1)	31 (58.5)	
Latin America	0 (0.0)	3 (5.7)	
Middle East	12 (8.2)	2 (3.8)	
North Africa	3 (2.1)	3 (5.7)	
South Asia	6 (4.1)	1 (1.9)	
Genetic confirmation of clinical diagnosis, n (%)^b^	106 (72.6)	47 (88.7)	0.0174

a Chi-squared test (sex, genetic confirmation of clinical diagnosis, and sample size >5) or Fisher's exact tests (ethnicity, sample size <5).

b Categorical variables are expressed as count (percent of total count).

Heterozygous subjects were classified based on the variant carried: 195 subjects carried variants previously identified in homozygous FLD patients (FLD-Het) and 100 subjects carried variants previously identified in homozygous FED patients (FED-Het) (Table 2 and supplemental Table S2). Additional 54 heterozygous subjects had variants that could not be definitively classified as either FLD or FED causing and were not included in the analysis (supplemental Table S3). Finally, 16 subjects could not be classified because of unusual clinical and genetic features and are described separately in the supplemental data (supplemental Table S4).

**Table 2:** Demographic characteristics of heterozygous subjects

Characteristic	Predicted Effect of the Variant on Clinical Phenotype		*P*^a^
	FLD-Causing Variant	FED-Causing Variant	
Total number of subjects, n (%)	195	100	
Sex, n (%)^b^			0.0562
Male	92 (47.2)	61 (61.0)	
Female	95 (48.7)	39 (39.0)	
Not reported	8 (4.1)	0 (0)	
Region of provenience, n (%)^b^			<0.0001
Asia	22 (11.3)	3 (3.0)	
Australia	0 (0.0)	3 (3.0)	
Canada/USA	19 (9.7)	8 (8.0)	
Europe	144 (73.9)	79 (79.0)	
Latin America	0 (0.0)	3 (3.0)	
Middle East	0 (0.0)	0 (0.0)	
North Africa	3 (1.5)	4 (4.0)	
South Asia	7 (3.6)	0 (0.0)	
Genetic confirmation of clinical diagnosis, n (%)^b^	195 (100.0)	100 (100.0)	—

a Chi-squared test (sex, sample size >5, not including the frequency of nonreported) or Fisher’s exact tests (ethnicity, sample size <5).

b Categorical variables are expressed as count (percent of total count).

### Demographic and clinical characteristics

The majority of reported FLD and FED cases were from European countries, and FLD cases were predominantly male (Table 1 and supplemental Fig. S1). The geographical distribution of heterozygous subjects mirrors that found in FED and FED patients (Table 2). Interestingly, the gender distribution of heterozygous subjects showed a comparable proportion of males and females in heterozygous subjects of FED-Het and FLD-Het.

Corneal opacity is often the first clinically recognized sign of LCAT deficiency and was present in all FLD and FED patients at the time of report (Table 3). HDL-C levels were uniformly low in both FLD and FED patients, with most patients having levels <10 mg/dl (third quartile = 13 and 9.4 mg/dl, respectively; Table 4). FLD patients had a significantly higher prevalence of anemia compared with FED patients (Table 3).

**Table 3:** Clinical characteristics of FED and FLD patients

Clinical Trait	Patient's Clinical Phenotype		FED Versus FLD *P*^a^
	FLD	FED	
**Clinical trait (prevalence)**			
Corneal opacity, n/N (%)^b^	146/146 (100)	53/53 (100)	—
Anemia, n/N (%)^b^	109/119 (91.6)	8/33 (24.2)	<0.0001
Proteinuria, n/N (%)^b^	119/136 (87.5)	0/49 (0)	—
Hematuria, n/N (%)^b^	44/46 (95.7)	0/2 (0)	—
CKD stage III–V, n/N (%)^b^	55/103 (53.4)	0/16 (0)	—
CVD, all types, n/N (%)^b^	16/74 (21.6)	14/45 (31.1)	0.2477
Clinically significant ASCVD, n/N (%)^b^	7/68 (10.3)	12/45 (26.7)	0.0227
Asymptomatic ASCVD, n/N (%)^b^	5/30 (16.7)	1/12 (8.3)	0.3260
CVD, other nature, n/N (%)^b^	9/20 (45.0)	2/4 (50.0)	0.4037
**Age at diagnosis/first report of clinical trait**			
Corneal opacity (years)			0.0869
Median	29.5	33.0	
IQR	16.5–37.0	18.0–54	
N	144	50	
Anemia (years)			0.1289
Median	33.0	44.5	
IQR	25.0–44.0	34.0–52.5	
N	107	8	
Proteinuria (years)		—	NA
Median	30.5		
IQR	24.0–38.0		
N	118		
Hematuria (years)		—	NA
Median	31.5		
IQR	24.0–36.0		
N	40		
CKD stage III–V (years)		—	NA
Median	38.0		
IQR	30.0–45.0		
N	54		
CVD, all types (years)			0.0879
Median	44.0	56.0	
IQR	34.0–56.5	43.0–61.0	
N	16	14	
Clinically significant ASCVD (years)			0.8687
Median	55.0	56.0	
IQR	48.0–61.0	43.5–61.5	
N	10	12	
Asymptomatic ASCVD (years)			0.2278
Median	40.0	62.0	
IQR	40.0–53.0	NA	
N	5	1	
CVD, other nature (years)			0.0442
Median	33.5	52.0	
IQR	27.0–36.0	43.0–61.0	
N	6	2	

NA, not available.

a Comparison between frequencies: Chi-squared test (sample size >5) or Fisher's exact tests (sample size <5). Comparison between ages: Wilcoxon rank sum test.

b Categorical variables are expressed as count/total count of available entries, percent of total count (%).

**Table 4:** Lipid, lipoprotein, and LCAT activity levels in FLD and FED patients and heterozygous subjects

Biomarker	Subject Group				FLD Versus FED^a^	FLD-Het Versus FED-Het^a^	FLD Versus FLD-Het^a^	FED Versus FED-Het^a^	Desirable Values in the General Population
	FLD	FED	FLD-Het	FED-Het	*P*				
Lipid and lipoprotein measurements									
TC (mg/dl)					0.0892	0.5633	<0.0001	0.3050	<200
Median	146.0	176.3	175.9	195.1					
IQR	103.9–188.2	113.0–211.9	147.0–206.0	149.0–197.2					
n	126	47	169	94					
HDL–C (mg/dl)					0.0183	0.0094	<0.0001	<0.0001	>40 (men) >50 (women)
Median	7.9	6.2	35.3	31.3					
IQR	5.0–13.0	4.0–9.4	29.4–40.8	24.7–40.0					
n	106	48	156	100					
Non-HDL-C (mg/dl)					0.1413	0.2244	0.5091	0.0894	<160
Median	145.7	170.6	142.0	160.9					
IQR	108.1–185.0	109.4–207.0	107.1–171.2	121.0–165.9					
n	103	46	152	94					
LDL-C (mg/dl)					0.0006	<0.0001	0.0001	0.4546	<129
Median	71.9	113.6	107.1	135.3					
IQR	41.1–110.6	82.0–155.0	83.0–126.1	99.0–139.2					
n	64	40	109	93					
TGs (mg/dl)					0.5437	0.9449	<0.0001	<0.0001	<150
Median	226.5	207.0	120.8	124.0					
IQR	148.3–408.1	146.3–366.4	92.0–212.0	97.7–141.7					
n	116	48	166	100					
ApoA-I (mg/dl)					0.0002	0.1706	<0.0001	<0.0001	>120
Median	43.0	32.0	111.0	104.0					
IQR	37.0–50.8	26.0–43.5	94.0–130.8	99.0–119.0					
n	83	37	105	75					
ApoA-II (mg/dl)					0.0684	0.6213	<0.0001	0.0002	>25
Median	5.1	8.0	29.5	31.9					
IQR	3.5–9.0	6.0–9.0	27.0–34.0	28.0–36.0					
n	43	17	36	7					
ApoB (mg/dl)					<0.0001	0.0741	<0.0001	0.0627	<90
Median	42.8	134.5	100.0	114.0					
IQR	34.3–63.7	91.0–174.0	81.0–127.0	109.0–116.0					
n	66	26	79	57					
LpX (prevalence) n/N (%)^b^	32/36 (88.9)	0/0 (0)	0/1 (0.0)	0 (0)	NA	NA	NA	NA	Absent
LCAT protein, activity measurements, and markers of cholesterol esterification									
Plasma LCAT concentration (% of control values)					<0.0001	<0.0001	<0.0001	<0.0001	100%
Median	10.2	40.4	56.9	74.5					
IQR	1.0–27.9	32.9–49.2	52.6–66.0	72.0–95.8					
n	59	22	63	63					
LCAT activity toward endogenous lipoproteins (% of control values)					<0.0001	0.5390	<0.0001	<0.0001	100%
Median	0.0	53.6	85.8	97.7					
IQR	0.0–4.9	41.5–69.6	59.2–104.1	79.7–102.7					
n	82	33	52	74					
LCAT activity toward exogenous substrate (% of control values)					<0.0001	<0.0001	<0.0001	<0.0001	100%
Median	1.4	5.8	61.3	79.0					
IQR	0.0–4.8	2.1–9.5	50.6–64.7	61.5–83.1					
n	74	40	76	75					
UC (mg/dl)					<0.0001	0.0762	<0.0001	<0.0001	<60
Median	118.5	74.0	48.0	54.1					
IQR	87.0–161.7	61.2–88.9	40.0–59.7	47.6–58.0					
n	102	35	90	65					
EC (% of TC)					<0.0001	0.2277	<0.0001	<0.0001	60–80
Median	11.0	54.0	70.3	72.5					
IQR	7.2–16.2	43.0–61.1	67.6–74.0	71.2–72.5					
n	107	33	90	62					

NA, not available.

a Wilcoxon rank sum test.

b Categorical variables are expressed as count/total count of available entries, percent of total count (%).

### Renal disease

For this systematic review, cases were classified as FLD if they presented with corneal opacity, low HDL-C, and renal disease or if they presented with corneal opacity, low HDL-C, were homozygous carriers of variants previously reported to be FLD causing, or were identified within the same family of an FLD patient. Accordingly, none of the FED patients had renal disease, and 15 FLD patients were still asymptomatic for signs of renal disease at the time of the case report. Proteinuria and hematuria were the first signs of renal impairment, and 55 (53.4%) of the patients eventually developed CKD stage III–V, 34 of whom reached ESRD (median age = 39.5 years; IQR: 33–48) by the time their case was published (Table 3). Twenty patients underwent kidney transplantation (supplemental Table S5). Subsequent kidney failure or rejection has been reported on four of them. One patient received a second transplanted kidney, when the first one failed. Ten of 12 transplanted kidneys for which histology was reported displayed signs consistent with the recurrence of the renal damage, and one displayed unclear results.

Renal biopsies from 57 patients shared several histopathological features showing glomerular changes. Together with segmental glomerular sclerosis, notable findings included expansion and vacuolization of the mesangial area with electron microscopy revealing high-density lipid deposits, depositions of foamy material in the glomerular basement membrane leading to irregular thickening, and capillary loops distended with foamy material. Podocyte foot processes were described as fused or effaced. Immunofluorescence results were mixed. Among the biopsies that provided immunofluorescence data, 62.5% stained positive for complement protein C3 and 53.3% for immunoglobulin M (Table 5 and supplemental Table S6).

**Table 5:** Kidney histologic findings in FLD patients

Histologic Finding	Prevalence (n/N, %)^a^
Glomeruli	
Global and/or segmental glomerular sclerosis	18/20 (90.0)
Vacuolization of glomerular Cells	6/8 (75.0)
Foam cell infiltration	20/25 (80.0)
Macrophage infiltration	3/7 (42.9)
Mesangial matrix expansion	26/29 (89.7)
Mesangial deposits	26/26 (100.0)
Vacuolization of mesangial area	13/13 (100.0)
Mesangial foam cell infiltration	6/8 (75.0)
Thickening of the GBM	24/24 (100.0)
Duplication of the GBM	6/9 (66.7)
Vacuolization of GBM	26/29 (89.7)
Lipid deposits in GBM	14/15 (93.3)
Thickened capillary walls	12/13 (92.3)
Pericapillary depositions	9/9 (100.0)
Effacement of podocyte foot processes	9/9 (100.0)
Tubules/interstitial space	
Tubulointerstitial changes	4/7 (57.1)
Tubular atrophy	13/15 (86.7)
Interstitial fibrosis	10/13 (76.9)
Tubular cell vacuolization	3/6 (50.0)
Thickening of TBM	3/3 (100.0)
TBM vacuolization	3/7 (42.9)
Interstitial inflammation	5/7 (71.4)
Interstitial edema	1/7 (14.3)
Interstitial macrophages	6/6 (100.0)
Vessels	
Intimal thickening	4/6 (66.7)
Hyalinosis	9/10 (90.0)
Pseudothrombi	6/9 (66.7)
Subendothelial deposits	7/7 (100.0)
Immunofluorescence/immunohistochemistry	
IgA	0/14 (0.0)
IgG	1/15 (6.7)
IgM	8/15 (53.3)
C3	10/16 (62.5)
C4	3/12 (25.0)
C1q	5/11 (45.5)

C1q, complement protein C1q; C3, complement protein C3; C4, complement protein C4; GBM, glomerular basement membrane; IgA, immunoglobulin A; IgG, immunoglobulin G; IgM, immunoglobulin M; TBM, tubular basement membrane.

a Categorical variables are expressed as count/total count of available entries, percent of total count (%).

The eGFR at presentation in FLD and FED patients was comparable, although the age at determination was significantly lower in FLD compared with FED patients (Table 6). For 54 of the FLD patients, eGFR from only one time point was available and showed a high variability in the degree of renal function impairment with median eGFR of 77.5 ml/min/1.73 m^2^ (IQR: 14.0; 92.5). For the remaining 46 FLD patients, two or more eGFR determinations were reported. In this subgroup, the median eGFR at first report was normal (82 ml/min/1.73 m^2^) but declined rapidly over time, with a median annual change in eGFR of −6.18 ml/min/1.73 m^2^ (Table 6).

**Table 6:** eGFR in FLD and FED patients

eGFR Parameters	Patient's Clinical Phenotype		FED Versus FLD^a^ *P*
	FLD	FED	
eGFR at presentation/first report (patients with one or more eGFR determinations)			
eGFR at presentation/first report (ml/min/1.73 m^2^)			0.2737
Median	81.5	90.0	
IQR	46.0; 98.0	84.0; 90.0	
N	100	15	
Age at presentation/first report (years)			0.0011
Median	34.5	55	
IQR	26.5; 46.0	43.0; 62.0	
N	100	15	
eGFR in FLD patients with one eGFR determination			
eGFR (ml/min/1.73 m^2^)		—	—
Median	77.5		
IQR	14.0; 92.5		
N	54		
Age at presentation/first report (years)		—	—
Median	35.0		
IQR	27.0; 48.0		
N	54		
eGFR and eGFR change in FLD patients with more than one eGFR determination			
eGFR at presentation/first report (ml/min/1.73 m^2^)		—	—
Median	82		
IQR	59.0; 100.0		
N	46		
eGFR at last report (ml/min/1.73 m^2^)		—	—
Median	14.0		
IQR	14.0; 58.0		
N	46		
Age at presentation/first report (years)		—	—
Median	33.5		
IQR	26.0; 45.0		
N	46		
Age at last report (years)		—	—
Median	41.3		
IQR	33.0; 53.0		
N	46		
Annual rate of eGFR change (ml/min/1.73 m^2^/year)		—	—
Median	−6.18		
IQR	−2.29; −8.93		
N	46		
Period of eGFR change (years)		—	—
Median	6.00		
IQR	2.5; 12.0		
N	46		

a Wilcoxon rank sum test.

### Cardiovascular disease

Data from the limited number of records describing individual patients suggest that FLD patients have lower prevalence of ASCVD, compared with FED patients, but comparable prevalence of preclinical ASCVD and CVD of other nature (Table 3). Additional studies reporting on ASCVD risks in larger groups of carriers of *LCAT* variants are listed in supplemental Table S7. These studies could not be included in the quantitative analysis because the study populations were genetically heterogenous and partially redundant across studies. Overall, data from the latter studies suggest that populations enriched in carriers of FED-causing variants display similar or increased risk of ASCVD as compared with noncarriers, whereas those enriched in carriers of FLD-causing variants display a decreased risk of ASCVD.

### Lipid profile and LCAT-related parameters

In order to evaluate potential diagnostic and prognostic biomarkers, we tested the association of lipid and LCAT-related parameters with the clinical/genetic phenotype.

As expected, HDL-C and Apo A–I, the primary HDL proteins, were markedly decreased in both FLD and FED patients (Table 4). Triglyceride (TG) levels were elevated in both groups. LDL-C and apoB levels were significantly lower in FLD compared with FED. Additional lipid and lipoprotein parameters for which data were available for less than 25% of the studied FLD and FED patients are shown in supplemental Table S8. Interestingly, levels of ApoA-II, another HDL protein, were also markedly reduced. The presence of LpX was reported in 32 of the 36 FLD patients where measured.

We extracted data for LCAT plasma concentration and activity. The “LCAT activity toward exogenous substrate” measures the ability of LCAT to esterify cholesterol in HDL-like particles, and it is therefore a marker of alpha activity. The “LCAT activity toward endogenous lipoproteins” measures the ability of LCAT to esterify cholesterol in all plasma lipoproteins, thus representing a combined measurement of both alpha and beta activity (total LCAT activity). Given the wide range of method used, we reported LCAT protein concentration and activities as percent of control values (Table 4).

Plasma LCAT concentration was reduced in both FLD and FED, albeit more markedly in FLD. LCAT activity toward exogenous substrate (alpha activity) was 1.4% (IQR: 0; 4.8) and 5.8% (IQR: 2.1; 9.5) of control values in FLD and FED, respectively (Table 4). LCAT activity toward endogenous lipoproteins (total, alpha, and beta activities) was virtually absent in FLD and 53.6% (IQR: 41.5; 69.6) of control levels in FED. These latest results were reflected in the levels of markers of plasma cholesterol esterification, namely the absolute levels of plasma UC (substrate of LCAT reaction) and the relative amount of EC (product of LCAT reaction) compared with total cholesterol (TC) (EC%).

Plasma UC was approximately 2-fold higher than the reference threshold in FLD patients (118.5 [IQR: 87; 161.7], reference <60 mg/dl) and approximately 1.2-fold higher in FED patients (74 [IQR: 61.2; 88.9], reference <60 mg/dl) (Table 4). Plasma EC% (percent of EC:TC) was approximately 5.4-fold below the lowest limit of the reference range in FLD (11, [IQR: 7.2; 16.2], reference: 60–80%) but only approximately 1.1-fold below the reference values in FED (54 [IQR: 43; 61.1], reference: 60–80%) (Table 4). All esterification markers were significantly different in FLD compared with FED, also after adjustment for plasma LCAT concentration (supplemental Table S9). Thus, in FLD, the complete loss of LCAT activity is explained by the presence of inactive LCAT and not only by reduced LCAT levels.

FLD and FED heterozygous subjects displayed a semidominant trait in respect to the major lipoprotein classes, and their levels of LCAT activities are somewhat lower than those of controls; however, the markers of plasma cholesterol esterification, absolute UC levels, and EC% were within normal limits (Table 4).

**Fig. 2:**
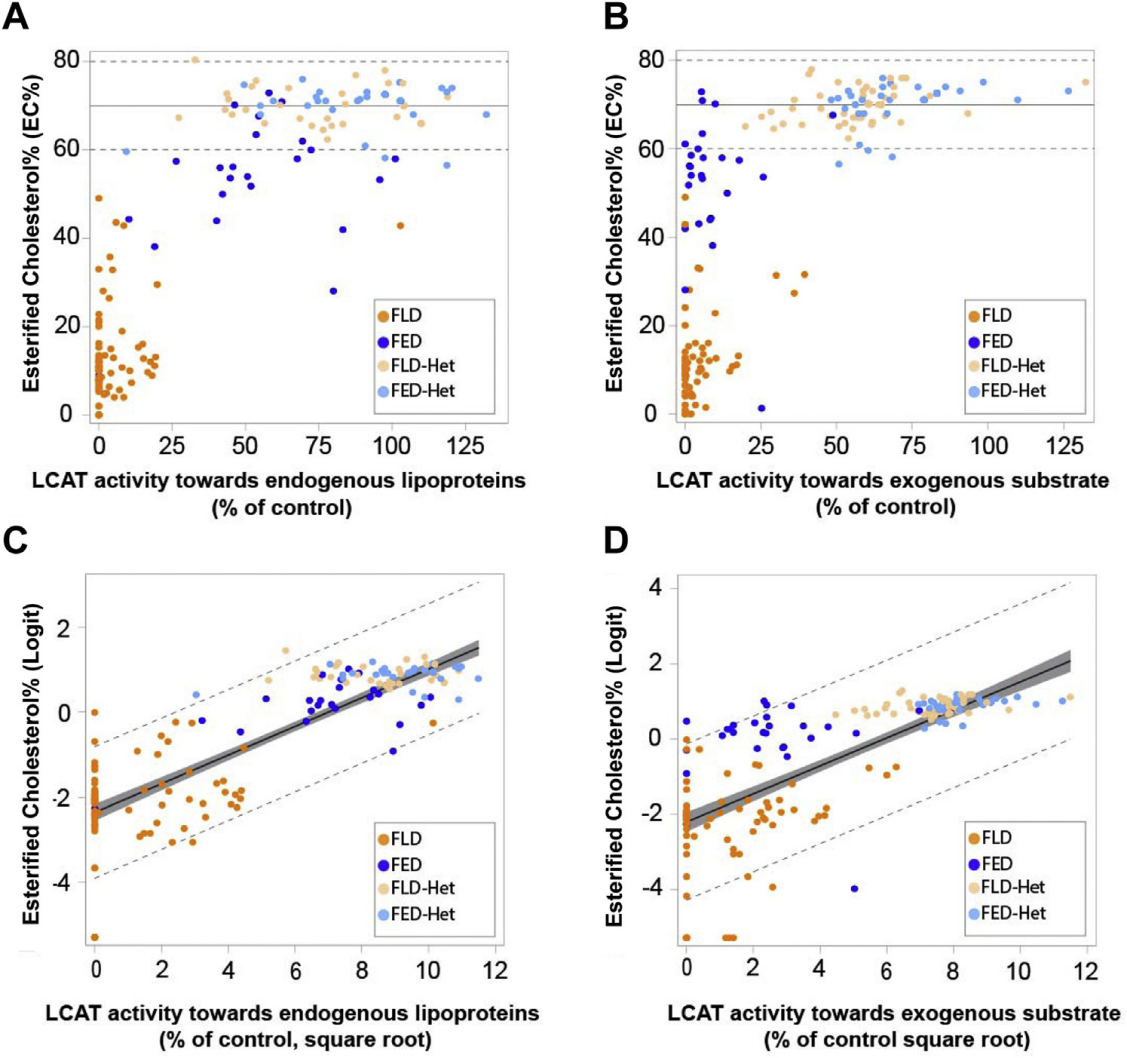
Association of LCAT activity on endogenous or exogenous substrates with EC%. A and B: Scatter plot representation of the association between LCAT activity toward endogenous lipoproteins (A) or exogenous substrates (B) and EC%. For both activity measures, levels are expressed as percent of the mean control value from the same study. Dashed lines indicate the limits of reference range values for EC% in nonaffected subjects. Solid line indicates the mean value of the reference range (70%). C and D: Linear regression analysis of the LCAT activity toward endogenous lipoproteins (C) or exogenous substrates (D) and EC%. Activity levels (percent of control) were root-squared transformed, and EC% levels were logit transformed prior to analysis. Dashed lines indicate 95% prediction limits, solid line indicates regression line, and area highlighted in gray represents 95% confidence limits. For all panels, single points represent values from a single subject, and color code reflects their classification based on clinical/genetic characteristics. Regression parameters were as follows: (C) number of subjects = 200, estimate equation: y = 0.3365 × −2.3594, *R*^2^ = 0.7616 (D) number of subjects = 214, estimate equation: y = 0.3729 × −2.2059, *R*^2^ = 0.6092.

Because EC% was the most differentiating indirect biomarker of cholesterol esterification, we evaluated its association with LCAT activity measurements. EC% displayed a nonlinear association with both LCAT activity toward endogenous (total, alpha, and beta activities) and exogenous substrates (alpha activity) across all subjects included in this study (Fig. 2A,C and B,D, respectively). Specifically, it shows a rapid increase in the lower range of LCAT activity, that tapers and reaches a plateau at higher levels of LCAT activity, so that 47.6% of enzymatic activity toward exogenous substrate and 65.7% toward endogenous substrate, respectively, were sufficient to achieve EC% levels of 70%, corresponding to the mean value of the reference range.

### Concordance between clinical and biochemical diagnosis

We examined the association between variants, clinical phenotype, and enzymatic activity in homozygotes and compound heterozygous subjects with the intent to assess the concordance between the clinical and biochemical diagnoses (supplemental Tables S10 and S11). Variants associated with the FLD phenotype were associated with complete loss of patient's plasma enzymatic activity in 80.4% of the cases and with partial enzymatic deficiency in 3.6% of the cases.

In contrast, variants associated with the FED phenotype were associated with complete enzymatic deficiency in 14.3% of cases and partial enzymatic deficiency in 60.7% of cases. For the remaining variants, there were not sufficient data for this assessment. When available, expression of variants in in vitro cell systems mostly recapitulated the biochemical diagnosis obtained by testing the patient's plasma (supplemental Tables S10 and S11). These results are in line with the known presence of a broad phenotypic variability and overlap between the two syndromes and highlight the need of further studies to identify potential modulators of the clinical course of these conditions.

### Biomarkers of renal disease severity and eGFR decline in FLD patients

We used multivariate regression analyses to test whether markers of LCAT activity or altered lipid metabolism were associated with the prevalence of severe renal disease (CKD stage III–V/ESRD) in FLD patients (Table 7). For the small subset of FLD patients for whom two or more eGFR determinations were available, we also estimated the potential association of candidate biomarkers with eGFR change (Table 7). After controlling for sex and age, higher levels of TC, non-HDL-C, and UC were significantly associated with higher prevalence of severe renal disease, and higher LDL-C levels were associated with a more pronounced eGFR decrease. Levels of EC% were not associated with prevalence of severe renal disease or eGFR decline.

**Table 7:** Univariate and multivariate regression analyses of parameters associated with the prevalence of severe CKD or eGFR change in FLD patients

Parameter	Univariate Analysis		Multivariate Analysis			
	Estimate Coefficient (SE), N	*P*	Model 1 (Sex)	*P*	Model 2 (Sex and Age)	*P*
			Estimate Coefficient (SE), N		Estimate Coefficient (SE), N	
Logistic regression analysis of parameters associated with CKD stage III–V/ESRD (log odds ratios)^a^						
TC (mg/dl)	0.0125 (0.0048), 78	0.0090	0.0120 (0.0046), 78	0.0090	0.0118 (0.0046), 78	0.0103
LDL-C (mg/dl)	0.0143 (0.0079), 45	0.0698	0.0165 (0.0084), 45	0.0503	0.0146 (0.0082), 45	0.0727
HDL-C (mg/dl)	0.0301 (0.0424), 67	0.4785	0.0325 (0.0427), 67	0.4467	0.0262 (0.0434), 67	0.5453
Non-HDL-C (mg/dl)	0.0148 (0.0059), 65	0.0125	0.0142 (0.0058), 65	0.0145	0.0138 (0.0058), 65	0.0184
UC (mg/dl)	0.0099 (0.0052), 57	0.0555	0.0105 (0.0052), 57	0.0443	0.0103 (0.0052), 57	0.0485
TGs (mg/dl)	0.0022 (0.0012), 77	0.0609	0.0020 (0.0011), 77	0.0800	0.0019 (0.0011), 77	0.1046
ApoB (mg/dl)	−0.0005 (0.0122), 49	0.9685	−0.0023 (0.0125), 49	0.8563	−0.0104 (0.0140), 49	0.4576
EC (% of TC)	0.0188 (0.0240), 62	0.4333	0.0100 (0.0245), 62	0.6827	0.0105 (0.0245), 62	0.6683
Linear regression analysis of parameters associated with eGFR change^b^						
TC (mg/dl)	−0.0209 (0.0144), 40	0.1552	−0.0221 (0.0129), 40	0.0945	−0.0233 (0.0127), 40	0.0750
LDL-C (mg/dl)	−0.0912 (0.0378), 19	0.0273	−0.0833 (0.0302), 19	0.0140	−0.0810 (0.0310), 19	0.0197
HDL-C (mg/dl)	−0.0957 (0.3024), 33	0.7539	−0.0286 (0.2568), 33	0.9122	−0.1993 (0.2726), 33	0.4706
Non-HDL-C (mg/dl)	−0.0329 (0.0209), 32	0.1252	−0.0229 (0.0181), 32	0.2166	−0.0278 (0.0178), 32	0.1290
UC (mg/dl)	−0.0154 (0.0187), 26	0.4205	−0.0216 (0.0157), 26	0.1829	−0.0234 (0.0161), 26	0.1604
TGs (mg/dl)	−0.0091 (0.0065), 40	0.1689	−0.0046 (0.0061), 40	0.4592	−0.0058 (0.0061), 40	0.3510
ApoB (mg/dl)	0.0330 (0.0980), 20	0.7406	0.0573 (0.0835), 20	0.5017	−0.0388 (0.0819), 20	0.6425
EC (% of TC)	0.1304 (0.2292), 26	0.5749	0.2340 (0.1941), 26	0.2402	0.2270 (0.1985), 26	0.2652

a Nonstandardized regression coefficients refer to the change in log odds of CKD stage III–V/ESRD, per unit increase in the levels of indicated parameters.

b Nonstandardized regression coefficients refer to the change in eGFR, per unit increase in the levels of indicated parameters.

## Discussion

We present an up-to-date comprehensive systematic review of patients with partial and complete LCAT deficiency. We reviewed reports published from 1967 until April 22, 2020 with the goal of gathering data on the natural history of FLD, with particular attention to the progression of renal disease and the evaluation of previously proposed biomarkers that could be used for diagnosis, assessing prognosis, and evaluating the efficacy of therapeutic interventions. The assessment of such biomarkers in a larger number of subjects is necessary to further the research toward the development of targeted therapeutic strategies.

The majority of the FLD and FED cases reported in the literature are from Europe. Although this observation may underlie a genuine enrichment in mutation frequency in certain populations (12), it likely reflects reporting, publication, and search bias. In recent years, new cases have been identified in Asian and South American countries (13, 14, 15, 16, 17, 18, 19), suggesting that this disease may be underdiagnosed, especially in isolated communities (13). The gender distribution of FLD cases is also imbalanced, with FLD patients being predominantly male. Although we cannot exclude a biological explanation for this outcome, the absence of such imbalance in the heterozygous subjects does not indicate an obvious sex-related pattern of inheritance. It is, nevertheless, possible that male sex may constitute an additional risk factor for progression to ESRD (20).

Diffuse corneal opacity, in association with extremely low HDL-C levels, is pathognomonic for both forms of LCAT deficiency. It is often first noted during childhood or adolescence but frequently not investigated until it requires surgical intervention or until the first signs of renal disease appear (FLD). Contributing to the late diagnosis is the low awareness of LCAT deficiency as a monogenic cause of nephrotic syndrome, possibly because of its rarity, because most studies focus on pediatric or young adult cohorts (21, 22), who are likely still asymptomatic for FLD-induced renal disease, and because the LCAT gene may not have sufficient coverage in the platforms used (23).

Our data confirm that the first sign of renal disease in FLD is proteinuria or hematuria, which may remain as the only signs for several years before a rapid decline of renal function is noted. About 53.4% of the FLD cases reported in the literature developed CKD stage III–V, by a median age of 38 years. This systematic review confirms that the variability of the renal disease progression was heterogenous across the FLD patients, with 15 patients remaining asymptomatic at time of publication and with some discordancy noted between the clinical and biochemical diagnosis, suggesting that other known and unknown factors, such as age, gender, comorbidities, and lipid levels, may contribute to the observed outcomes (supplemental Table S4). Furthermore, because of such variability, it is possible that some subjects who were classified as FED in this systematic review because of the reported phenotype may have developed renal disease after the time of publication. Nevertheless, in patients for whom we could calculate eGFR changes over time, a rapid decline at a rate of −6.18 ml/min/1.73 m^2^ was noted. This decline was greater than that reported recently in one large Greek kindred (−3.56 ml/min/1.73), which is also included in this study (24). It is possible that the overall eGFR data reported in our review were collected in a more advanced renal disease state compared with the cohort observed in that study. Furthermore, the therapeutic interventions, such as low-fat diet and angiotensin-converting enzyme inhibitors, attempted in that kindred may have somewhat mitigated disease progression. Nevertheless, the fast eGFR decline underscores the tremendous unmet medical needs of a condition that currently does not have effective diagnostic and therapeutic approaches to significantly alter its course.

FLD also displays a higher prevalence of anemia, although this observation was based on a very limited number of cases. Anemia is thought to be directly related to the deficiency of enzymatic activity. The complete loss of cholesterol esterification impairs the removal of UC from the membrane of erythrocytes, thus causing increased fragility and hemolytic anemia (25, 26, 27, 28). Our data suggest that the presentation of anemia almost coincides with the first signs of renal disease. Since anemia is often asymptomatic, it is unclear whether the age at first report reflects its onset or the date it was discovered through incidental laboratory testing. Further studies are needed to better evaluate the role of anemia as a biomarker of the condition.

This systematic review allows us to evaluate lipid biomarkers in a bigger number of cases than before reported. While most of these data are confirmatory, other data provide useful information for the planning of clinical trials. As expected, HDL-C was reduced to similar levels in both FLD and FED patients, reflecting the marked reduction in alpha activity in both conditions.

TG levels were elevated in both FLD and FED patients. The reasons for this observation are not completely understood. There have been reports of impaired TG-lipase activity in these patients (29, 30, 31, 32) as well as observations that the abnormal lipid phenotype is sensitive to dietary habits (33, 34, 35, 36, 37). It is also plausible that the virtual absence (FLD) or significant reduction (FED) of EC in the circulation may in turn impair the transfer of TG and EC between lipoproteins (38).

As previously reported, LDL-C and apoB levels were significantly decreased in FLD compared with FED, likely because of the absence of residual β-LCAT activity in FLD but not FED patients. Although these differences were statistically significant, the levels of these parameters were very variable across subjects, thus making them less suitable for diagnostic purposes. However, the levels of ApoB-containing lipoproteins may affect the risk of developing additional comorbidities, such as CVD. Indeed, our study suggests that FLD patients have reduced prevalence of ASCVD, compared with FED patients. These results are consistent with studies performed on Italian subjects (mostly FLD patients or FLD-heterozygous carriers), which showed atheroprotection compared with controls (4, 39, 40). Conversely, studies performed in Dutch subjects (mostly FED patients or FED-heterozygous carriers) indicated increased risk of CVD in carriers compared with controls (5, 6, 41, 42). Notably, results from a recent comparative study mainly focusing on heterozygous carriers form these two cohorts showed that FLD-Het had lower LDL-C and lower prevalence of preclinical atherosclerosis compared with FED-Het and controls (3). Although some carriers of biallelic variants (FLD and FED patients) were included in the latter study, their number was not sufficient to perform a comparative analysis of ASCVD risk in FLD and FED patients as separate groups (3).

For years, LCAT activity levels have been used to perform the differential diagnosis of FLD and FED in specialized research laboratories (7). Our data confirm that LCAT-alpha activity is drastically reduced in both FLD and FED and does not represent a differentiating factor between the two syndromes. Conversely, total LCAT activity (alpha and beta activity) is virtually absent in FLD patients, approximately half of normal in FED patients, and minimally affected in heterozygous subjects. These data confirm that the measurement of total LCAT enzymatic activity measured using endogenous substrate represents the most discriminatory diagnostic assay. Our data also provide for the first time an estimate threshold of LCAT activity levels (toward both endogenous and exogenous substrates) in FLD, FED, and heterozygous subjects, which is based on a large number of observations.

The measurement of LCAT activity can only be performed in specialized research laboratories and is not easily implemented in a clinical setting. We evaluated whether other indirect markers of cholesterol esterification, which are more easily measurable in a clinical laboratory, could differentiate between phenotypes. Both UC and EC% are proportionally affected by the number of mutated alleles. EC% is a measure of the relative abundance of EC compared with TC. As such, this measure showed the highest magnitude of difference across clinical phenotypes, and it displayed a significant correlation with total LCAT activity in plasma. For example, the median levels of LCAT activity toward endogenous substrate (percent of control value) were increased by ∼54, 86, and 98 in FED, FLD-het, and FED-Het, respectively, compared with FLD. In parallel, the median levels of EC (percent of TC) were increased by ∼43, 59, and 62 in FED, FLD-het, and FED-Het, respectively, compared with FLD.

Conversely, absolute UC levels are influenced by the levels of TC, and they show more overlapping between FLD and FED patients. Therefore, based on the data from this systematic review, EC% represents the most promising diagnostic biomarker in the clinical setting.

Interestingly, the correlation between LCAT activity and EC% is nonlinear, and normalization of EC% levels appear to be reached also in presence of subnormal levels of enzymatic activity. Specifically, ∼66% of normal LCAT activity toward endogenous substrate (total, alpha, and beta activities) and ∼48% of LCAT activity toward exogenous substrate (alpha activity) were sufficient to achieve normal levels of EC%.

This observation suggests that restoring relatively low levels of LCAT activity, perhaps comparable to those seen in FED patients, may be sufficient to prevent renal disease and poses the basis for the determination of a “therapeutic” threshold of activity that may be used as clinical endpoint.

It has been proposed that the renal disease in FLD patients is caused by abnormal circulating lipoproteins, such as LpX and abnormal large LDL particles (8, 43, 44, 45, 46, 47). LpX is an abnormal lipoprotein that is believed to originate from the nonspecific assembling of lipids (mostly phospholipids and UC) and proteins derived from the catabolism and remodeling of other circulating lipoproteins, such as chylomicrons and VLDL (45, 46, 48). Our analysis shows that LpX is in fact identified in 88.9% of the FLD patients tested for it. In mice and cells, LpX was shown to be taken up by glomerular endothelial cells, podocytes, and mesangial cells and induce inflammatory response (8), supporting a direct role for LpX in the development of renal damage (8, 45, 46). However, there have been isolated reports of patients with LpX and no renal disease (49, 50) as well as patients with renal disease and no LpX (47, 51). Furthermore, the presence of LpX in FED subjects has been rarely investigated (47), thus making it difficult to conclude whether this abnormal lipoprotein is specific of FLD. Large LDL particles with peculiar enrichment in TG and UC but distinct from LpX have also been reported in FLD but not FED patients, suggesting that these lipoproteins may also have a role in the development of renal disease (47). The absence of a reliable method for the detection of LpX and other abnormal lipoproteins may explain the different results obtained in past investigations. More studies using comparable LpX detection techniques (52) and including both FLD and FED patients are needed to conclusively determine the role of LpX and other abnormal apoB-containing lipoproteins in the pathogenesis and progression of renal disease. Interestingly, an increased risk of nephropathy has been observed in some heterozygous subjects in the context of other comorbidity affecting the lipid profile, supporting the importance of apoB-containing lipoprotein in modulating the renal phenotype (24, 53).

We were interested in evaluating if an association existed between any of the lipid parameters and the prevalence of severe CKD (stage III–V/ESRD) and/or eGFR decline in FLD patients. In the small number of cases for which data were available, higher levels of TC, UC, and non-HDL-C were significantly associated with the higher prevalence of severe CKD, and higher LDL-C levels were positively associated with eGFR decrease. These results suggest that, although most of FLD patients eventually progresses to severe renal disease, increased levels of TC, UC, non-HDL-C, and LDL-C may contribute to and/or accelerate its progression. Although the association with serum TG levels did not reach statistical significance in our analysis, the significant association with non-HDL-C levels suggests a possible role of TG-rich lipoproteins in the progression of renal disease (24). Interestingly, these data also show that UC appears to be a better prognostic predictor of CKD severity compared with EC%. It is worth noting that because EC% closely reflects activity levels, it is significantly and similarly low across the spectrum of FLD patients. Therefore, small variations in EC% levels across different FLD subjects may not be sufficient to affect renal disease outcome.

Conversely, the absolute amount of UC proportionally increases with the levels of TC and may vary significantly across FLD patients. In line with these findings, a recent follow-up study of a well-characterized cohort of 18 FLD patients, included in this review, found that higher UC levels were associated with lower event-free survival (54). These data support the rationale that the UC burden may be directly linked to the pathogenesis of renal damage, possibly by promoting the formation of LpX. These data are also consistent with anecdotal reports that lipid-lowering drugs improve the clinical phenotype of some patients (55, 56).

Renal biopsy reports showed heterogeneous findings, likely explained by the variable stage of renal damage across patients. Nevertheless, this systematic review confirmed the presence of common features that are unique to LCAT deficiency, such as the presence of lipid infiltrates in the glomerular basement membrane, pericapillary, and mesangial space and the sporadic presence of pseudothrombi and nonspecific inflammatory changes. It is interesting to note that complement protein C3 and immunoglobulin M were noted in more than 60% and 50% of biopsies with immunohistochemistry analysis, respectively. Although the number of observations is limited, these data suggest a role of the immune system in the pathogenesis of renal disease in FLD and provides foundation to the anecdotal reports in LCAT-deficient patients of sudden onset or worsening of nephrotic syndrome after flu-like symptoms or infections (24, 50, 57, 58, 59, 60).

Current treatment for FLD is limited to symptomatic management of its sequelae, specifically targeted to protect kidney function (34, 56, 61, 62). Although kidney transplantation in some FLD patients restored their kidney function, histological signs of the reoccurrence of disease have been documented in most of the few cases reported. Thus, FLD is a rare condition with clearly unmet needs. Excitingly, several potential treatments are at different stages of preclinical and early clinical development (9). There is therefore an urgent need to expand our knowledge of the natural history of the disease and validate candidate biomarkers that can predict disease occurrence and severity and could potentially be used to monitor treatment efficacy.

We recognize the limitations of this systemic review. The selected studies were mainly case reports, family studies, or small population studies, published over 53 years. Accordingly, there was variability in methodology and measurements, and longitudinal data on the cases described were limited. Because of the scarcity of the studies and limited cases, it was not possible to perform a formal assessment of study quality and risk of bias. In order to reduce the possibility of selection bias, all the records that reported extractable outcomes were included. Because of the nature of the study, the classification of patients as FLD and FED was based on clinical presentation and genetic information at the time of report. We cannot exclude the possibility that some FLD patients who were still asymptomatic for renal disease at the time of publication may have been misclassified as FED. The outcome definitions and approximations are reported in supplemental methods (Section 2, Data extraction). Despite these limitations, this systematic review is an important step in summarizing the current evidence on the pathogenesis of this condition, and it has several clinical implications.

Our findings confirm that the direct measurement of total LCAT enzymatic activity and the assessment of easy-to-measure indirect markers of LCAT function (such as EC%) are suitable diagnostic biomarkers to differentiate the FED and FLD syndromes. Our findings also reinforce the concept that multiple biochemical alterations associated with reduced LCAT function may be involved in the pathogenesis of renal damage and its progression: UC, TC, non-HDL-C, and LDL-C seems to be potential prognostic mediators of renal disease severity or progression, thus providing rationale for their therapeutic management with existing lipid-lowering therapies. Furthermore, other factors unrelated to LCAT such as concomitant infections may precipitate the decline in kidney function in FLD and should require particular attention.

Finally, the observation that there is a strong and quantitative relationship between LCAT activity levels and severity of clinical phenotype suggests that there is a “therapeutic” level of enzymatic activity that may be sufficient to slow the progression or even prevent the onset of severe renal disease. These results offer promise in guiding the development of novel therapeutic approaches for LCAT deficiency. These findings will need to be confirmed in a larger natural history study that, given the rarity of this condition, will require a collaborative effort amongst the international community of LCAT researchers, clinicians, and patients.

## Data availability

All the study data are contained within this article and its supplemental data file.

## Supplemental data

This article contains supplemental data (63, 64, 65, 66, 67, 68, 69, 70, 71, 72, 73, 74, 75, 76, 77, 78, 79, 80, 81, 82, 83, 84, 85, 86, 87, 88, 89, 90, 91, 92, 93, 94, 95, 96, 97, 98, 99, 100, 101, 102, 103, 104, 105, 106, 107, 108, 109, 110, 111, 112, 113, 114, 115, 116, 117, 118, 119, 120, 121, 122, 123, 124, 125, 126, 127, 128, 129, 130, 131, 132, 133, 134, 135, 136, 137, 138, 139, 140, 141, 142, 143, 144, 145, 146, 147, 148, 149, 150, 151, 152, 153, 154, 155, 156, 157, 158, 159, 160, 161, 162, 163, 164, 165, 166, 167, 168, 169, 170, 171, 172, 173, 174, 175, 176, 177, 178, 179, 180, 181, 182, 183, 184, 185, 186, 187, 188, 189, 190, 191, 192, 193, 194, 195, 196).

## Conflict of interest

The authors declare that they have no conflicts of interest with the contents of this article.
