# Supplementary material for: A systematic review of the natural history and biomarkers of primary lecithin:cholesterol acyltransferase deficiency
Source: J Lipid Res. 2022 Jan 20;63(3):100169. doi: 10.1016/j.jlr.2022.100169 (PMC8953693; doi:10.1016/j.jlr.2022.100169)
Supplement: Supplemental Tables S1–S11, Figure S1 and Methods [file mmc1.pdf]

Supplemental Material

Title

A systematic review of the natural history and biomarkers of primary Lecithin:Cholesterol Acyltransferase (LCAT) deficiency

Authors

Cecilia Vitali[1]

Archna Bajaj[1]

Christina Nguyen[1]

Jill Schnall[2]

Jinbo Chen[1,2]

Kostas Stylianou[3]

Daniel J. Rader[1,4]

Marina Cuchel[1]

Affiliations:

[1] Department of Medicine, Division of Translational Medicine and Human Genetics, Perelman School of Medicine, University of Pennsylvania, Philadelphia, PA, USA

[2] Department of Biostatistics, Epidemiology and Informatics, University of Pennsylvania, Philadelphia, PA, USA

[3] Department of Nephrology, Heraklion University Hospital, Crete, Greece

[4] Department of Genetics, Perelman School of Medicine, University of Pennsylvania, Philadelphia, PA, USA

Content

Table S1. Characteristics of included studies 3
Table S2. List of variants identified in at least one homozygous subject 16
Table S3. List of variants only identified in heterozygous and compound heterozygous subjects. ... 18
Table S4. Unclassified Subjects 20
Table S5. Transplantation outcome at follow up 23
Table S6. Kidney Histologic findings in single FLD patients 24
Table S7. Summary of observational studies of CVD risk in FLD and FED patients and heterozygous subjects. 27
Table S8. Additional lipid and lipoprotein parameters 30
Table S9. Linear Regression Analysis of LCAT activity, EC% and UC levels with FLD/FED phenotypes and LCAT concentration. 31
Table S10. Association of clinical phenotype with predicted and observed enzymatic activity in homozygous subjects 32
Table S11. Association of clinical phenotype with predicted and observed enzymatic activity in compound heterozygous subjects 34
Figure S1: Geographical distribution of FLD and FED cases included in this study 37
Supplemental methods 38
  1. Record search and selection 38
  2. Data extraction. 39
  3. Synopsis of the subject classification protocol. 44
  5. Regression analyses. 45
References (supplemental material) 47

Table S1. Characteristics of included studies

Record number	Record	Data collected	Population studied	Probands' Clinical phenotype
1	Adimoolam, S., L. Jin, E. Grabbe, J. J. Shieh, and A. Jonas. (1998) Structural and functional properties of two mutants of lecithin-cholesterol acyltransferase (T123I and N228K). The Journal of biological chemistry 273: 32561-32567.	Characterization of recombinant variants	NA	NA
2	Ahmad, S. B., M. Miller, S. Hanish, S. T. Bartlett, W. Hutson, R. N. Barth, and J. C. LaMattina. (2016) Sequential kidney-liver transplantation from the same living donor for lecithin cholesterol acyl transferase deficiency. Clinical transplantation 30: 1370-1374.	Clinical and Laboratory data	Case	FLD
3	Albers, J. J., and G. Utermann. (1981) Genetic control of lecithin-cholesterol acyltransferase (LCAT): measurement of LCAT mass in a large kindred with LCAT deficiency. Am J Hum Genet 33: 702-708.	Clinical and Laboratory data	Family study	FLD
4	Albers, J. J., C. Chen, and J. L. Adolphson. (1981) Familial lecithin-cholesterol acyltransferase: identification of heterozygotes with half-normal enzyme activity and mass. Hum Genet 58: 306-309.	Laboratory data	Family	FLD
5	Albers, J. J., C. H. Chen, J. Adolphson, M. Sakuma, T. Kodama, and Y. Akanuma. (1982) Familial lecithin-cholesterol acyltransferase deficiency in a Japanese family: evidence for functionally defective enzyme in homozygotes and obligate heterozygotes. Hum Genet 62: 82-85.	Clinical and Laboratory data	Family	FED
6	Albers, J. J., E. Gjone, J. L. Adolphson, C. H. Chen, P. Teisberg, and H. Torsvik. (1981) Familial lecithin-cholesterol acyltransferase deficiency in four Norwegian Families. Evidence for low levels of a functionally defective enzyme. Acta Med Scand 210: 455-459.	Laboratory data	Family	FLD
7	Albers, J. J., J. Adolphson, C. H. Chen, N. Murayama, S. Honma, and Y. Akanuma. (1985) Defective enzyme causes lecithin-cholesterol acyltransferase deficiency in a Japanese kindred. Biochimica et biophysica acta 835: 253-257.	Clinical and Laboratory data	Family	FLD
8	Althaf, M. M., H. Almana, A. Abdelfadiel, S. M. Amer, and T. O. Al-Hussain. (2015) Familial lecithin-cholesterol acyltransferase (LCAT) deficiency; a differential of proteinuria. Journal of nephropathology 4: 25-28.	Clinical and Laboratory data	Case	FLD
9	Angelin, B., and L. A. Carlson. (1986) Bile acids and plasma high density lipoproteins: biliary lipid metabolism in fish eye disease. Eur J Clin Invest 16: 157-162.	Laboratory data	Case	FED
10	Aranda, P., P. Valdivielso, L. Pisciotta, I. Garcia, A. A. C. Garcã, S. Bertolini, N. R. G. Martã, L.-S. Gonzã, and S. Calandra. (2008) Therapeutic management of a new case of LCAT deficiency with a multifactorial long-term approach based on high doses of angiotensin II receptor blockers (ARBs). Clinical nephrology 69: 213-218.	Clinical and Laboratory data	Case	FLD
11	Argyropoulos, G., A. Jenkins, R. L. Klein, T. Lyons, B. Wagenhorst, J. St Armand, S. M. Marcovina, J. J. Albers, P. H. Pritchard, and W. T. Garvey. (1998) Transmission of two novel mutations in a pedigree with familial lecithin:cholesterol acyltransferase deficiency: structure-function relationships and studies in a compound heterozygous proband. J Lipid Res 39: 1870-1876.	Clinical and Laboratory data	Family	FED
12	Asada, S., M. Kuroda, Y. Aoyagi, H. Bujo, S. Tanaka, S. Konno, M. Tanio, I. Ishii, M. Aso, and Y. Saito. (2011) Disturbed apolipoprotein A-I-containing lipoproteins in fish-eye disease are improved by the lecithin:cholesterol acyltransferase produced by gene-transduced adipocytes in vitro. Molecular genetics and metabolism 102: 229-231.	Clinical and Laboratory data	Family	FED
13	Assmann, G., A. von Eckardstein, and H. Funke. (1991) Lecithin: cholesterol acyltransferase deficiency and fish-eye disease. Current opinion in lipidology 2: 110-117.	Clinical and Laboratory data	Case collection	FLD; FED
14	Ayyobi, A. F., S. H. McGladdery, S. Chan, G. B. John Mancini, J. S. Hill, and J. J. Frohlich. (2004) Lecithin: cholesterol acyltransferase (LCAT) deficiency and risk of vascular disease: 25 year follow-up. Atherosclerosis 177: 361-366.	Clinical and Laboratory data	Family	FLD
15	Baass, A., H. Wassef, M. Tremblay, L. Bernier, R. Dufour, and J. Davignon. (2009) Characterization of a new LCAT mutation causing familial LCAT deficiency (FLD) and the role of APOE as a modifier gene of the FLD phenotype. Atherosclerosis 207: 452-457.	Clinical and Laboratory data	Family	FLD

16	Balwani, M. R., V. A. Ghodela, V. B. Kute, P. R. Shah, H. V. Patel, D. N. Gera, A. Vanikar, and H. L. Trivedi. (2017) An unusual presentation of LCAT deficiency as nephrotic syndrome with normal serum HDL-C level. Journal of nephropharmacology 6: 23-26.	Clinical and Laboratory data	Case	Unclassified
17	Bender, B. U., T. Quaschning, H. P. Neumann, D. Schmidt, and A. Kraemer-Guth. (2007) A novel frameshift mutation of the lecithin:cholesterol acyltransferase (LCAT) gene associated with renal failure in familial LCAT deficiency. Clinical chemistry and laboratory medicine 45: 483-486.	Clinical and Laboratory data	Family	FLD
18	Berard, A. M., M. Clerc, B. Brewer, Jr., and S. Santamarina-Fojo. (2001) A normal rate of cellular cholesterol removal can be mediated by plasma from a patient with familial lecithin-cholesterol acyltransferase (LCAT) deficiency. Clinica chimica acta; international journal of clinical chemistry 314: 131-139.	Clinical and Laboratory data	Case	FLD
19	Bethell, W., C. McCulloch, and M. Ghosh. (1975) Lecithin cholesterol acyl transferase deficiency. Light and electron microscopic finding from two corneas. Canadian journal of ophthalmology. Journal canadien d'ophtalmologie 10: 494-501.	Clinical and Laboratory data	Family	FLD
20	Blanco-Vaca, F., S. J. Qu, C. Fiol, H. Z. Fan, Q. Pao, A. Marzal-Casacuberta, J. J. Albers, I. Hurtado, V. Gracia, X. Pinto, T. Marti, and H. J. Pownall. (1997) Molecular basis of fish-eye disease in a patient from Spain. Characterization of a novel mutation in the LCAT gene and lipid analysis of the cornea. Arterioscler Thromb Vasc Biol 17: 1382-1391.	Clinical and Laboratory data; Characterization of recombinant variants	Family	FED
21	Blomhoff, J. P., R. Holme, J. Sauar, and E. Gjone. (1978) Familial lecithin:cholesterol acyltransferase deficiency. Further studies on plasma lipoproteins and plasma postheparin lipase activity of a patient with normal renal function. Scandinavian journal of clinical and laboratory investigation. Supplementum 150: 177-182.	Laboratory data	Case	FLD
22	Borysiewicz, L. K., A. K. Soutar, D. J. Evans, G. R. Thompson, and A. J. Rees. (1982) Renal failure in familial lecithin: cholesterol acyltransferase deficiency. The Quarterly journal of medicine 51: 411-426.	Clinical and Laboratory data	Family	FLD
23	Bron, A. J., J. K. Lloyd, A. S. Fosbrooke, A. F. Winder, and R. C. Tripathi. (1975) Letter: Primary L.C.A.T.-deficiency disease. Lancet (London, England) 1: 928-929.	Clinical and Laboratory data	Case	FLD
24	Bujo, H., J. Kusunoki, M. Ogasawara, T. Yamamoto, Y. Ohta, T. Shimada, Y. Saito, and S. Yoshida. (1991) Molecular defect in familial lecithin:cholesterol acyltransferase (LCAT) deficiency: a single nucleotide insertion in LCAT gene causes a complete deficient type of the disease. Biochemical and biophysical research communications 181: 933-940.	Clinical and Laboratory data	Case	FLD
25	Calabresi, L., D. Baldassarre, S. Castelnuovo, P. Conca, L. Bocchi, C. Candini, B. Frigerio, M. Amato, C. R. Sirtori, P. Alessandrini, M. Arca, G. Boscutti, L. Cattin, L. Gesualdo, T. Sampietro, et al. (2009) Functional lecithin: cholesterol acyltransferase is not required for efficient atheroprotection in humans. Circulation 120: 628-635.	Characterization of recombinant variants	NA	NA
26	Calabresi, L., L. Pisciotta, A. Costantin, I. Frigerio, I. Eberini, P. Alessandrini, M. Arca, G. B. Bon, G. Boscutti, G. Busnach, G. Frasca, L. Gesualdo, M. Gigante, G. Lupattelli, A. Montali, et al. (2005) The molecular basis of lecithin:cholesterol acyltransferase deficiency syndromes: a comprehensive study of molecular and biochemical findings in 13 unrelated Italian families. Arterioscler Thromb Vasc Biol 25: 1972-1978.	Clinical and Laboratory data	Collection of families	FLD; FED
27	Carlson, L. A. (1979) A further case of fish-eye disease. Lancet (London, England) 2: 1376-1377.	Laboratory data	Case collection	FED
28	Carlson, L. A. (1982) Fish eye disease: a new familial condition with massive corneal opacities and dyslipoproteinaemia. Eur J Clin Invest 12: 41-53.	Laboratory data	Case collection	FED
29	Carlson, L. A., and B. Philipson. (1979) Fish-eye disease. A new familial condition with massive corneal opacities and dyslipoproteinaemia. Lancet (London, England) 2: 922-924.	Laboratory data	Case collection	FED
30	Carlson, L. A., and L. Holmquist. (1983) Studies on high density lipoproteins in fish eye disease. Acta Med Scand 213: 177-182.	Clinical and Laboratory data	Family	FED
31	Carlson, L. A., and L. Holmquist. (1985) Evidence for deficiency of high density lipoprotein lecithin: cholesterol acyltransferase activity (alpha-LCAT) in fish eye disease. Acta Med Scand 218: 189-196.	Clinical and Laboratory data	Case	FED
32	Carlson, L. A., and L. Holmquist. (1985) Paradoxical esterification of plasma cholesterol in fish eye disease. Acta Med Scand 217: 491-499.	Clinical and Laboratory data	Family	FED
33	Castro-Ferreira, I., R. Carmo, S. E. Silva, O. Correa, S. Fernandes, S. Sampaio, R. P. Pedro, A. Praca, and J. P. Oliveira. (2018) Novel Missense LCAT Gene Mutation Associated with an Atypical Phenotype of Familial LCAT Deficiency in Two Portuguese Brothers. JIMD reports 40: 55-62.	Clinical and Laboratory data	Family	FLD

34	Charlton-Menys, V., L. Pisciotta, P. N. Durrington, R. Neary, C. D. Short, L. Calabresi, S. Calandra, and S. Bertolini. (2007) Molecular characterization of two patients with severe LCAT deficiency. Nephrology, dialysis, transplantation : official publication of the European Dialysis and Transplant Association - European Renal Association 22: 2379-2382.	Clinical and Laboratory data	Case collection	FLD
35	Chevet, D., M. P. Ramée, P. Le Pogamp, R. Thomas, M. Garré, and L. G. Alcindor. (1978) Hereditary lecithin cholesterol acyltransferase deficiency. Report of a new family with two afflicted sisters. Nephron 20: 212-219.	Clinical and Laboratory data	Family	FLD
36	Cirera, S., J. Julve, I. Ferrer, C. Mainou, R. Bonet, J. M. Martin-Campos, F. Gonzalez-Sastre, and F. Blanco-Vaca. (1998) Molecular diagnosis of lecithin: cholesterol acyltransferase deficiency in a presymptomatic proband. Clinical chemistry and laboratory medicine 36: 443-448.	Clinical and Laboratory data	Family	Unclassified
37	Clerc, M., M. F. Dumon, D. Sess, M. Freneix-Clerc, M. Mackness, and C. Conri. (1991) A 'Fish-eye disease' familial condition with massive corneal opacities and hypoalphalipoproteinaemia: clinical, biochemical and genetic features. Eur J Clin Invest 21: 616-624.	Clinical and Laboratory data	Family	FED
38	Cogan, D. G., H. S. Kruth, M. B. Datilis, and N. Martin. (1992) Corneal opacity in LCAT disease. Cornea 11: 595-599.	Clinical and Laboratory data	Case	FED
39	Conca, P., S. Pileggi, S. Simonelli, E. Boer, G. Boscutti, L. Magnolo, P. Tarugi, S. Penco, G. Franceschini, L. Calabresi, and M. Gomaraschi. (2012) Novel missense variants in LCAT and APOB genes in an Italian kindred with familial lecithin:cholesterol acyltransferase deficiency and hypobetalipoproteinemia. *J Clin Lipidol* **6**: 244-250.	Clinical and Laboratory data	Family	Unclassified
40	Contacos, C., D. R. Sullivan, K. A. Rye, H. Funke, and G. Assmann. (1996) A new molecular defect in the lecithin: cholesterol acyltransferase (LCAT) gene associated with fish eye disease. *J Lipid Res* **37**: 35-44.	Clinical and Laboratory data	Family	FED
41	De Smedt, M., R. Van Ginderdeuren, R. De Vos, A. Mertens, E. Muls, and B. Foets. (2001) Corneal opacifications in a low high density lipoprotein syndrome: suspicion of fish eye disease: a case report. *Bulletin de la Societe belge d'ophtalmologie*: 25-30.	Clinical and Laboratory data	Case	FED
42	Dimick, S. M., B. Sallee, B. F. Asztalos, P. H. Pritchard, J. Frohlich, and E. J. Schaefer. (2014) A kindred with fish eye disease, corneal opacities, marked high-density lipoprotein deficiency, and statin therapy. *J Clin Lipidol* **8**: 223-230.	Clinical and Laboratory data	Family	FED
43	Dorval, I., P. Jezequel, C. Dubourg, B. Chauvel, P. Le Pogamp, and J. Y. Le Gall. (1994) Identification of the homozygous missense mutation in the lecithin:cholesterol-acyltransferase (LCAT) gene, causing LCAT familial deficiency in two French patients. *Atherosclerosis* **105**: 251-252.	Genetic data	Case	FLD
44	Elkhalil, L., Z. Majd, R. Bakir, O. Perez-Mendez, G. Castro, P. Poulain, B. Lacroix, N. Duhal, J. C. Fruchart, and G. Luc. (1997) Fish-eye disease: structural and in vivo metabolic abnormalities of high-density lipoproteins. *Metabolism: clinical and experimental* **46**: 474-483.	Laboratory data	Case	FED
45	Flatmark, A. L., T. Hovig, E. Myhre, and E. Gjone. (1977) Renal transplantation in patients with familial lecithin: cholesterol-acetyltransferase deficiency. *Transplant Proc* **9**: 1665-1671.	Clinical and Laboratory data	Case collection	FLD
46	Fotakis, P., J. A. Kuivenhoven, E. Dafnis, D. Kardassis, and V. I. Zannis. (2015) The Effect of Natural LCAT Mutations on the Biogenesis of HDL. *Biochemistry* **54**: 3348-3359.	Characterization of recombinant variants	NA	NA
47	Fountoulakis, N., E. Lioudaki, D. Lygerou, E. K. Dermitzaki, I. Papakitsou, V. Kounali, A. G. Holleboom, S. Stratigis, C. Belogianni, P. Syngelaki, S. Stratakis, A. Evangeliou, H. Gakiopoulou, J. A. Kuivenhoven, R. Wevers, et al. (2019) The P274S Mutation of Lecithin-Cholesterol Acyltransferase (LCAT) and Its Clinical Manifestations in a Large Kindred. *American journal of kidney diseases : the official journal of the National Kidney Foundation* **74**: 510-522.	Clinical and Laboratory data; Characterization of recombinant variants	Family	FLD
48	Frasca, G. M., L. Soverini, E. Tampieri, G. Franceschini, L. Calabresi, L. Pisciotta, P. Preda, A. Vangelista, S. Stefoni, and S. Bertolini. (2004) A 33-year-old man with nephrotic syndrome and lecithin-cholesterol acyltransferase (LCAT) deficiency. Description of two new mutations in the LCAT gene. *Nephrology, dialysis, transplantation : official publication of the European Dialysis and Transplant Association - European Renal Association* **19**: 1622-1624.	Clinical and Laboratory data	Family	FLD
49	Frohlich, J., G. Hoag, R. McLeod, M. Hayden, D. V. Godin, L. D. Wadsworth, J. D. Critchley, and P. H. Pritchard. (1987) Hypoalphalipoproteinemia resembling fish eye disease. *Acta Med Scand* **221**: 291-298.	Clinical and Laboratory data	Family	FLD
50	Frohlich, J., K. Hon, and R. McLeod. (1982) Detection of heterozygotes for familial lecithin: cholesterol acyltransferase (LCAT) deficiency. *Am J Hum Genet* **34**: 65-72.	Clinical and Laboratory data	Family	FED
51	Frohlich, J., R. McLeod, and K. Hon. (1982) Lecithin: cholesterol acyl transferase (LCAT). *Clinical biochemistry* **15**: 269-278.	Clinical and Laboratory data	Family	FLD

52	Frohlich, J., R. McLeod, P. H. Pritchard, J. Fesmire, and W. McConathy. (1988) Plasma lipoprotein abnormalities in heterozygotes for familial lecithin:cholesterol acyltransferase deficiency. *Metabolism: clinical and experimental* **37**: 3-8.	Laboratory data	Case collection	FLD; FED
53	Frohlich, J., W. J. Godolphin, C. E. Reeve, and K. A. Evelyn. (1978) Familial LCAT deficiency. Report of two patients from a Canadian family of Italian and Swedish descent. *Scand J Clin Lab Invest Suppl* **150**: 156-161.	Laboratory data	Family	FLD
54	Funke, H., A. von Eckardstein, P. H. Pritchard, A. E. Hornby, H. Wiebusch, C. Motti, M. R. Hayden, C. Dachet, B. Jacotot, U. Gerdes, and et al. (1993) Genetic and phenotypic heterogeneity in familial lecithin: cholesterol acyltransferase (LCAT) deficiency. Six newly identified defective alleles further contribute to the structural heterogeneity in this disease. *The Journal of clinical investigation* **91**: 677-683.	Clinical and Laboratory data	Family	FED
55	Funke, H., A. von Eckardstein, P. H. Pritchard, J. J. Albers, J. J. Kastelein, C. Droste, and G. Assmann. (1991) A molecular defect causing fish eye disease: an amino acid exchange in lecithin-cholesterol acyltransferase (LCAT) leads to the selective loss of alpha-LCAT activity. *Proc Natl Acad Sci U S A* **88**: 4855-4859.	Clinical and Laboratory data	Case collection	FLD
56	Geller, A. S., E. Y. Polisecki, M. R. Diffenderfer, B. F. Asztalos, S. K. Karathanasis, R. A. Hegele, and E. J. Schaefer. (2018) Genetic and secondary causes of severe HDL deficiency and cardiovascular disease. *J Lipid Res* **59**: 2421-2435.	Clinical and Laboratory data	Population	FLD; FED; Unclassified
57	Gigante, M., E. Ranieri, G. Cerullo, L. Calabresi, A. Iolascon, G. Assmann, L. Morrone, L. Pisciotta, F. P. Schena, and L. Gesualdo. (2006) LCAT deficiency: molecular and phenotypic characterization of an Italian family. *J Nephrol* **19**: 375-381.	Clinical and Laboratory data	Family	FLD
58	Gjone, E. (1974) Familial lecithin:cholesterol acyltransferase deficiency--a clinical survey. *Scand J Clin Lab Invest Suppl* **137**: 73-82.	Clinical and Laboratory data	Case	FLD
59	Gjone, E. (1981) Familial lecithin:cholesterol acyltransferase deficiency - a new metabolic disease with renal involvement. *Advances in nephrology from the Necker Hospital* **10**: 167-185.	Clinical and Laboratory data	Family	FLD
60	Gjone, E., A. J. Skarbovik, J. P. Blomhoff, and P. Teisberg. (1974) Familial lecithin:cholesterol acyltransferase deficiency. Report of a third Norwegian family with two afflicted members. *Scand J Clin Lab Invest Suppl* **137**: 101-105.	Laboratory data	Family	FLD
61	Gjone, E., and K. R. Norum. (1968) Familial serum cholesterol ester deficiency. Clinical study of a patient with a new syndrome. *Acta Med Scand* **183**: 107-112.	Clinical and Laboratory data	Family	FLD
62	Gjone, E., J. P. Blomhoff, and A. J. Skarbovik. (1974) Possible association between an abnormal low density lipoprotein and nephropathy in lecithin: cholesterol acyltransferase deficiency. *Clinica chimica acta; international journal of clinical chemistry* **54**: 11-18.	Clinical and Laboratory data	Family	FLD
63	Gjone, E., J. P. Blomhoff, R. Holme, T. Hovig, B. Olaisen, A. J. Skarbovik, and P. Teisberg. (1981) Familial lecithin:cholesterol acyltransferase deficiency. Report of a fourth family from northwestern Norway. *Acta Med Scand* **210**: 3-6.	Clinical and Laboratory data	Collection of families	FLD
64	Gjone, E., N. B. Javitt, J. P. Blomhoff, and O. Fausa. (1973) Studies of lipoprotein-X (LP-X) and bile acids in familial LCAT deficiency. Preliminary report. *Acta Med Scand* **194**: 377-378.	Clinical and Laboratory data	Collection of families	FLD
65	Glomset, J. A., K. R. Norum, A. V. Nichols, W. C. King, C. D. Mitchell, K. R. Applegate, E. L. Gong, and E. Gjone. (1975) Plasma lipoproteins in familial lecithin: cholesterol acyltransferase deficiency: effects of dietary manipulation. *Scand J Clin Lab Invest Suppl* **142**: 3-30.	Clinical and Laboratory data	Case collection	FLD
66	Gotoda, T., N. Yamada, T. Murase, M. Sakuma, N. Murayama, H. Shimano, K. Kozaki, J. J. Albers, Y. Yazaki, and Y. Akanuma. (1991) Differential phenotypic expression by three mutant alleles in familial lecithin:cholesterol acyltransferase deficiency. *Lancet (London, England)* **338**: 778-781.	Clinical and Laboratory data	Case collection	FLD; FED
67	Guerin, M., C. Dachet, S. Goulinet, D. Chevet, P. J. Dolphin, M. J. Chapman, and M. Rouis. (1997) Familial lecithin:cholesterol acyltransferase deficiency: molecular analysis of a compound heterozygote: LCAT (Arg147 --> Trp) and LCAT (Tyr171 --> Stop). *Atherosclerosis* **131**: 85-95.	Clinical and Laboratory data	Family	FLD
68	Guerin, M., P. J. Dolphin, and M. J. Chapman. (1993) Familial lecithin:cholesterol acyltransferase deficiency: further resolution of lipoprotein particle heterogeneity in the low density interval. *Atherosclerosis* **104**: 195-212.	Laboratory data	Case	FLD
69	Gylling, H., and T. A. Miettinen. (1992) Non-cholesterol sterols, absorption and synthesis of cholesterol and apolipoprotein A-I kinetics in a Finnish lecithin-cholesterol acyltransferase deficient family. *Atherosclerosis* **95**: 25-33.	Laboratory data	Family	FLD
70	Gylling, H., and T. A. Miettinen. (1993) Postabsorptive retinyl palmitate removal is retarded in lecithin-cholesterol acyltransferase deficiency. *Eur J Clin Invest* **23**: 302-306.	Laboratory data	Family	FLD
71	Haase, C. L., A. Tybjaerg-Hansen, A. A. Qayyum, J. Schou, B. G. Nordestgaard, and R. Frikke-Schmidt. (2012) LCAT, HDL cholesterol and ischemic cardiovascular disease: a Mendelian randomization study of HDL cholesterol in 54,500 individuals. *J Clin Endocrinol Metab* **97**: E248-256.	Clinical and Laboratory data	Population	Non FLD/FED

72	Hamnstrom, B. (1970) The lipemia in familial plasma lecithin: cholesterol acyltransferase deficiency. *Acta Med Scand* **1-2**: 55-58.	Clinical and Laboratory data	Family	FLD
73	Hamnstrom, B., E. Gjone, and K. R. Norum. (1969) Familial plasma lecithin: cholesterol acyltransferase deficiency. *British medical journal* **2**: 283-286.	Laboratory data	Family	FLD
74	Hanna, E. V., S. Simonelli, S. Chamney, A. Ossoli, and R. N. Mullan. (2018) Paradoxical fall in proteinuria during pregnancy in an LCAT-deficient patient-A case report. *J Clin Lipidol* **12**: 1151-1156.	Clinical and Laboratory data	Case	FLD
75	Hill, J. S., K. O, X. Wang, and P. H. Pritchard. (1993) Lecithin:cholesterol acyltransferase deficiency: identification of a causative gene mutation and a co-inherited protein polymorphism. *Biochimica et biophysica acta* **1181**: 321-323.	Characterization of recombinant variants	NA	NA
76	Hirano, K., S. Kachi, C. Ushida, and M. Naito. (2004) Corneal and macular manifestations in a case of deficient lecithin: cholesterol acyltransferase. *Japanese journal of ophthalmology* **48**: 82-84.	Clinical	Case	FLD
77	Hirashio, S., K. Izumi, T. Ueno, T. Arakawa, T. Naito, T. Taguchi, and N. Yorioka. (2010) Point mutation (C to T) of the LCAT gene resulting in A140C substitution. *Journal of atherosclerosis and thrombosis* **17**: 1297-1301.	Clinical and Laboratory data	Case	FLD
78	Holleboom, A. G., J. A. Kuivenhoven, C. C. van Olden, J. Peter, A. W. Schimmel, J. H. Levels, R. M. Valentijn, P. Vos, J. C. Defesche, J. J. Kastelein, G. K. Hovingh, E. S. Stroes, and C. E. Hollak. (2011) Proteinuria in early childhood due to familial LCAT deficiency caused by loss of a disulfide bond in lecithin:cholesterol acyl transferase. *Atherosclerosis* **216**: 161-165.	Clinical and Laboratory data; Characterization of recombinant variants	Population	FED; FLD-Het; FED-het
79	Holleboom, A. G., J. A. Kuivenhoven, F. Peelman, A. W. Schimmel, J. Peter, J. C. Defesche, J. J. Kastelein, G. K. Hovingh, E. S. Stroes, and M. M. Motazacker. (2011) High prevalence of mutations in LCAT in patients with low HDL cholesterol levels in The Netherlands: identification and characterization of eight novel mutations. *Human mutation* **32**: 1290-1298.	Clinical and Laboratory data; Characterization of recombinant variants	Family	FLD
80	Holmquist, L., and L. A. Carlson. (1987) Alpha-lecithin:cholesterol acyltransferase deficiency. Lack of both phospholipase A2 and acyltransferase activities characteristic of high density lipoprotein lecithin:cholesterol acyltransferase in fish eye disease. *Acta Med Scand* **222**: 23-26.	Laboratory data	Case collection	FED
81	Homma, S., N. Murayama, I. Yoshida, E. Kusano, K. Kuriki, K. Saito, and Y. Asano. (2001) Marked atherosclerosis in a patient with familiar lecithin: cholesterol acyltransferase deficiency associated with end-stage renal disease and diabetes mellitus. *American journal of nephrology* **21**: 415-419.	Clinical and Laboratory data	Case	FLD
82	Homma, S., N. Murayama, T. Kodama, N. Yamada, K. Takahashi, Y. Asano, S. Hosoda, T. Murase, and Y. Akanuma. (1993) Effects of long-term, low-fat diet on plasma apo E in familial LCAT deficiency. *The Japanese Journal of Nephrology* **35**: 999-1006.	Clinical and Laboratory data	Case	FLD
83	Horina, J. H., G. Wirnsberger, S. Horn, J. M. Roob, M. Ratschek, H. Holzer, H. Pogglitsch, and G. J. Krejs. (1993) Long-term follow-up of a patient with lecithin cholesterol acyltransferase deficiency syndrome after kidney transplantation. *Transplantation* **56**: 233-236.	Clinical data	Case	FLD
84	Horl, G., P. M. Kroisel, E. Wagner, B. Tiran, E. Petek, and E. Steyrer. (2006) Compound heterozygosity (G71R/R140H) in the lecithin:cholesterol acyltransferase (LCAT) gene results in an intermediate phenotype between LCAT-deficiency and fish-eye disease. *Atherosclerosis* **187**: 101-109.	Clinical and Laboratory data; Characterization of recombinant variants	Family	FLD
85	Horven, I., K. Egge, and E. Gjone. (1974) Corneal and fundus changes in familial LCAT-deficiency. *Acta Ophthalmol (Copenh)* **52**: 201-210.	Clinical data	Case collection	FLD
86	Hovig, T., and E. Gjone. (1973) Familial plasma lecithin: cholesterol acyltransferase (LCAT) deficiency. Ultrastructural aspects of a new syndrome with particular reference to lesions in the kidneys and the spleen. *Acta pathologica et microbiologica Scandinavica. Section A, Pathology* **81**: 681-697.	Clinical data	Case collection	FLD
87	Hovig, T., and E. Gjone. (1973) Ultrastructural aspects of familial lecithin-cholesterol acyltransferase deficiency. *Nutr Metab* **15**: 89-96.	Clinical data	Case	FLD
88	Hovig, T., and E. Gjone. (1974) Familial lecithin:cholesterol acyltransferase deficiency. Ultrastructural studies on lipid deposition and tissue reactions. *Scand J Clin Lab Invest Suppl* **137**: 135-146.	Clinical data	Case	FLD

89	Hovingh, G. K., B. A. Hutten, A. G. Holleboom, W. Petersen, P. Rol, A. Stalenhoef, A. H. Zwinderman, E. de Groot, J. J. Kastelein, and J. A. Kuivenhoven. (2005) Compromised LCAT function is associated with increased atherosclerosis. *Circulation* **112**: 879-884.	Clinical and Laboratory data	Case collection	FED
90	Ibrahim, O., L. Amah, and S. E. Mace. (2015) Corneal opacities in a man with chronic kidney disease. *Cleveland Clinic journal of medicine* **82**: 339-340.	Clinical and Laboratory data	Case	FLD
91	Idzior-Walus, B., J. Sieradzki, G. Kostner, M. T. Malecki, T. Klupa, T. Wesolowska, W. Rostworowski, J. Hartwich, M. Walus, A. D. Kiec, and M. Naruszewicz. (2006) Familial lecithin-cholesterol acyltransferase deficiency: biochemical characteristics and molecular analysis of a new LCAT mutation in a Polish family. *Atherosclerosis* **185**: 413-420.	Clinical and Laboratory data	Family	FLD
92	Imbasciati, E., C. Paties, L. Scarpioni, and M. J. Mihatsch. (1986) Renal lesions in familial lecithin-cholesterol acyltransferase deficiency. Ultrastructural heterogeneity of glomerular changes. *American journal of nephrology* **6**: 66-70.	Clinical and Laboratory data	Case	FLD
93	Ingle, V. K., P. K. Maharana, and P. Rajesh. (2016) Secret in the eyes - fish eye disease. *Indian journal of nephrology* **26**: 313-314.	Clinical and Laboratory data	Case	FED
94	Iwamoto, A., C. Naito, T. Teramoto, H. Kato, M. Kako, T. Kariya, T. Shimizu, H. Oka, and T. Oda. (1978) Familial lecithin: cholesterol acyltransferase deficiency complicated with unconjugated hyperbilirubinemia and peripheral neuropathy. The first reported cases in the Far East. *Acta Med Scand* **204**: 219-227.	Clinical and Laboratory data	Family	FLD
95	Jahanzad, I., S. Amoueian, and A. Attaranzadeh. (2009) Familial lecithin-cholesterol acyltransferase deficiency. *Archives of Iranian medicine* **12**: 179-181.	Clinical and Laboratory data	Case	FLD
96	Jain, S. K., N. Mohandas, G. F. Sensabaugh, A. M. Shojania, and S. B. Shohet. (1982) Hereditary plasma lecithin-cholesterol acyl transferase deficiency: a heterozygous variant with erythrocyte membrane abnormalities. *The Journal of laboratory and clinical medicine* **99**: 816-826.	Clinical and Laboratory data	Family	FLD
97	Jimi, S., N. Uesugi, K. Saku, H. Itabe, B. Zhang, K. Arakawa, and S. Takebayashi. (1999) Possible induction of renal dysfunction in patients with lecithin:cholesterol acyltransferase deficiency by oxidized phosphatidylcholine in glomeruli. *Arterioscler Thromb Vasc Biol* **19**: 794-801.	Clinical and Laboratory data	Case collection	FLD; Unclassified
98	Kanai, M., S. Koh, D. Masuda, M. Koseki, and K. Nishida. (2018) Clinical features and visual function in a patient with Fish-eye disease: Quantitative measurements and optical coherence tomography. *American journal of ophthalmology case reports* **10**: 137-141.	Clinical and Laboratory data	Case	FED
99	Kasid, A., J. Rhyne, K. Zeller, H. Pritchard, and M. Miller. (2001) A novel TC deletion resulting in Pro(260)-->Stop in the human LCAT gene is associated with a dominant effect on HDL-cholesterol. *Atherosclerosis* **156**: 127-132.	Clinical and Laboratory data	Family	Non FLD/FED
100	Kastelein, J. J., P. H. Pritchard, D. W. Erkelens, J. A. Kuivenhoven, J. J. Albers, and J. J. Frohlich. (1992) Familial high-density-lipoprotein deficiency causing corneal opacities (fish eye disease) in a family of Dutch descent. *Journal of internal medicine* **231**: 413-419.	Clinical and Laboratory data	Family	FED
101	Katayama, A., J. Wada, H. U. Kataoka, H. Yamasaki, S. Teshigawara, T. Terami, K. Inoue, M. Kanzaki, K. Murakami, A. Nakatsuka, H. Sugiyama, N. Koide, H. Bujo, and H. Makino. (2011) Two novel mutations of lecithin:cholesterol acyltransferase (LCAT) gene and the influence of APOE genotypes on clinical manifestations. *NDT plus* **4**: 299-302.	Clinical and Laboratory data	Case collection	FLD
102	Kettritz, R., S. Elitok, M. L. Koepke, J. Kuchenbecker, W. Schneider, and F. C. Luft. (2009) The case: the eyes have it! *Kidney Int* **76**: 465-466.	Clinical and Laboratory data	Case	FLD
103	Klein, H. G., N. Duverger, J. J. Albers, S. Marcovina, H. B. Brewer, Jr., and S. Santamarina-Fojo. (1995) In vitro expression of structural defects in the lecithin-cholesterol acyltransferase gene. *The Journal of biological chemistry* **270**: 9443-9447.	Characterization of recombinant variants	NA	NA
104	Klein, H. G., P. Lohse, N. Duverger, J. J. Albers, D. J. Rader, L. A. Zech, S. Santamarina-Fojo, and H. B. Brewer, Jr. (1993) Two different allelic mutations in the lecithin:cholesterol acyltransferase (LCAT) gene resulting in classic LCAT deficiency: LCAT (tyr83-->stop) and LCAT (tyr156-->asn). *J Lipid Res* **34**: 49-58.	Clinical and Laboratory data	Family	FLD
105	Klein, H. G., P. Lohse, P. H. Pritchard, D. Bojanovski, H. Schmidt, and H. B. Brewer, Jr. (1992) Two different allelic mutations in the lecithin-cholesterol acyltransferase gene associated with the fish eye syndrome. Lecithin-cholesterol acyltransferase (Thr123----Ile) and lecithin-cholesterol acyltransferase (Thr347----Met). *The Journal of clinical investigation* **89**: 499-506.	Clinical and Laboratory data	Family	FED
106	Klein, H. G., S. Santamarina-Fojo, N. Duverger, M. Clerc, M. F. Dumon, J. J. Albers, S. Marcovina, and H. B. Brewer, Jr. (1993) Fish eye syndrome: a molecular defect in the lecithin-cholesterol acyltransferase (LCAT) gene associated with	Clinical and Laboratory data; Characterization of	Family	FED

	normal alpha-LCAT-specific activity. Implications for classification and prognosis. *The Journal of clinical investigation* **92**: 479-485.	recombinant variants		
107	Koster, H., M. Savoldelli, M. F. Dumon, L. Dubourg, M. Clerc, and Y. Pouliquen. (1992) A fish-eye disease-like familial condition with massive corneal clouding and dyslipoproteinemia. Report of clinical, histologic, electron microscopic, and biochemical features. *Cornea* **11**: 452-464.	Clinical and Laboratory data	Family	FED
108	Kuivenhoven, J. A., A. F. Stalenhoef, J. S. Hill, P. N. Demacker, A. Errami, J. J. Kastelein, and P. H. Pritchard. (1996) Two novel molecular defects in the LCAT gene are associated with fish eye disease. *Arterioscler Thromb Vasc Biol* **16**: 294-303.	Clinical and Laboratory data; Characterization of recombinant variants	Family	FED
109	Kuivenhoven, J. A., E. J. van Voorst tot Voorst, H. Wiebusch, S. M. Marcovina, H. Funke, G. Assmann, P. H. Pritchard, and J. J. Kastelein. (1995) A unique genetic and biochemical presentation of fish-eye disease. *The Journal of clinical investigation* **96**: 2783-2791.	Clinical and Laboratory data	Family	FED
110	Kuivenhoven, J. A., H. Weibusch, P. H. Pritchard, H. Funke, R. Benne, G. Assmann, and J. J. Kastelein. (1996) An intronic mutation in a lariat branchpoint sequence is a direct cause of an inherited human disorder (fish-eye disease). *The Journal of clinical investigation* **98**: 358-364.	Clinical and Laboratory data; Characterization of recombinant variants	Family	FED
111	Kuroda, M., A. G. Holleboom, E. S. Stroes, S. Asada, Y. Aoyagi, K. Kamata, S. Yamashita, S. Ishibashi, Y. Saito, and H. Bujo. (2014) Lipoprotein subfractions highly associated with renal damage in familial lecithin:cholesterol acyltransferase deficiency. *Arterioscler Thromb Vasc Biol* **34**: 1756-1762.	Clinical and Laboratory data	Case collection	FLD; FED
112	Lager, D. J., B. F. Rosenberg, H. Shapiro, and J. Bernstein. (1991) Lecithin cholesterol acyltransferase deficiency: ultrastructural examination of sequential renal biopsies. *Modern pathology : an official journal of the United States and Canadian Academy of Pathology, Inc* **4**: 331-335.	Clinical data	Family	FLD
113	Lamiquiz-Moneo, I., F. Civeira, D. Gomez-Coronado, F. Blanco-Vaca, H. M. Villafuerte-Ledesma, M. Gil, N. Amigo, R. Mateo-Gallego, and A. Cenarro. (2019) Lipid Profile Rather Than the LCAT Mutation Explains Renal Disease in Familial LCAT Deficiency. *Journal of clinical medicine* **8**.	Clinical and Laboratory data	Family	FLD
114	Li, M., and P. H. Pritchard. (2000) Characterization of the effects of mutations in the putative branchpoint sequence of intron 4 on the splicing within the human lecithin:cholesterol acyltransferase gene. *The Journal of biological chemistry* **275**: 18079-18084.	Characterization of recombinant variants	NA	NA
115	Li, M., J. A. Kuivenhoven, A. F. Ayyobi, and P. H. Pritchard. (1998) T-->G or T-->A mutation introduced in the branchpoint consensus sequence of intron 4 of lecithin:cholesterol acyltransferase (LCAT) gene: intron retention causing LCAT deficiency. *Biochimica et biophysica acta* **1391**: 256-264.	Characterization of recombinant variants	NA	NA
116	Liew, H., I. Simpson, J. Kanellis, and W. R. Mulley. (2016) Recurrent glomerulopathy in a renal allograft due to lecithin cholesterol acyltransferase deficiency. *Nephrology (Carlton)* **21**: 73-74.	Clinical data	Case	FLD
117	Lucchi, T., L. Calabresi, A. Pinto, E. Benetti, B. Arosio, S. Simonelli, R. Ratiglia, and C. Vergani. (2012) A woman with low HDL cholesterol and corneal opacity. *Internal and emergency medicine* **7**: 533-537.	Clinical and Laboratory data	Family	FLD
118	Mackness, M. I., C. H. Walker, and L. A. Carlson. (1987) Low A-esterase activity in serum of patients with fish-eye disease. *Clin Chem* **33**: 587-588.	Laboratory data	Case collection	FED
119	Maeda, E., Y. Naka, T. Matozaki, M. Sakuma, Y. Akanuma, G. Yoshino, and M. Kasuga. (1991) Lecithin-cholesterol acyltransferase (LCAT) deficiency with a missense mutation in exon 6 of the LCAT gene. *Biochemical and biophysical research communications* **178**: 460-466.	Clinical and Laboratory data	Case	FED
120	Magil, A., W. Chase, and J. Frohlich. (1982) Unusual renal biopsy findings in a patient with familial lecithin:cholesterol acyltransferase deficiency. *Human pathology* **13**: 283-285.	Clinical and Laboratory data	Case	FLD
121	Mahapatra, H. S., S. Ramanarayanan, A. Gupta, and M. Bhardwaj. (2015) Co-existence of classic familial lecithin-cholesterol acyl transferase deficiency and fish eye disease in the same family. *Indian journal of nephrology* **25**: 362-365.	Clinical and Laboratory data	Case	FLD
122	Manthei, K. A., J. Ahn, A. Glukhova, W. Yuan, C. Larkin, T. D. Manett, L. Chang, J. A. Shayman, M. J. Axley, A. Schwendeman, and J. J. G. Tesmer. (2017) A retractable lid in lecithin:cholesterol acyltransferase provides a structural mechanism for activation by apolipoprotein A-I. *The Journal of biological chemistry* **292**: 20313-20327.	Characterization of recombinant variants	NA	NA

123	McConathy, W. J., P. Alaupovic, M. D. Curry, H. N. Magnani, H. Torsvik, K. Berg, and E. Gjone. (1973) Identification of lipoprotein families in familial lecithin: cholesterol acyltransferase deficiency. *Biochimica et biophysica acta* **326**: 406-418.	Laboratory data	Case collection	FLD
124	Miarka, P., B. Idzior-Walus, M. Kuzniewski, M. Walus-Miarka, T. Klupa, and W. Sulowicz. (2011) Corticosteroid treatment of kidney disease in a patient with familial lecithin-cholesterol acyltransferase deficiency. *Clinical and experimental nephrology* **15**: 424-429.	Clinical and Laboratory data	Family	FLD
125	Miettinen, H. E., H. Gylling, J. Tenhunen, J. Virtamo, M. Jauhiainen, J. K. Huttunen, I. Kantola, T. A. Miettinen, and K. Kontula. (1998) Molecular genetic study of Finns with hypoalphalipoproteinemia and hyperalphalipoproteinemia: a novel Gly230 Arg mutation (LCAT[Fin]) of lecithin:cholesterol acyltransferase (LCAT) accounts for 5% of cases with very low serum HDL cholesterol levels. *Arterioscler Thromb Vasc Biol* **18**: 591-598.	Clinical and Laboratory data; Characterization of recombinant variants	Family	FLD
126	Miettinen, H., H. Gylling, I. Ulmanen, T. A. Miettinen, and K. Kontula. (1995) Two different allelic mutations in a Finnish family with lecithin:cholesterol acyltransferase deficiency. *Arterioscler Thromb Vasc Biol* **15**: 460-467.	Clinical and Laboratory data; Characterization of recombinant variants	Population	FLD; Unclassified
127	Miida, T., B. Zhang, K. Obayashi, U. Seino, Y. Zhu, T. Ito, Y. Nakamura, M. Okada, and K. Saku. (2004) T13M mutation of lecithin-cholesterol acyltransferase gene causes fish-eye disease. *Clinica chimica acta; international journal of clinical chemistry* **343**: 201-208.	Clinical and Laboratory data; Characterization of recombinant variants	Family	FED
128	Miller, M., K. Zeller, P. C. Kwiterovich, J. J. Albers, and G. Feulner. (1995) Lecithin: cholesterol acyltransferase deficiency: identification of two defective alleles in fibroblast cDNA. *J Lipid Res* **36**: 931-938.	Clinical and Laboratory data; Characterization of recombinant variants	Family	FLD
129	Miyatake, T., Y. Matsui, M. Koyama, T. Watanabe, S. Yamada, and K. Yasuda. (2005) Cardiac surgery for a patient with familial lecithin: cholesterol acyltransferase deficiency. *The Japanese journal of thoracic and cardiovascular surgery : official publication of the Japanese Association for Thoracic Surgery = Nihon Kyobu Geka Gakkai zasshi* **53**: 317-319.	Clinical and Laboratory data	Case	FED
130	Morales, E., M. Alonso, B. Sarmiento, and M. Morales. (2018) LCAT deficiency as a cause of proteinuria and corneal opacification. *BMJ case reports* **2018**.	Clinical and Laboratory data	Case	FLD
131	Moriyama, K., J. Sasaki, F. Arakawa, N. Takami, E. Maeda, A. Matsunaga, Y. Takada, K. Midorikawa, T. Yanase, G. Yoshino, and et al. (1995) Two novel point mutations in the lecithin:cholesterol acyltransferase (LCAT) gene resulting in LCAT deficiency: LCAT (G873 deletion) and LCAT (Gly344-->Ser). *J Lipid Res* **36**: 2329-2343.	Clinical and Laboratory data; Characterization of recombinant variants	Family	FLD
132	Murano, S., K. Shirai, Y. Saito, S. Yoshida, Y. Ohta, H. Tsuchida, S. Yamamoto, G. Asano, C. H. Chen, and J. J. Albers. (1987) Impaired intermediate-density lipoprotein triglyceride hydrolysis in familial lecithin:cholesterol acyltransferase (LCAT) deficiency. *Scandinavian journal of clinical and laboratory investigation* **47**: 775-783.	Clinical and Laboratory data	Case	FLD
133	Murayama, N., Y. Asano, K. Kato, Y. Sakamoto, S. Hosoda, N. Yamada, T. Kodama, T. Murase, and Y. Akanuma. (1984) Effects of plasma infusion on plasma lipids, apoproteins and plasma enzyme activities in familial lecithin: cholesterol acyltransferase deficiency. *Eur J Clin Invest* **14**: 122-129.	Laboratory data	Case	FLD
134	Murayama, N., Y. Asano, S. Hosoda, M. Maesawa, M. Saito, F. Takaku, T. Sugihara, K. Miyashima, and Y. Yawata. (1984) Decreased sodium influx and abnormal red cell membrane lipids in a patient with familial plasma lecithin: cholesterol acyltransferase deficiency. *American journal of hematology* **16**: 129-137.	Clinical and Laboratory data	Family	FLD
135	Muthusethupathi, M. A., R. Padmanabhan, A. Date, M. Jayakumar, S. Rajendran, and R. Vijayakumar. (1999) Familial Lecithin:cholesterol acyltransferase deficiency with renal failure in two siblings. First case report from India. *Nephron* **81**: 89-93.	Clinical and Laboratory data	Family	FLD
136	Myhre, E., E. Gjone, A. Flatmark, and T. Hovig. (1977) Renal failure in familial lecithin-cholesterol acyltransferase deficiency. *Nephron* **18**: 239-248.	Clinical and Laboratory data	Case collection	FLD

137	Naghashpour, M., and H. Cualing. (2009) Splenomegaly with sea-blue histiocytosis, dyslipidemia, and nephropathy in a patient with lecithin-cholesterol acyltransferase deficiency: a clinicopathologic correlation. *Metabolism: clinical and experimental* **58**: 1459-1464.	Clinical and Laboratory data	Case	FLD
138	Naito, C., T. Teramoto, H. Kato, T. Watanabe, T. Yamanaka, and A. Iwamoto. (1978) Lipid compositions of plasma major lipoproteins and lipoprotein lipase activity in hypolipidemic and hyperlipidemic siblings with familial LCAT deficiency. *Scand J Clin Lab Invest Suppl* **150**: 168-176.	Laboratory data	Family	FLD
139	Naito, M., E. Maeda, G. Yoshino, M. Kasuga, A. Iguchi, and F. Kuzuya. (1994) Japanese family with a deficiency of lecithin:cholesterol acyltransferase (LCAT). *Internal medicine (Tokyo, Japan)* **33**: 677-682.	Clinical and Laboratory data	Family	FLD
140	Naito, S., M. Kamata, M. Furuya, M. Hayashi, M. Kuroda, H. Bujo, and K. Kamata. (2013) Amelioration of circulating lipoprotein profile and proteinuria in a patient with LCAT deficiency due to a novel mutation (Cys74Tyr) in the lid region of LCAT under a fat-restricted diet and ARB treatment. *Atherosclerosis* **228**: 193-197.	Clinical and Laboratory data	Case	FLD
141	Nanjee, M. N., J. Stocks, C. J. Cooke, H. O. Molhuizen, S. Marcovina, D. Crook, J. P. Kastelein, and N. E. Miller. (2003) A novel LCAT mutation (Phe382-->Val) in a kindred with familial LCAT deficiency and defective apolipoprotein B-100. *Atherosclerosis* **170**: 105-113.	Clinical and Laboratory data	Family	Unclassified
142	Neumann, M. J., and S. Whisiker-Lewis. (1994) Lecithin-cholesterol acyltransferase deficiency: first report of case in a United States citizen. *The Journal of the American Osteopathic Association* **94**: 333-335.	Clinical and Laboratory data	Case	FLD
143	Nishiwaki, M., K. Ikewaki, G. Bader, H. Nazih, M. Hannuksela, A. T. Remaley, R. D. Shamburek, and H. B. Brewer, Jr. (2006) Human lecithin:cholesterol acyltransferase deficiency: in vivo kinetics of low-density lipoprotein and lipoprotein-X. *Arterioscler Thromb Vasc Biol* **26**: 1370-1375.	Clinical and Laboratory data	Case collection	FLD
144	Norum, K. R., and E. Gjone. (1967) Familial Plasma Lecithin: Cholesterol Acyltransferase Deficiency Biochemical Study of a New Inborn Error of Metabolism. *Scandinavian Journal of Clinical and Laboratory Investigation* **20**: 231-243.	Clinical and Laboratory data	Family	FLD
145	Norum, K. R., and E. Gjone. (1967) Familial serum-cholesterol esterification failure. A new inborn error of metabolism. *Biochimica et biophysica acta* **144**: 698-700.	Laboratory data	Family	FLD
146	Norum, K. R., and E. Gjone. (1968) The effect of plasma transfusion on the plasma cholesterol esters in patients with familial plasma lecithin: cholesterol acyltransferase deficiency. *Scand J Clin Lab Invest* **22**: 339-342.	Clinical and Laboratory data	Family	FLD
147	Norum, K. R., J. A. Glomset, A. V. Nichols, and T. Forte. (1971) Plasma lipoproteins in familial lecithin: cholesterol acyltransferase deficiency: physical and chemical studies of low and high density lipoproteins. *The Journal of clinical investigation* **50**: 1131-1140.	Laboratory data	Family	FLD
148	Norum, K. R., S. Borsting, and I. Grundt. (1970) Familial lecithin: cholesterol acyltransferase deficiency. Study of two new patients and their close relatives. *Acta Med Scand* **188**: 323-326.	Laboratory data	Case collection	FLD
149	O, K., J. S. Hill, X. Wang, and P. H. Pritchard. (1993) Recombinant lecithin:cholesterol acyltransferase containing a Thr123-->Ile mutation esterifies cholesterol in low density lipoprotein but not in high density lipoprotein. *J Lipid Res* **34**: 81-88.	Characterization of recombinant variants	NA	NA
150	Ohta, T., S. Hattori, R. Nakamura, S. Horiuchi, J. Frohlich, K. Takata, Y. Ikeda, Y. Saito, and I. Matsuda. (1994) Characterization of subspecies of apolipoprotein A-I-containing lipoprotein in homozygotes for familial lecithin:cholesterol acyltransferase deficiency. *Arteriosclerosis and thrombosis : a journal of vascular biology* **14**: 1137-1145.	Laboratory data	Case collection	FLD
151	Ohta, Y., S. Yamamoto, H. Tsuchida, S. Murano, Y. Saitoh, S. Tohjo, and M. Okada. (1986) Nephropathy of familial lecithin-cholesterol acyltransferase deficiency: report of a case. *American journal of kidney diseases : the official journal of the National Kidney Foundation* **7**: 41-46.	Clinical and Laboratory data	Family	FLD
152	Okubo, M., Y. Aoyama, H. Shio, J. J. Albers, and T. Murase. (1996) A novel missense mutation (Asn5-->Ile) in lecithin: cholesterol acyltransferase (LCAT) gene in a Japanese patient with LCAT deficiency. *Int J Clin Lab Res* **26**: 250-254.	Clinical and Laboratory data	Case	FLD
153	Oliaei, F., B. Batebi, R. Tabaripour, and H. Akhavan Niaki. (2018) Finding a very rare mutation in non-Caucasian LCAT patients from Southwest Asia for the first time. *Journal of cellular biochemistry*.	Clinical and Laboratory data	Case collection	FLD
154	Owen, J. S., H. Wiebusch, P. Cullen, G. F. Watts, V. L. Lima, H. Funke, and G. Assmann. (1996) Complete deficiency of plasma lecithin-cholesterol acyltransferase (LCAT) activity due to a novel homozygous mutation (Gly-30-Ser) in the LCAT gene. *Human mutation* **8**: 79-82.	Clinical and Laboratory data	Family	FLD
155	Ozkok, A., Y. Onal, I. Kilicaslan, S. Solakoglu, H. Bayramlar, and A. R. Odabas. (2017) The Case | Nephrotic syndrome with corneal opacities. *Kidney Int* **91**: 515-516.	Clinical and Laboratory data	Case	FLD

156	Palmiero, P. M., Z. Sbeity, J. Liebmann, and R. Ritch. (2009) In vivo imaging of the cornea in a patient with lecithin-cholesterol acyltransferase deficiency. *Cornea* **28**: 1061-1064.	Clinical data	Case	FED
157	Panescu, V., Y. Grignon, D. Hestin, G. Rostoker, L. Frimat, E. Renoult, J. Gamberoni, G. Grignon, and M. Kessler. (1997) Recurrence of lecithin cholesterol acyltransferase deficiency after kidney transplantation. *Nephrology, dialysis, transplantation : official publication of the European Dialysis and Transplant Association - European Renal Association* **12**: 2430-2432.	Clinical and Laboratory data	Case	FLD
158	Park, C. W., M. H. Lim, D. Y. Youn, S. E. Jung, S. Chung, Y. S. Ahn, Y. S. Chang, and J. H. Lee. (2009) Two novel frame shift mutations in lecithin:cholesterol acyltransferase (LCAT) gene associated with a familial LCAT deficiency phenotype. *Atherosclerosis* **206**: 346-348.	Clinical and Laboratory data; Characterization of recombinant variants	Family	FLD
159	Peelman, F., J. L. Verschelde, B. Vanloo, C. Ampe, C. Labeur, J. Tavernier, J. Vandekerckhove, and M. Rosseneu. (1999) Effects of natural mutations in lecithin:cholesterol acyltransferase on the enzyme structure and activity. *J Lipid Res* **40**: 59-69.	Characterization of recombinant variants	NA	NA
160	Pisciotta, L., L. Calabresi, G. Lupattelli, D. Siepi, M. R. Mannarino, E. Moleri, A. Bellocchio, A. Cantafora, P. Tarugi, S. Calandra, and S. Bertolini. (2005) Combined monogenic hypercholesterolemia and hypoalphalipoproteinemia caused by mutations in LDL-R and LCAT genes. *Atherosclerosis* **182**: 153-159.	Clinical and Laboratory data	Family	Unclassified
161	Posadas-Sánchez, R., C. Posadas-Romero, W. A. Ocampo-Arcos, M. T. Villarreal-Molina, G. Vargas-Alarcón, E. Antúnez-Argüelles, E. Mendoza-Pérez, G. Cardoso-Saldaña, R. Martínez-Alvarado, A. Medina-Urrutia, and E. Jorge-Galarza. (2014) Premature and severe cardiovascular disease in a Mexican male with markedly low high-density-lipoprotein-cholesterol levels and a mutation in the lecithin:cholesterol acyltransferase gene: a family study. *Int J Mol Med* **33**: 1570-1576.	Clinical and Laboratory data	Family	FED
162	Qu, S. J., H. Z. Fan, F. Blanco-Vaca, and H. J. Pownall. (1995) In vitro expression of natural mutants of human lecithin:cholesterol acyltransferase. *J Lipid Res* **36**: 967-974.	Characterization of recombinant variants	NA	NA
163	Rader, D. J., K. Ikewaki, N. Duverger, H. Schmidt, H. Pritchard, J. Frohlich, M. Clerc, M. F. Dumon, T. Fairwell, L. Zech, and et al. (1994) Markedly accelerated catabolism of apolipoprotein A-II (ApoA-II) and high density lipoproteins containing ApoA-II in classic lecithin: cholesterol acyltransferase deficiency and fish-eye disease. *The Journal of clinical investigation* **93**: 321-330.	Clinical and Laboratory data	Case collection	FLD; FED
164	Rajpal, J. S., J. Mapel-Lentz, A. D. Mancera, R. C. Reed, Y. Kim, and B. M. Chavers. (2014) Familial LCAT deficiency in a child with nephrotic syndrome. *Clinical nephrology* **82**: 211-214.	Clinical and Laboratory data	Case	FLD
165	Rial-Crestelo, D., I. Santos-Recuero, J. Julve, F. Blanco-Vaca, and M. Torralba. (2017) A novel homozygous mutation causing lecithin-cholesterol acyltransferase deficiency in a proband of Romanian origin with a record of extreme gestational hyperlipidemia. *J Clin Lipidol* **11**: 1475-1479.e1473.	Clinical and Laboratory data	Family	FLD
166	Ritland, S., and E. Gjone. (1975) Quantitative studies of lipoprotein-X in familial lecithin: cholesterol acyltransferase deficiency and during cholesterol esterification. *Clinica chimica acta; international journal of clinical chemistry* **59**: 109-119.	Laboratory data	Case collection	FLD
167	Roshan, B., O. P. Ganda, R. Desilva, R. B. Ganim, E. Ward, S. D. Haessler, E. Y. Polisecki, B. F. Asztalos, and E. J. Schaefer. (2011) Homozygous lecithin:cholesterol acyltransferase (LCAT) deficiency due to a new loss of function mutation and review of the literature. *J Clin Lipidol* **5**: 493-499.	Clinical and Laboratory data	Case	FLD
168	Rosset, J., J. Wang, B. M. Wolfe, P. J. Dolphin, and R. A. Hegele. (2001) Lecithin:cholesterol acyl transferase G30S: association with atherosclerosis, hypoalphalipoproteinemia and reduced in vivo enzyme activity. *Clinical biochemistry* **34**: 381-386.	Clinical and Laboratory data	Case	FLD-Het
169	Sahay, M., P. S. Vali, K. Ismal, S. Gowrishankar, M. D. Padua, and M. Swain. (2016) An unusual case of nephrotic syndrome. *Indian journal of nephrology* **26**: 55-56.	Clinical and Laboratory data	Case	FLD
170	Sakuma, M., Y. Akanuma, T. Kodama, N. Yamada, S. Murata, T. Murase, H. Itakura, and K. Kosaka. (1982) Familial plasma lecithin: cholesterol acyltransferase deficiency. A new family with partial LCAT activity. *Acta Med Scand* **212**: 225-232.	Clinical and Laboratory data	Family	FED
171	Savel, J., M. Lafitte, Y. Pucheu, V. Pradeau, A. Tabarin, and T. Couffinhal. (2012) Very low levels of HDL cholesterol and atherosclerosis, a variable relationship--a review of LCAT deficiency. *Vasc Health Risk Manag* **8**: 357-361.	Clinical and Laboratory data	Case	FED

172	Scarpioni, R., C. Paties, and G. Bergonzi. (2008) Dramatic atherosclerotic vascular burden in a patient with familial lecithin-cholesterol acyltransferase (LCAT) deficiency. *Nephrology, dialysis, transplantation : official publication of the European Dialysis and Transplant Association - European Renal Association* **23**: 1074;-author reply 1074-1075.	Clinical and Laboratory data	Case	FLD
173	Sessa, A., G. Battini, M. Meroni, G. Daidone, I. Carnera, P. L. Brambilla, G. Vigano, F. Giordano, F. Pallotti, L. Torri Tarelli, L. Calabresi, M. Rolleri, and S. Bertolini. (2001) Hypocomplementemic type II membranoproliferative glomerulonephritis in a male patient with familial lecithin-cholesterol acyltransferase deficiency due to two different allelic mutations. *Nephron* **88**: 268-272.	Clinical and Laboratory data	Family	FLD
174	Shamburek, R. D., R. Bakker-Arkema, B. J. Auerbach, B. R. Krause, R. Homan, M. J. Amar, L. A. Freeman, and A. T. Remaley. (2016) Familial lecithin:cholesterol acyltransferase deficiency: First-in-human treatment with enzyme replacement. *J Clin Lipidol* **10**: 356-367.	Clinical and Laboratory data	Case	FLD
175	Shojania, A. M., P. J. McAlpine, and M. Ray. (1983) Hereditary lecithin-cholesterol acyltransferase deficiency and Bloom syndrome in the same individual. *American journal of medical genetics* **14**: 479-485.	Clinical and Laboratory data	Family	FLD
176	Shoji, K., H. Morita, Y. Ishigaki, C. J. Rivard, M. Takayasu, K. Nakayama, T. Nakayama, Y. Inoue, M. Ayaki, and A. Yoshimura. (2011) Lecithin-cholesterol acyltransferase (LCAT) deficiency without mutations in the coding sequence: a case report and literature review. *Clinical nephrology* **76**: 323-328.	Clinical and Laboratory data	Case	Unclassified
177	Skretting, G., and H. Prydz. (1992) An amino acid exchange in exon I of the human lecithin: cholesterol acyltransferase (LCAT) gene is associated with fish eye disease. *Biochemical and biophysical research communications* **182**: 583-587.	Genetic data	Case collection	FLD
178	Skretting, G., J. P. Blomhoff, J. Solheim, and H. Prydz. (1992) The genetic defect of the original Norwegian lecithin:cholesterol acyltransferase deficiency families. *FEBS letters* **309**: 307-310.	Genetic data	Case collection	FED
179	Soutar, A. K., B. L. Knight, and N. B. Myant. (1982) The characterization of lipoproteins in the high density fraction obtained from patients with familial lecithin:cholesterol acyltransferase deficiency and their interaction with cultured human fibroblasts. *J Lipid Res* **23**: 380-390.	Clinical and Laboratory data	Family	FLD
180	Sparks, D. L., J. Frohlich, and P. H. Pritchard. (1988) Cholesteryl ester transfer activity in plasma of patients with familial high-density lipoprotein deficiency. *Clin Chem* **34**: 1812-1815.	Laboratory data	Case collection	FLD; FED
181	Steyrer, E., S. Durovic, S. Frank, W. Giessauf, A. Burger, H. Dieplinger, R. Zechner, and G. M. Kostner. (1994) The role of lecithin: cholesterol acyltransferase for lipoprotein (a) assembly. Structural integrity of low density lipoproteins is a prerequisite for Lp(a) formation in human plasma. *The Journal of clinical investigation* **94**: 2330-2340.	Laboratory data; Characterization of recombinant variants	Family	FLD
182	Steyrer, E., S. Haubenwallner, G. Horl, W. Giessauf, G. M. Kostner, and R. Zechner. (1995) A single G to A nucleotide transition in exon IV of the lecithin: cholesterol acyltransferase (LCAT) gene results in an Arg140 to His substitution and causes LCAT-deficiency. *Hum Genet* **96**: 105-109.	Clinical and Laboratory data	Family	FLD
183	Stocks, J., C. J. Cooke, and N. E. Miller. (2000) A common lecithin: cholesterol acyltransferase gene variant (Ser208-->Thr). *Atherosclerosis* **149**: 219-220.	Laboratory data	Population	Non FLD/FED
184	Stoekenbroek, R. M., M. A. van den Bergh Weerman, G. K. Hovingh, B. J. Potter van Loon, C. E. Siegert, and A. G. Holleboom. (2013) Familial LCAT deficiency: from renal replacement to enzyme replacement. *The Netherlands journal of medicine* **71**: 29-31.	Clinical and Laboratory data	Family	FLD
185	Stokke, K. T., K. S. Bjerve, J. P. Blomhoff, B. Oystese, A. Flatmark, K. R. Norum, and E. Gjone. (1974) Familial lecithin:cholesterol acyltransferase deficiency. Studies on lipid composition and morphology of tissues. *Scand J Clin Lab Invest Suppl* **137**: 93-100.	Clinical and Laboratory data	Case	FLD
186	Stoynovska, B. B. (1992) Successful renal transplantation in a patient with familial lecithin:cholesterol acyltransferase deficiency. *Transpl Int* **5**: 187.	Clinical and Laboratory data	Case	FLD
187	Strom, E. H., S. Sund, M. Reier-Nilsen, C. Dorje, and T. P. Leren. (2011) Lecithin: Cholesterol Acyltransferase (LCAT) Deficiency: renal lesions with early graft recurrence. *Ultrastruct Pathol* **35**: 139-145.	Clinical and Laboratory data	Family	FLD
188	Suda, T., A. Akamatsu, Y. Nakaya, Y. Masuda, and J. Desaki. (2002) Alterations in erythrocyte membrane lipid and its fragility in a patient with familial lecithin:cholesterol acyltrasferase (LCAT) deficiency. *The journal of medical investigation : JMI* **49**: 147-155.	Clinical and Laboratory data	Case	FLD
189	Takata, K., G. Kajiyama, I. Horiuchi, T. Watanabe, H. Tokumo, and Y. Hirata. (1989) A new case of familial lecithin: cholesterol acyltransferase (LCAT) deficiency--paradoxical findings regarding LCAT mass and activity in 23 members of a family. *Japanese journal of medicine* **28**: 765-771.	Clinical and Laboratory data	Family	Unclassified

190	Taramelli, R., M. Pontoglio, G. Candiani, S. Ottolenghi, H. Dieplinger, A. Catapano, J. Albers, C. Vergani, and J. McLean. (1990) Lecithin cholesterol acyl transferase deficiency: molecular analysis of a mutated allele. *Hum Genet* **85**: 195-199.	Genetic data; Characterization of recombinant variants	Case	FLD
191	Tateno, H., M. Taomoto, N. Jo, K. Takahashi, and Y. Uemura. (2012) Clinical and histopathological features of a suspected case of fish-eye disease. *Japanese journal of ophthalmology* **56**: 453-457.	Clinical and Laboratory data	Case	FED
192	Teh, E. M., J. W. Chisholm, P. J. Dolphin, Y. Pouliquen, M. Savoldelli, J. L. de Gennes, and P. Benlian. (1999) Classical LCAT deficiency resulting from a novel homozygous dinucleotide deletion in exon 4 of the human lecithin: cholesterol acyltransferase gene causing a frameshift and stop codon at residue 144. *Atherosclerosis* **146**: 141-151.	Clinical and Laboratory data	Case	FED
193	Teramoto, T., H. Kato, Y. Hashimoto, M. Kinoshita, H. Oka, and C. Naito. (1986) The basic apolipoprotein A-I in the patients with familial lecithin:cholesterol acyltransferase deficiency. *Scand J Clin Lab Invest* **46**: 297-305.	Laboratory data	Family	FLD
194	Tobar, H. E., L. R. Cataldo, T. Gonzalez, R. Rodriguez, V. Serrano, A. Arteaga, A. Alvarez-Mercado, C. F. Lagos, L. Vicuna, J. P. Miranda, A. Pereira, C. Bravo, C. M. Aguilera, S. Eyheramendy, R. Uauy, et al. (2019) Identification and functional analysis of missense mutations in the lecithin cholesterol acyltransferase gene in a Chilean patient with hypoalphalipoproteinemia. *Lipids in health and disease* **18**: 132.	Clinical and Laboratory data; Characterization of recombinant variants	Case	FED
195	Torsvik, H., E. Gjone, and K. R. Norum. (1968) Familial plasma cholesterol ester deficiency. Clinical studies of a family. *Acta Med Scand* **183**: 387-391.	Clinical and Laboratory data	Family	FLD
196	Tsuchiya, Y., Y. Ubara, R. Hiramatsu, T. Suwabe, J. Hoshino, K. Sumida, E. Hasegawa, M. Yamanouchi, N. Hayami, Y. Marui, N. Sawa, S. Hara, K. Takaichi, and K. Oohashi. (2011) A case of familial lecithin-cholesterol acyltransferase deficiency on hemodialysis for over 20 years. *Clinical nephrology* **76**: 492-498.	Clinical and Laboratory data	Family	FLD
197	Turner, P. R., L. A. Carlson, C. Cortese, S. Rao, C. B. Marenah, N. E. Miller, and B. Lewis. (1984) Studies of lipoprotein metabolism in a patient with fish-eye disease. *Eur J Clin Invest* **14**: 273-277.	Laboratory data	Case	FED
198	Ustaoglu, M., N. Solmaz, B. Baser, H. K. Kurtulgan, and F. Onder. (2019) Ocular and Genetic Characteristics Observed in Two Cases of Fish-Eye Disease. *Cornea* **38**: 379-383.	Clinical and Laboratory data	Family	FED
199	Utermann, G., H. J. Menzel, P. Dieker, K. H. Langer, and G. Fiorelli. (1981) Lecithin-cholesterol-acyltransferase deficiency: autosomal recessive transmission in a large kindred. *Clinical genetics* **19**: 448-455.	Clinical and Laboratory data	Family	FLD
200	Utermann, G., W. Schoenborn, K. H. Langer, and P. Dieker. (1972) Lipoproteins in LCAT-deficiency. *Humangenetik* **16**: 295-306.	Clinical and Laboratory data	Family	FLD
201	Vanloo, B., F. Peelman, K. Deschuymere, J. Taveirne, A. Verhee, C. Gouyette, C. Labeur, J. Vandekerckhove, J. Tavernier, and M. Rosseneu. (2000) Relationship between structure and biochemical phenotype of lecithin:cholesterol acyltransferase (LCAT) mutants causing fish-eye disease. *J Lipid Res* **41**: 752-761.	Characterization of recombinant variants	NA	NA
202	Vergani, C., A. L. Catapano, P. Roma, and G. Giudici. (1983) A new case of familial LCAT deficiency. *Acta Med Scand* **214**: 173-176.	Clinical and Laboratory data	Case	FLD
203	Viestenz, A., U. Schlotzer-Schrehardt, C. Hofmann-Rummelt, B. Seitz, and M. Kuchle. (2002) Histopathology of corneal changes in lecithin-cholesterol acyltransferase deficiency. *Cornea* **21**: 834-837.	Clinical and Laboratory data	Case	FED
204	von Eckardstein, A., Y. Huang, S. Wu, H. Funke, G. Noseda, and G. Assmann. (1995) Reverse cholesterol transport in plasma of patients with different forms of familial HDL deficiency. *Arterioscler Thromb Vasc Biol* **15**: 691-703.	Laboratory data	Case collection	FLD; FED
205	Vrabec, M. P., M. B. Shapiro, E. Koller, D. A. Wiebe, J. Henricks, and J. J. Albers. (1988) Ophthalmic observations in lecithin cholesterol acyltransferase deficiency. *Arch Ophthalmol* **106**: 225-229.	Clinical and Laboratory data	Family	FLD
206	Wang, X. L., J. Osuga, F. Tazoe, K. Okada, S. Nagashima, M. Takahashi, T. Ohshiro, T. Bayasgalan, H. Yagyu, and S. Ishibashi. (2011) Molecular analysis of a novel LCAT mutation (Gly179 --> Arg) found in a patient with complete LCAT deficiency. *Journal of atherosclerosis and thrombosis* **18**: 713-719.	Clinical and Laboratory data; Characterization of recombinant variants	Case	FLD
207	Watts, G. F., K. A. Mitropoulos, A. al-Bahrani, B. E. Reeves, and J. S. Owen. (1995) Lecithin-cholesterol acyltransferase deficiency presenting with acute pancreatitis: effect of infusion of normal plasma on triglyceride-rich lipoproteins. *Journal of internal medicine* **238**: 137-141.	Clinical and Laboratory data	Case	FLD

208	Weber, C. L., J. Frohlich, J. Wang, R. A. Hegele, and C. Chan-Yan. (2007) Stability of lipids on peritoneal dialysis in a patient with familial LCAT deficiency. *Nephrology, dialysis, transplantation : official publication of the European Dialysis and Transplant Association - European Renal Association* **22**: 2084-2088.	Clinical and Laboratory data	Case	FLD
209	Weber, P., J. S. Owen, K. Desai, and M. R. Clemens. (1987) Hereditary lecithin-cholesterol acyltransferase deficiency. Case report of a German patient. *Am J Clin Pathol* **88**: 510-516.	Clinical and Laboratory data	Family	FLD
210	Weidle, E. G., and W. Lisch. (1988) Recognizing familial lecithin cholesterol acyltransferase deficiency at the slit lamp. *Arch Ophthalmol* **106**: 1164.	Clinical and Laboratory data	Case	FLD
211	Wiebusch, H., P. Cullen, J. S. Owen, D. Collins, P. S. Sharp, H. Funke, and G. Assmann. (1995) Deficiency of lecithin:cholesterol acyltransferase due to compound heterozygosity of two novel mutations (Gly33Arg and 30 bp ins) in the LCAT gene. *Hum Mol Genet* **4**: 143-145.	Clinical and Laboratory data	Case	FLD
212	Winder, A. F., A. Garner, G. A. Sheraidah, and P. Barry. (1985) Familial lecithin:cholesterol acyltransferase deficiency. Biochemistry of the cornea. *J Lipid Res* **26**: 283-287.	Clinical and Laboratory data	Case	FLD
213	Winder, A. F., and A. J. Bron. (1978) Lecithin:cholesterol acyl transferase deficiency presenting as visual impairment, with hypocholesterolaemia and normal renal function. *Scand J Clin Lab Invest Suppl* **150**: 151-155.	Clinical data	Case	FLD
214	Winder, A. F., and L. K. Borysiewicz. (1982) Corneal opacification and familial disorders affecting plasma high-density lipoprotein. *Birth defects original article series* **18**: 433-440.	Clinical and Laboratory data	Case	FLD
215	Winder, A. F., J. S. Owen, P. H. Pritchard, D. Lloyd-Jones, D. T. Vallance, P. White, and R. Wray. (1999) A first British case of fish-eye disease presenting at age 75 years: a double heterozygote for defined and new mutations affecting LCAT structure and expression. *Journal of clinical pathology* **52**: 228-230.	Clinical and Laboratory data	Case	Unclassified
216	Yang, X. P., A. Inazu, A. Honjo, I. Koizumi, K. Kajinami, J. Koizumi, S. M. Marcovina, J. J. Albers, and H. Mabuchi. (1997) Catalytically inactive lecithin: cholesterol acyltransferase (LCAT) caused by a Gly 30 to Ser mutation in a family with LCAT deficiency. *J Lipid Res* **38**: 585-591.	Clinical and Laboratory data; Characterization of recombinant variants	Family	FLD
217	Yawata, Y., K. Miyashima, T. Sugihara, N. Murayama, S. Hosoda, S. Nakashima, H. Iida, and Y. Nozawa. (1984) Self-adaptive modification of red-cell membrane lipids in lecithin: cholesterol acyltransferase deficiency. Lipid analysis and spin labeling. *Biochimica et biophysica acta* **769**: 440-448.	Clinical and Laboratory data	Case	FLD
218	Yee, M. S., D. V. Pavitt, W. Richmond, H. T. Cook, A. G. McLean, J. Valabhji, and R. S. Elkeles. (2009) Changes in lipoprotein profile and urinary albumin excretion in familial LCAT deficiency with lipid lowering therapy. *Atherosclerosis* **205**: 528-532.	Clinical and Laboratory data	Case	FLD
219	Zemsky, C. J., S. W. Sherman, H. D. Schubert, and L. H. Suh. (2019) Case Report: Management of Corneal Clouding from Lecithin: Cholesterol Acyltransferase Deficiency. *Optom Vis Sci* **96**: 137-141.	Clinical and Laboratory data	Case	FLD

Table S2. List of variants identified in at least one homozygous subject

Carrier type	Clinical classification	DNA change	Protein change
**1.** HO	FLD	c.31del	p.Val11Ter (1)
**2.** HO	FED	c.86A>G	p.Asn29Ser (2)
**3.** HO	FLD	c.86A>T	p.Asn29Ile (3)
**4.** HO	FED	c.101C>T	p.Pro34Leu (4)
**5.** HO	FLD	c.101insC	p.His35AlafsTer7 (5)
**6.** HO	FLD	c.102del	p.His35ThrfsTer26 (6)
**7.** HO	FED	c.110C>T	p.Thr37Met (7, 8)
**8.** HO	FLD	c.154+1G>C [g.5166G>C]	NA (intronic) (9)
**9.** HO	FLD	c.160G>A	p.Gly54Ser (10-12)
**10.** HO	FED	c.209T>A	p.Val70Glu (1)
**11.** HO	FLD	c.278C>T	p.Pro93Leu (13)
**12.** HO	FLD	c.293G>A	p.Cys98Tyr (10, 14)
**13.** HO	FLD	c.295T>C	p.Trp99Arg (15)
**14.** HO	FLD	c.301G>A	p.Asp101Asn (16)
**15.** HO	FLD	c.321C>A	p.Tyr107Ter (17)
**16.** HO	FLD	c.349G>A* c.544C>T*	p.Ala117Thr* p.Arg182Cys* (17, 18)
**17.** HO	FLD	c.356G>A	p.Gly119Asp (19)
**18.** HO	FED	c.367C>T	p.Arg123Cys (20)
**19.** HO	FLD	c.368G>C	p.Arg123Pro (21)
**20.** HO	FED	c.440C>T	p.Thr147Ile (10, 22, 23)
**21.** HO	FED	c.463A>G	p.Asn155Asp (24)
**22.** HO	FED	c.488_489del	p.Val163AlafsTer6 (25)
**23.** HO	FLD	c.490C>T	p.Arg164Cys (26)
**24.** HO	FLD	c.491G>A	p.Arg164His (27)
**25.** HO	FLD	c.493insGGC	p.Arg164_Ala165insGly (28)
**26.** HO	FLD	c.511C>T	p.Arg171Trp (1, 29, 30)
**27.** HO	FLD	c.512G>A	p.Arg171Gln (31)
**28.** HO	FED	c.544C>T	p.Arg182Cys (32)
**29.** HO	FLD	c.604insA	p.Ile202AsnfsTer27 (33)
**30.** HO	FLD	c.605T>C	p.Ile202Thr (31)
**31.** HO	FLD	c.607G>C	p.Gly203Arg (10, 34)
**32.** HO	NA	c.694T>A	p.Ser232Thr (35)
**33.** HO	FLD	c.698T>C	p.Leu233Pro (17, 36)
**34.** HO	FLD	c.726G>C	p.Lys242Asn (1)
**35.** HO	FLD	c.756C>A	p.Asn252Lys (28)
**36.** HO	FLD	c.760G>C	p.Gly254Arg (37)
**37.** HO	FLD	c.803G>T	p.Arg268Leu (38)
**38.** HO	FLD	c.820 C>T	p.Pro274Ser (39)
**39.** HO	FLD	c.821C>G	p.Pro274Arg (40)
**40.** HO	FLD	c.827T>A	p.Met276Lys (41)
**41.** HO	FLD	c.862del	p.Val288CysfsTer122 (40, 42)
**42.** HO	FLD	c.893C>T	p.Thr298Ile (1)
**43.** HO	FLD	c. 950T>C	p.Met317Thr (13)

Carrier type	Clinical classification	DNA change	Protein change
**44.** HO	FLD	c.950T>G	p.Met317Arg (43-45)
**45.** HO	FED	c.951G>A	p.Met317Ile (28, 46)
**46.** HO	FED	c.970_972del	p.Leu324del (47, 48)
**47.** HO	FLD	c.997G>A	p.Val333Met (49, 50)
**48.** HO	FLD	c.1010G>A	p.Cys337Tyr (10, 51)
**49.** HO	FLD	c.1034C>T	p.Thr345Met (17, 52, 53)
**50.** HO	FED	c.1052 A>G	p.Tyr351Cys (2)
**51.** HO	FLD	c.1102G>A	p.Gly368Ser (40, 42)
**52.** HO	FLD	c.1187T>G	p.Leu396Arg (1, 54)
**53.** HO	Unclassified	c.1267C>T	p.Arg423Cys (37)

Abbreviations: HO, Homozygous subject; FED indicates subjects with Fish-eye disease; FLD indicates subjects with Familial LCAT deficiency; Unclassified indicates subjects who could not be clearly classified as FLD or FED; NA, indicates absence of FLD or FED phenotypes. Variants are described according to the guidelines of Human Genome Variation Society (55) and using (Reference GenBank numbers NG_009778, genomic sequence, NM_000229, coding sequence and their location in the protein sequence, 440 amino acids). Ter indicates termination codon. For frameshift mutations, the first amino acid changed in the mutant protein is indicated. The position of the translation termination codon in the new reading frame is calculated starting at the first amino acid changed. *The variants were identified on the same allele.

Table S3. List of variants only identified in heterozygous and compound heterozygous subjects.

Carrier type	Carrier Clinical classification	Variant	
		DNA change	Protein change
54. CH	FLD (56)	c.54_83dup	p.Leu19_Leu28dup
55. CH	FED (57, 58)	c.96insC	p.His35AlafsTer7
56. CH	FED (59)	c.101C>A	p.Pro34Gln
57. CH	FLD (1)	c.131_155+3del	p.Asn44LysfsTer7
58. CH	FLD (1, 60)	c.141_145del	p.Pro48HisfsTer53
59. CH	FLD (61)	c.154G>A	p.Val52Met
60. CH	FLD (15)	c.167T>C	p.Leu56Pro
61. CH	FLD (56)	c.169G>C	p.Gly57Arg
62. CH	FLD (62)	c.193del	p.Asp65ThrfsTer6
63. CH	FLD (63)	c.283G>C	p.Gly95Arg
64. CH	Unclassified (64)	c.284-285del	p.Val96ArgfsTer6
65. CH	FED (10, 65)	c.296G>C	p.Trp99Ser
66. CH	FED (1)	c.343T>C	p.Ser115Pro
67. CH	FLD (66)	c.388A>G	p.Thr130Ala
68. CH	FED (43)	c.382G>A	p.Gly128Ser
69. CH	FLD (17)	c.475C>T	p.Arg159Trp
70. CH	FED (59)<br>Unclassified(64)	c.476G>A	p.Arg159Gln
71. CH	FED (1)	c.493G>A	p.Ala165Thr
72. CH	FED (67)	c.503A>G	p.Tyr168Cys
73. CH	FED (68)	c.524-22T>C<br>[g.6504T>C]	NA (intronic)
74. CH	FLD (69)	c.538T>A	p.Tyr180Asn
75. CH	FLD (70, 71)	c.576del	p.His192GlnfsTer72
76. CH	FLD (72)	c.585T>G	p.Tyr195Ter
77. CH	FLD (62)	c.602T>A	p.Leu201His
78. CH	FLD (1, 60)	c.614G>A	p.Ser205Asn
79. CH	FLD (73)	c.634insG	p.Leu212AlafsTer16
80. CH	Unclassified (74)	c.694T>A*<br>c.1216T>G*	p.Ser232Thr*<br>Phe406Val*
81. CH	FLD (61)	c.703G>A	p.Ala235Thr
82. CH	FLD(73)	c.794_801del	p.Glu265AlafsTer18
83. CH	FLD (15)	c.802C>T	p.Arg268Cys
84. CH	FLD (75)	c.803G>A	p.Arg268His
85. CH	FLD (1, 76)	c.892A>G	p.Thr298Ala
86. CH	FED (65)	c.945G>A	p.Trp315Ter
87. CH	FED (77)	c.991C>T	p.Pro331Ser
88. CH	FED (65)	c.1012C>T	p.Leu338Phe
89. CH	FED (47, 78)	c.1112C>T	p.Thr371Met
90. CH	FED (65)	c.1039C>T	p.Arg347Cys
91. CH	FLD (17)	c.1198insG	p.Gln400AlafsTer41
92. CH	FED (47)	c.1244A>G	p.Asn415Ser
93. CH	FED (79)	c. 1210A>G	p.Met404Val
94. H	NA (1)	c.35C>T	p.Thr12Met
95. H	NA (43)	c.154+5G>A<br>[g.5170G>A]	p.Arg304His
96. H	NA(43)	c.397delG	p.Val133TrpfsTer131

Carrier type	Carrier Clinical classification	Variant	
		DNA change	Protein change
97. H	NA (65)	c.402G>T*<br>c.403T>A*	p.Glu134Asp*<br>p.Tyr135Asn*
98. H	NA(65)	c.736G>T	p.Val246Phe
99. H	NA (80)	c.835_836del	p.Arg280HisfsTer5
100. H	NA (43)	c.911G>A	p.Arg304His
101. H	NA (65)	c.964C>T	p.Arg322Cys
102. H	NA (43)	c.1123C>T	p.Arg375Cys
103. H	NA (43)	c.1139G>A	p.Cys380Tyr
104. H	Unclassified (81)	c.1289C>T	p. Pro430Leu

Abbreviations: CH, compound heterozygous subject; H, heterozygous subject; FED indicates subjects with Fish-eye disease; FLD indicates subjects with Familial LCAT deficiency; Unclassified indicates subjects who could not be clearly classified as FLD or FED; NA, indicates absence of FLD or FED phenotypes. Variants are described according to the guidelines of Human Genome Variation Society (55) and using (Reference GenBank numbers NG_009778, genomic sequence, NM_000229, coding sequence and their location in the protein sequence, 440 amino acids). Ter indicates termination codon. For frameshift mutations, the first amino acid changed in the mutant protein is indicated. The position of the translation termination codon in the new reading frame is calculated starting at the first amino acid changed. *The variants were identified on the same allele.

Table S4. Unclassified Subjects

Family	Category	Description	Ref
1	Cases with uncertain genotype-phenotype relationship	The proband (woman, age 36 y/o at last determination) presented a phenotype compatible with FLD. She had a clinical history of purpura during childhood and hyperthyroidism that was pharmacologically treated. She presented mild proteinuria since age 17 with normal renal function (age 32). Her lipid profile was consistent with the diagnosis of FLD. Her mother and sister presented with corneal opacity, but normal HDL-C and LCAT activity levels, and no signs of renal disease. Gene analysis in this family did not reveal any genetic abnormality in LCAT exons and the 500 bases upstream (5'). Despite the absence of a defect in the LCAT coding sequence, the presence of clinical signs in different family members suggests the presence of a genetic disease, perhaps in the non-coding region.	(82)
2	Cases with uncertain genotype-phenotype relationship	This subject (male, 75 y/o at last report) was heterozygotes for the c.440C>T (p.Thr147Ile) variant, reported to be FED-causing (table S2 and S10). He had myocardial infarctions at age 49, 73 and 74 y/o. Corneal opacity and reduced HDL-C levels were noted at age 75. He had a history of cutaneous rashes probably caused by psoriasis. His brother, who was deceased at the time of examination died at age 76 and displayed cloudy corneas. HDL-C, ApoA-I levels and LCAT activity on exogenous substrate were dramatically impaired, whereas activity on isolated apoB-containing lipoproteins or whole plasma were normal. A reduction in the total amount of circulating LCAT was also reported. The unusual presentation of this case is unlikely to be explained only by the effect of the c.440C>T (p.Thr147Ile) variant. The presence of a second yet to be identified variant in *LCAT* or in another gene has been suggested.	(83)
3	Cases with uncertain genotype-phenotype relationship	These subjects were 2 brothers (age 48 and 56 y/o at last determination) who were found to be heterozygotes for the c.951G>A variant (p.Met317Ile) (40). This variant was reported in an homozygotes subject with FED (table S2) but the study of this variant *in vitro* provided conflicting results (table S10). Despite being heterozygotes, these 2 individuals showed signs of LCAT deficiency (FLD, and FED, respectively). The younger brother displayed proteinuria at age 38 (84), he underwent his first renal biopsy at age 44 and showed severe impairment of renal function, with initiation of hemodialysis at age 48 (40, 84). In both cases LCAT activity, EC%, HDL-C and ApoA-I levels were similarly reduced. However, the younger brother had higher total cholesterol and apoB levels and displayed mild glucose intolerance whereas the older brother was on vegetarian, low-fat diet (84). The presence of a second yet to be identified variant in *LCAT* or in another gene, or the presence of other non-genetic factors may have contributed to the reported phenotype.	(40, 84)
4	Cases with uncertain genotype-phenotype relationship	The proband (male 53 y/o) was a heterozygotes carrier of the *LCAT* c.1289C>T (p. Pro430Leu) variant, never described in homozygotes subjects. He suffered from hematuria and proteinuria since age 24. At age 30 he was diagnosed with IgA glomerulonephritis. At age 35 he started dialysis and he subsequently underwent 2 renal transplantations. His laboratory values at the time of the second transplantation (age 53) showed significantly reduced HDL-C, LCAT mass and LCAT activity levels and increased TG levels. Interestingly	(81)

Family	Category	Description	Ref
		all these parameters improved after transplantation, although the levels of TG, LDL-C and LCAT activity towards endogenous lipoproteins were not fully normalized. His sister (age 63) was also heterozygous for the same mutation. Her lipid profile was normal, and she did not display renal disease. Finally, his son (age 12) was heterozygous for both *LCAT* c.1289C>T (p. Pro430Leu) and an *APOB* variant. His lipid profile was consistent with hypobetalipoproteinemia and he did not show any sign of renal disease. Overall, these data suggest that other factors, rather than the LCAT variant, have led to the clinical presentation of this case.	
5	Cases with uncertain genotype-phenotype relationship	This is a large kindred that included 9 homozygotes carriers, 57 heterozygous carriers of the c.820 C>T (p.Pro274Ser) LCAT variant and 44 non-carriers. The homozygotes displayed the classical FLD phenotype and are included in the analysis described in the main text. Among the heterozygotes, 3 displayed corneal opacity, 35 experienced hematuria and 21 proteinuria. 18 heterozygous subjects developed renal events, of whom 2 reached ESRD. None of the non-carrier family controls displayed corneal opacities, but 9 experienced hematuria and 5 experienced proteinuria. Interestingly, corneal opacity, low HDL-C levels and reduced eGFR, co-occurred in one heterozygous subject thus making this case atypical and compatible with our clinical definition of FLD (data provided by KS). The high prevalence of renal disease in this family suggests that other factors, genetic or not, other than or in addition of the presence of the LCAT variant may have contributed to the observed phenotypes.	(39)
6	Cases with intermediate clinical phenotype	This subject (woman, 28 y/o at last determination) displayed edema, anemia, proteinuria and modestly decreased renal function (78 ml/min/1.73 m^2). Renal biopsy revealed the presence of the typical features of FLD. Despite these traits, the subject did not display corneal opacity. Laboratory analyses showed significantly decreased LCAT activity levels with surprisingly normal HDL-C values. A genetic confirmation of the diagnosis was not available. The absence of corneal opacity and the presence of normal HDL-C levels indicates an abnormal clinical phenotype, perhaps compatible with acquired LCAT deficiency.	(85)
7	Cases with intermediate clinical phenotype	This subject (woman, 11 y/o at last examination) was first admitted to the hospital when she was 7 y/o with signs of precocious puberty. When she was 11, very low levels of HDL-C were observed and genetic testing revealed the presence of two different LCAT variants, one leading to premature protein truncation (c.284-285del, p.Val96ArgfsTer6) and the second inducing a single amino acid substitution (c.476G>A, p.Arg159Gln). The latter variant has also been identified in a compound heterozygote with FED phenotype (table S3 and S11). At clinical examination, she did not display corneal opacity or renal disease, but her lipid and lipoprotein profile were consistent with LCAT deficiency. It is possible that this subject was still in a pre-symptomatic state.	(64)
8	Cases with intermediate clinical phenotype	This subject (man, 28 y/o at last examination) was an homozygotes carrier of the c.1267C>T (p.R423C) variant, previously identified in 2 compound heterozygous subjects with FLD phenotype (table S3 and S11). At age 28, he did not display corneal opacity or renal abnormalities, but he had anemia, significantly reduced HDL-C and LCAT activity levels. *In vitro* expression of this variant showed that the LCAT activity on HDL-like substrate (measuring α activity) for the mutant protein was negligible (57).	(37)

Family	Category	Description	Ref
9	Carriers of additional variants on genes known to affect lipid metabolism	The case (male, 32 y/o) was a heterozygous carrier of the *LCAT* c.512G>A (p.Arg171Gln) variant, reported to be FLD-causing (table S2 and S9) and a second variant in the LPL gene. He did not display any sign of LCAT deficiency or cardiovascular disease. The levels of HDL-C and ApoA-I levels were decreased, whereas the levels of TG were increased.	(43)
10	Carriers of additional variants on genes known to affect lipid metabolism	The proband (man, 48 y/o), his father (75 y/o) and his son (23 y/o) were heterozygous carriers of two different mutations: *LCAT* c.803G>A (p.Arg268His), also identified in an FLD patient (table S3 and S11) and a variant in the *LDLR* gene. None of the cases displayed any sign of LCAT deficiency. The proband had signs of pre-clinical atherosclerosis whereas his father had myocardial infarctions at the ages of 53 and 62. The lipid and lipoprotein profile in all cases reflected the presence of both variants and showed reduced HDL-C, LCAT concentration, LCAT activity levels with increased levels of LDL-C and apoB.	(86)
11	Carriers of additional variants on genes known to affect lipid metabolism	This case (man, 30 y/o) presented with mild corneal opacification. There were no signs of renal disease, coronary heart disease, or anemia, although the erythrocyte morphology was altered. Genetic analysis revealed the presence of the c.694T>A and c.1216T>G (p.Ser232Thr; Phe406Val, respectively) *LCAT* variants on one allele and the c.1034C>T (p.Thr345Met) variant on the second allele. The clinical significance of the c.694T>A and c.1216T>G variants are uncertain. c.694T>A occurs at a relatively high frequency in the population, and it is likely benign. c.1216T>G has never been identified in homozygote subjects but its expression in cell systems suggests that it may abolish activity towards HDL particles (table S11). Finally, the c.1034C>T variant was associated with FLD (table S2 and S10). Furthermore, this subject was also heterozygous for a mutation in the *APOB* gene, reported to cause familial hypercholesterolemia. He presented with normal levels of total cholesterol, impaired levels of LCAT activity, HDL-C and EC% and increased levels of TG. LpX was present. His lipoprotein phenotype appeared to be mostly affected by lack of LCAT activity rather than defective apoB and it was suggestive of FLD. However, at the time of publication (age 30 y/o) the subject did not display signs of renal disease.	(74)

.

Table S5. Transplantation outcome at follow up

Subject	Number of Transplants	Age at Transplant	Outcome				Ref
			Failure/Rejection	Preserved kidney function	Histologic signs of recurrence	Follow-up Age (time from transplant)	
1	1	27	✓		ND	37 (10 years)	(87)
2	1	34	✓		Unclear	39 (5 years)	(50)
3	1	47	✓		ND	47 (6 months)	(88)
4	1	44	✓		ND	ND	(89)
5[a]	1	29		✓	✗	33 (4 years)	(71)
6	1	64		✓	ND	66 (26 months)	(33)
7	1	35		✓	ND	ND	(90)
8	2	28<br>37	✓ 	 ✓	ND<br>ND	ND<br>46 (9 years)	(91)
9	1	40		✓	✓	45 (5 years)	(38)
10	1	43		✓<br>✓	✓<br>ND	55 (12 years)<br>57 (14 years)	(38)
11	1	28		✓<br>✓<br>✓	✓<br>✓<br>✓	28 (2 days)<br>28 (6 weeks)<br>29 (1 year)	(75)
12	1	45		✓<br>✓<br>✓<br>✓	✗<br>✓<br>ND<br>ND	45 (3 weeks)<br>48 (40 months)<br>49 (48 months)<br>50 (56 months)	(92)
13[b]	1	39		✓	✓	39 (6 months)	(89, 93, 94)
14	1	55		✓<br>✓	✓<br>ND	56 (14 months)<br>61 (6 years)	(89, 94)
15	1	51		✓	✓	60 (9 years)	(95)
16	1	24		✓	✓	28 (4 years)	(96)
17	1	41		✓<br>✓	✓<br>ND	43 (20 months)<br>45 (4 years)	(97)
18	1	42		✓	✓	44 (2 years)	(89)
19	1	ND	ND	ND	ND	ND	(53)
20	1	39	ND	ND	ND	ND	(72)

Abbreviations: ✓ indicates presence of indicated trait, ✗ indicates absence of indicated trait, ND, not determined. Preserved kidney function indicates residual kidney function that didn't require permanent restoration of dialysis. Histologic signs of recurrence indicate biopsy findings characteristic of FLD. Age at follow-up is indicated in years. [a] Subject underwent a sequential liver-kidney transplantation. [b] Subject died after 7 months because of Tuberculosis infection. Data refer to post-mortem biopsy of transplanted kidney.

Table S6. Kidney Histologic findings in single FLD patients

Subject	1	2	3	4	5	6	7	8	9	10	11	12	13	14	15	16	17	18	19	20	21	22	23	24	25	26	27	28	29
Reference	(51)	(14)	(98, 99)	(13)	(13)	(26)	(100)	(101)	(40, 42)	(40)	(40)	(102)	(76)	(60)	(15)	(15)	(66)	(38)	(103)	(104)	(17)	(17)	(17)	(105, 106)	(53)	(39)	(39)	(39)	(39)
Global and/or segmental glomerular sclerosis						✓	✓							✓		✓	✓									✓	✗	✓	✓
Vacuolization of glomerular cells																										✗	✗	✓	✓
Foam cell infiltration		✓	✓						✓			✓								✗	✗			✓		✓	✗	✗	✓
Macrophage infiltration							✓																			✓	✗	✓	✗
Mesangial matrix expansion		✓					✓		✓	✓	✓		✓	✓	✓			✓			✓	✓				✗	✗	✓	✗
Mesangial deposits	✓			✓	✓					✓	✓		✓	✓	✓				✓	✓	✓								
Vacuolization of mesangial area						✓	✓	✓	✓			✓		✓				✓											
Mesangial foam cell infiltration		✓																	✓		✗								
Thickening of the GBM		✓	✓			✓		✓				✓	✓		✓			✓	✓		✓			✓		✓	✓	✓	✓
Duplication of the GBM		✓																								✗	✗	✗	✓
Vacuolization of GBM	✓	✓	✓	✓	✓	✓			✓			✓	✓	✓						✓				✓	✓	✗	✗	✗	✓
Lipid deposits in GBM				✓	✓									✓	✓						✗							✓	✓
Thickened capillary walls							✓						✓	✓					✗										
Pericapillary depositions	✓						✓		✓																				
Effacement of podocyte foot processes												✓							✓					✓					
Tubulo-interstitial changes						✓						✓							✗										
Tubular atrophy							✓							✓		✓			✗							✓	✓	✓	✓
Interstitial fibrosis							✓											✓								✓	✗	✗	✓
Tubular cell vacuolization																										✓	✗	✗	✗
Thickening of TBM													✓	✓															
TBM vacuolization																										✗	✗	✗	✗
Interstitial inflammation																										✓	✗	✓	✓
Interstitial edema																										✗	✓	✗	✗
Interstitial macrophages																										✓	✓	✓	✓
Intimal thickening																										✓	✗	✓	✓
Hyalinosis																										✓	✓	✓	✓
Pseudo-thrombi																										✓	✓	✓	✗
Subendothelial deposits																													
IgA		✗					✗																			✗	✗	✗	✗
IgG		✗					✗																			✗	✗	✗	✗
IgM		✗					✗																			✗	✓	✗	✓
C3		✗					✓						✓	✓						✓						✓	✓	✗	✗
C4		✗					✗																			✗	✗	✗	✗
C1q		✗					✓																			✗	✓	✗	✗

Subject	30	31	32	33	34	35	36	37	38	39	40	41	42	43	44	45	46	47	48	49	50	51	52	53	54	55	56	57	58
**Reference**	(39)	(39)	(49, 50)	(75)	(62)	(107)	(108)	(109)	(110)	(92)	(93, 111-113)	(94, 112)	(114)	(115)	(88)	(88)	(95)	(116)	(117)	(118)	(73)	(96)	(97)	(33, 119)	(90)	(120)	(31)	(9)	(121)
**Global and/or segmental glomerular sclerosis**	✓	✓		✓	✓	✓										✗					✓		✓		✓	✓		✓	
**Vacuolization of glomerular cells**	✓	✓														✓				✓									
**Foam cell infiltration**	✓	✓		✗		✓			✓		✓			✓		✓		✓				✓		✓		✓		✓	✓
**Macrophage infiltration**	✗	✗																											
**Mesangial matrix expansion**	✓	✓	✓			✓	✓								✓	✓	✓	✓	✓				✓	✓		✓	✓		
**Mesangial deposits**				✓	✓	✓					✓	✓	✓	✓	✓	✓	✓		✓				✓			✓	✓	✓	
**Vacuolization of mesangial area**				✓		✓					✓						✓						✓			✓			
**Mesangial foam cell infiltration**				✗			✓							✓		✓		✓											
**Thickening of the GBM**	✓	✓					✓				✓			✓		✓			✓	✓						✓			
**Duplication of the GBM**	✓	✓					✓													✓									
**Vacuolization of GBM**	✓	✓	✓	✓		✓					✓			✓		✓		✓	✓		✓				✓				
**Lipid deposits in GBM**	✓	✓			✓	✓						✓				✓			✓							✓			
**Thickened capillary walls**			✓	✓		✓								✓				✓			✓		✓			✓	✓		
**Pericapillary depositions**														✓		✓						✓				✓	✓	✓	
**Effacement of podocyte foot processes**				✓							✓		✓			✓	✓			✓									
**Tubulo-interstitial changes**														✓							✓			✗					✗
**Tubular Atrophy**	✓	✓		✓												✗		✓					✓			✓			
**Interstitial Fibrosis**	✓	✓		✓																	✓		✓			✓			✗
**Tubular cell vacuolization**	✓	✓																											
**Thickening of TBM**											✓																		
**TBM Vacuolization**	✓	✓																							✓				
**Interstitial inflammation**	✓	✓																											✗
**Interstitial edema**	✗	✗																											✗
**Interstitial macrophages**	✓	✓																											
**Intimal thickening**	✗	✓																											
**Hyalinosis**	✗	✓									✓			✓						✓									✓
**Pseudo Thrombi**	✗	✓		✓																						✗			✓
**Subendothelial deposits**				✓									✓	✓		✓								✓		✓		✓	
**IgA**	✗	✗		✗															✗	✗					✗	✗			✗
**IgG**	✗	✗	✓	✗															✗	✗					✗	✗			✗
**IgM**	✓	✓		✓			✓												✗	✗					✓	✓			✗
**C3**	✓	✓		✗			✓												✗							✓			✗
**C4**	✓	✓		✗															✗							✓			✗
**C1q**	✓	✓					✓												✗							✗			

Abbreviations: GBM, glomerular basement membrane; TBM, tubular basement membrane; IgA, immunoglobulin A; IgG, immunoglobulin G; IgM, immunoglobulin M; C3, complement protein C3; C4, complement protein C4; C1q, complement protein C1q; ✓, presence of indicated trait; ✗, absence of indicated trait.

Table S7. Summary of observational studies of CVD risk in FLD and FED patients and heterozygous subjects.

Study (Ref)	Study groups	Number of subjects	Measured CVD-related outcome [a]	Results [b]	Notes
1 (122)	• Carriers: FLD-Het + FED-Het (Various variants)<br>• Controls: Non-carriers	• 47<br>• 58	• Pre-clinical atherosclerosis (Carotid Ultrasonography)<br>• CAD events (Acute myocardial infarction, percutaneous coronary intervention, coronary artery bypass grafting, angina pectoris, peripheral or cerebrovascular disease)	Mean IMT was higher in Carriers vs controls. 2 carriers and 0 controls had a CAD event.	The subjects were family members of FED patients from 5 different families.
2 (123)	• Carriers: FLD + FED + FLD-Het + FED-Het (Various variants)<br>• Controls: Non-carriers	• 40 (9 FLD, 3 FED, 24 FLD-Het, 4 FED-Het)<br>• 80	Pre-clinical atherosclerosis (Carotid Ultrasonography)	Average IMT and maximum IMT were decreased in Carriers vs Controls.	Most of included subjects are FLD and FLD-Het.<br>Subjects included in the study may overlap across studies 2, 3, 7, 8.
	• Carriers 1: FLD + FED (Various variants)<br>• Carriers 2: FLD-Het + FED-Het (Various variants)<br>• Controls: Non-carriers	• 12 (9 FLD, 3 FED)<br>• 28 (24 FLD-Het, 4 FED-Het)<br>• 80	Pre-clinical atherosclerosis (Carotid Ultrasonography)	All IMT measurements were decreased in allele-dependent fashion (Carriers 1 < Carriers 2 < Controls).	
	• Carriers 1: FLD + FLD-Het (Various variants)<br>• Carriers 2: FED+FED-Het (Various variants)	• 33 (9 FLD, 24 FLD-Het)<br>• 7 (3 FED, 4 FED-Het)	Pre-clinical atherosclerosis (Carotid Ultrasonography)	IMT was comparable in Carriers 1 vs Carriers 2.	
3 (124)	• Carriers 1: FLD + FED (Various variants)<br>• Carriers 2: FLD-Het + FED-Het (Various variants)<br>• Controls: Non-carriers	• 14 (8 FLD, 6 FED)<br>• 27<br>• 10	Serum cholesterol efflux capacity	Estimated SR-BI mediated cholesterol efflux capacity was reduced in Carriers 1 compared to Carriers 2 and controls.<br>Estimated ABCG1 mediated cholesterol efflux capacity was reduced in Carriers 1 and 2 compared to controls.<br>Estimated ABCA1- mediated cholesterol efflux capacity was increased in Carriers 1 and 2 compared to controls.	Subjects included in the study may overlap across studies 2, 3, 7, 8.
4 (125)	• Carriers: FED + FLD-Het + FED-Het + unknown clinical significance (Various variants)<br>• Controls: Non-carriers	• 40 (2 FED, 4 FLD-Het, 31 FED-het, 3 NA-Het)<br>• 40	Pre-clinical atherosclerosis (Carotid Ultrasonography, Carotid Magnetic Resonance Imaging, MRI)	Mean wall area, normalized wall index, and total wall volume were increased in Carriers vs Controls (Carotid MRI).<br>IMT was comparable in Carriers vs Controls (Carotid Ultrasonography)	Subjects included in the study may overlap across studies 4, 5 and 8.<br>Most included subjects are FED-Het.<br>The study includes carriers of variants of unknown clinical significance (NA).

Study (Ref)	Study groups	Number of subjects	Measured CVD-related outcome [a]	Results [b]	Notes
5<br>(126)	• FED + FLD-Het + FED-Het + unknown clinical significance (Various variants)<br>• Controls: Non-carriers	• 45 (2 FED, 43 Het carriers of any LCAT variant)<br>• 45	Pre-clinical atherosclerosis (Carotid-femoral pulse wave velocity, PWV)	PWV was higher in Carriers vs Controls.	Subjects included in the study may overlap across studies 4, 5, 8.<br>The study includes carriers of variants of unknown clinical significance (NA).
6<br>(127)	• Carriers 1: FLD-Het + FED-Het + unknown clinical significance (Various variants)<br>• Controls: Non-carriers	• 28, with 29 variants (5 FLD-causing, 17 FED-causing, 7 NA*)<br>• 149	CAD events (Angina pectoris or myocardial infarction)	Increased CAD odds ratio in the subset of carriers with HDL-C < 5th percentile of the control population.	Carriers were identified in a population with HDL-C<10th percentile of the control population.<br>The study includes carriers of variants of unknown clinical significance (NA).
7<br>(128)	• Carriers 1: FLD (Various variants)<br>• Carriers 2: FLD-Het + FED-Het (Various variants)<br>• Controls: Non-carriers	• 6 (pooled)<br>• 10<br>• 10	Vascular endothelial function (HDL-induced eNOS expression and activation levels, HDL-induced inhibition of TNFα-mediated VCAM-1 expression)	Allele-dependent increase of eNOS activation without difference in expression. Allele-dependent decrease of TNFα-mediated VCAM-1 expression.	Subjects included in the study may overlap across studies 2, 3, 7, 8.<br>Most included subjects are FLD or FLD-Het.
	• Carriers 1: FLD (Various variants)<br>• Carriers 2: FLD-Het + FED-Het (Various variants)<br>• Controls: Non-carriers	• 15 (10 FLD, 5 FED)<br>• 60 (48 FLD-het, 12 FED-Het)<br>• 32	Vascular endothelial function (soluble VCAM-1, ICAM-1, and E-selectin plasma levels).	VCAM-1, ICAM-1, and E-selectin plasma levels were comparable across groups	
	Carriers 1: FLD + FLD-Het + FED-Het (Various variants)<br>• Controls: Non-carriers	• 15 (5 FLD, 7 FLD-Het, 3 FED-Het)<br>• 35	Flow mediated vasodilation, FMD	FMD was comparable in Carriers vs Controls	
8<br>(129)	• Carriers 1: FLD-Het (Various variants)<br>• Carriers 2: FED-Het (Various variants)<br>• Controls: Non-carriers	• 33<br>• 41<br>• 280	Pre-clinical atherosclerosis (Carotid Ultrasonography)<br>History of CVD	Average IMT and maximum IMT were decreased in Carriers 1 vs Carriers 2 and in Carriers 1 vs Controls.<br>2/33 subjects from Carrier 1, 5/41 subjects from Carrier 2 and 8/280 controls had personal history of CVD.	Subjects included in the study may overlap across studies 2, 3, 4, 5, 7, 8,
	• Carriers 1: FLD + FLD-Het (Various variants)<br>• Carriers 2: FED + FED-Het (Various variants)<br>• Controls: Non-carriers	• 44 (11 FLD, 33 FLD-Het)<br>• 46 (5 FED, 41 FED-Het)<br>• 280	Pre-clinical atherosclerosis (Carotid Ultrasonography)<br>History of CVD	Average IMT and maximum IMT were decreased in Carriers 1 vs Carriers 2 and in Carriers 1vs Controls.<br>2/44 subjects from Carrier 1, 6/46 subjects from Carrier 2 and 8/280 controls had personal history of CVD.	

Study (Ref)	Study groups	Number of subjects	Measured CVD-related outcome [a]	Results [b]	Notes
	• Carriers 1: FLD (Various variants)<br>• Carriers 2: FED (Various variants)<br>• Controls: Non-carriers	• 11<br>• 5<br>• 280	History of CVD	0/11 subjects from Carrier 1, 1/5 subjects from Carrier 2 and 8/280 controls had personal history of CVD.	

Abbreviations: [a] CAD, Coronary Artery Disease; SR-BI, scavenger receptor class B type I; ABCG1, ATP Binding Cassette Subfamily G Member 1; ABCA1, ATP Binding Cassette Subfamily A Member 1; eNOS, endothelial nitric oxide synthase; TNFα, Tumor Necrosis Factor alpha; VCAM-1, Vascular Cell Adhesion Molecule 1; ICAM-1, Intercellular Adhesion Molecule 1; [b] IMT, Intima-Media Thickness.

Table S8. Additional lipid and lipoprotein parameters

Biomarker	Subject group				FLD vs FED p-value	FLD-Het vs FED-Het p-value	FLD vs FLD-Het p-value	FED vs FED-Het p-value	Ref values
	FLD	FED	FLD-Het	FED-Het					
Phospholipids									
Median	218.6	71.5	140.4	ND	0.1070	ND	0.0481	ND	125 – 275
IQR	127.0-301.0	-	86.4-243.0	ND					
N	42	1	18	0					
ApoC-III									
Median	4.4	4.5	7.7	8.4	0.5410	0.4795	0.1701	0.1213	4.5-11
IQR	3.6-8.5	2.8-5.2	6.3-8.6	8.1-8.7					
N	15	5	9	2					
Apo E									
Median	8.6	6.6	3.3	4.0	0.3554	0.6227	<0.0001	0.4018	3-7
IQR	6.2-11.0	4.2-11.5	2.9-4.5	3.1-4.8					
n	22	6	16	2					
Lp(a)									
Median	1.2	10	6.2	11.4	0.0024	0.0081	<0.0001	0.1096	<30
IQR	1.0-3.0	3.0-34.0	4.1-23.1	10.1-11.4					
N	29	7	57	38					

Abbreviations: FLD indicates subjects with Familial LCAT deficiency phenotype; FED indicates subjects with Fish-eye disease phenotype; FLD-Het indicates heterozygous carriers of a FLD-causing variant; FED-Het indicates heterozygous carriers of a FED-causing variant; NA, not applicable. Reference values refer to thresholds and intervals determined in non-affected healthy individuals. Lipid and lipoproteins are expressed in mg/dL, Statistical comparisons were determined using Wilcoxon Rank Sum test.

Table S9. Linear Regression Analysis of LCAT activity, EC% and UC levels with FLD/FED phenotypes and LCAT concentration.

Parameter	Multivariate analysis	
	Estimate coefficient (SE), N	P-value
**LCAT activity towards endogenous lipoproteins (% of control)**	-46.3847 (5.2021), 67	<0.0001
**LCAT activity towards exogenous substrate (% of control)**	-5.0078 (1.4870), 65	0.0013
**Unesterified Cholesterol (UC) (mg/dL)**	55.9272 (22.1721), 71	0.0140
**Esterified Cholesterol (% of total Cholesterol), EC%**	-42.9373 (3.0900), 76	<0.0001

Abbreviations: SE, standard error. N indicates the number of subjects for which data were available. Linear regression was performed using LCAT activity towards endogenous lipoproteins, LCAT activity towards exogenous substrates, unesterified cholesterol or Esterified Cholesterol (% of Total Cholesterol) levels as dependent variables. FLD or FED phenotype and plasma LCAT concentration were used as explanatory variables. Non-standardized regression coefficients and P values refer to the to the change in indicated parameters explained by FLD phenotype, accounting for plasma LCAT concentration.

Table S10. Association of clinical phenotype with predicted and observed enzymatic activity in homozygous subjects

Carrier type	Carrier Clinical Classification	Variant		Predicted effect on enzymatic activity	Observed effect on enzymatic activity
		DNA change	Protein change	Expression of recombinant protein in cell systems	Homozygotes patient's plasma
HO	FLD	c.31del	p.Val11Ter (1)	Complete deficiency**	Complete deficiency (1)
HO	FED	c.86A>G	p.Asn29Ser (2)	ND	ND
HO	FLD	c.86A>T	p.Asn29Ile (3)	ND	Complete deficiency (3)
HO	FED	c.101C>T	p.Pro34Leu (4)	Complete/partial deficiency (residual β activity) (130, 131)	Partial deficiency (residual β activity) (4, 132, 133)
HO	FLD	c.101insC	p.His35AlafsTer7 (5)	Complete deficiency**	Complete deficiency (102, 134, 135)
HO	FLD	c.102del	p.His35ThrfsTer26 (6)	Complete deficiency**	Complete deficiency (6)
HO	FED	c.110C>T	p.Thr37Met (7, 8)	Partial deficiency (partial α activity) (7)	Partial deficiency (residual α/β activity) (7, 8)
HO	FLD	c.154+1G>C [g.5166G>C]	NA (intronic) (9)	ND	ND
HO	FLD	c.160G>A	p.Gly54Ser (10-12)	ND (loss of α activity) (11, 136)	Complete deficiency (10-12)
HO	FED	c.209T>A	p.Val70Glu (1)	ND	Partial deficiency (residual β activity) (1)
HO	FLD	c.278C>T	p.Pro93Leu (13)	ND	Partial deficiency (residual α activity) (13)
HO	FLD	c.293G>A	p.Cys98Tyr (10, 14)	ND	Complete deficiency (14)
HO	FLD	c.295T>C	p.Trp99Arg (15)	ND	Complete deficiency (15)
HO	FLD	c.301G>A	p.Asp101Asn (16)	ND	ND
HO	FLD	c.321C>A	p.Tyr107Ter (17)	Complete deficiency**	Complete deficiency (17)
HO	FLD	c.349G>A*<br>c.544C>T*	p.Ala117Thr*<br>p.Arg182Cys* (17, 18)	Complete deficiency (131, 137)<br>Complete/partial deficiency (residual α /β activity) (130, 131, 137)	Complete deficiency (17, 18)
HO	FLD	c.356G>A	p.Gly119Asp (19)	ND	Complete deficiency (19)
HO	FED	c.367C>T	p.Arg123Cys (20)	Complete deficiency (20)	Partial deficiency (residual α/β activity) (20)
HO	FLD	c.368G>C	p.Arg123Pro (21)	ND	Complete deficiency (21)
HO	FED	c.440C>T	p.Thr147Ile (10, 22, 23)	Partial deficiency (residual β activity) (130, 131, 138-142)	Partial deficiency (residual β activity) (10, 22, 23)
HO	FED	c.463A>G	p.Asn155Asp (24)	Partial deficiency (residual β activity) (140, 142)	Partial deficiency (residual β activity) (24)
HO	FED	c.488_489del	p.Val163AlafsTer6 (25)	Complete deficiency**	Complete deficiency (25)
HO	FLD	c.490C>T	p.Arg164Cys (26)	ND	Complete deficiency (26)
HO	FLD	c.491G>A	p.Arg164His (27)	Complete deficiency (27, 63, 136)	ND (loss of α activity) (27, 143)
HO	FLD	c.493insGGC	p.Arg164_Ala165insGly (28)	ND	Complete deficiency (28, 98, 99)
HO	FLD	c.511C>T	p.Arg171Trp (1, 29, 30)	Complete deficiency (30, 123, 130, 131, 136)	Complete deficiency (1, 29, 30, 104, 144)
HO	FLD	c.512G>A	p.Arg171Gln (31)	ND	Complete deficiency (31)
HO	FED	c.544C>T	p.Arg182Cys (32)	Partial deficiency (residual β activity) (130, 131, 137)	Complete deficiency (32)
HO	FLD	c.604insA	p.Ile202AsnfsTer27 (33)	Complete deficiency**	Complete deficiency (33, 119)
HO	FLD	c.605T>C	p.Ile202Thr (31)	ND	Complete deficiency (31)
HO	FLD	c.607G>C	p.Gly203Arg (10, 34)	ND (loss of α activity) (34)	ND, (loss of α activity) (10, 34)
HO	NA	c.694T>A	p.Ser232Thr (35)	ND	ND
HO	FLD	c.698T>C	p.Leu233Pro (17, 36)	Complete deficiency (131, 136)	Complete deficiency (17, 110)
HO	FLD	c.726G>C	p.Lys242Asn (1)	Complete deficiency (123)	Complete deficiency (1)
HO	FLD	c.756C>A	p.Asn252Lys (28)	Complete deficiency (130, 138)	Complete deficiency (28, 101, 145-147)

Carrier type	Carrier Clinical Classification	Variant		Predicted effect on enzymatic activity	Observed effect on enzymatic activity
		DNA change	Protein change	Expression of recombinant protein in cell systems	Homozygotes patient's plasma
HO	FLD	c.760G>C	p.Gly254Arg (37)	Partial deficiency (37)	Complete deficiency (37)
HO	FLD	c.803G>T	p.Arg268Leu (38)	ND	ND
HO	FLD	c.820 C>T	p.Pro274Ser (39)	Complete deficiency (39, 139)	Complete deficiency (39)
HO	FLD	c.821C>G	p.Pro274Arg (40)	ND	Complete deficiency (40)
HO	FLD	c.827T>A	p.Met276Lys (41)	ND	Complete deficiency (41, 148)
HO	FLD	c.862del	p.Val288CysfsTer122 (40, 42)	Complete deficiency (42)	Complete deficiency (40, 42)
HO	FLD	c.893C>T	p.Thr298Ile (1)	Complete deficiency (123)	Complete deficiency (1)
HO	FLD	c.950T>C	p.Met317Thr (13)	ND	Partial deficiency (residual α activity) (13)
HO	FLD	c.950T>G	p.Met317Arg (43-45)	ND	Complete deficiency (44)
HO	FED	c.951G>A	p.Met317Ile (28, 46)	Complete deficiency (130)	Partial deficiency (residual β activity)(28, 46, 149, 150)
HO	FED	c.970_972del	p.Leu324del (47, 48)	Complete/partial deficiency (48, 130)	Partial deficiency (residual β activity) (47, 48, 151)
HO	FLD	c.997G>A	p.Val333Met (49, 50)	Complete deficiency (79, 123)	Complete deficiency (49, 50)
HO	FLD	c.1010G>A	p.Cys337Tyr (10, 51)	Complete deficiency (51)	Complete deficiency (10, 51)
HO	FLD	c.1034C>T	p.Thr345Met (17, 52, 53)	Complete deficiency (70, 131)	Complete deficiency (17, 53, 152-154)
HO	FED	c.1052 A>G	p.Tyr351Cys (2)	ND	ND
HO	FLD	c.1102G>A	p.Gly368Ser (40, 42)	ND (loss of α activity) (42, 136)	Complete deficiency (40, 42)
HO	FLD	c.1187T>G	p.Leu396Arg (1, 54)	Complete deficiency (123)	Complete deficiency (1, 54)
HO	Unclassified	c.1267C>T	p.Arg423Cys (37)	Complete deficiency (57)	Complete deficiency (37)

Abbreviations: HO, homozygous subject; FED indicates subjects with Fish-eye disease phenotype; FLD indicates subjects with Familial LCAT deficiency phenotype; Unclassified indicates subjects who could not be clearly classified as FLD or FED; NA, indicates absence of FLD or FED phenotypes; ND, not determined; β activity, activity towards apoB-containing particles. Variants are described according to the guidelines of Human Genome Variation Society (55) and using (Reference GenBank numbers NG_009778, genomic sequence, NM_000229, coding sequence and their location in the protein sequence, 440 amino acids). Ter indicates termination codon. For frameshift mutations, the first amino acid changed in the mutant protein is indicated. The position of the translation termination codon in the new reading frame is calculated starting at the first amino acid changed. Predicted effect on enzymatic activity was estimated based on activity and protein levels obtained by expression of recombinant proteins in cell systems. Observed effects on enzymatic activity were based on the measurement of LCAT activity and concentration in carrier's plasma. For both assessments, activity and protein levels lower than 25% were considered negligible. *The variants were identified on the same allele. **The activity of variants presenting a premature termination codon was estimated to be negligible.

Table S11. Association of clinical phenotype with predicted and observed enzymatic activity in compound heterozygous subjects

Carrier type	Carrier Clinical classification	Variant		Predicted effect on enzymatic activity			Observed effect on enzymatic activity
		DNA change	Protein change	Expression of recombinant protein in cell systems	Homozygotes patient's plasma	Estimated Cumulative effect	Compound heterozygous patient's plasma
CH	FLD (56)	c.54_83dup<br>c.169G>C	p.Leu19_Leu28dup<br>p.Gly57Arg	ND<br>ND	ND<br>ND	ND	Complete deficiency (56)
CH	FED (65)	c.86A>T<br>c.1039C>T	p.Asn29Ile<br>p.Arg347Cys	ND<br>Partial deficiency (residual α activity) (65)	Complete deficiency (3)<br>ND	Partial deficiency	Partial deficiency (residual α activity) (65)
CH	FLD (57, 58)	c.96insC<br>c.1267C>T	p.His35AlafsTer7<br>p.Arg423Cys	Complete deficiency**<br>Complete deficiency (57)	ND<br>Complete deficiency (37)	Complete deficiency	Complete deficiency (57, 58)
CH	FED (59)	c.101C>A<br>c.476G>A	p.Pro34Gln<br>p.Arg159Gln	Partial deficiency (residual β activity) (59)<br>Complete deficiency (59, 136)	ND<br>ND	Partial deficiency	Partial deficiency (residual β activity) (59)
CH	FED (43, 155)	c.101C>T<br>c.110C>T	p.Pro34Leu<br>p.Thr37Met	Complete/partial deficiency (residual β activity) (130, 131)<br>Partial deficiency (partial α activity) (7)	Partial deficiency (residual β activity) (4, 132, 133)<br>Partial deficiency (residual α/β activity) (7, 8)	Partial deficiency	Partial deficiency (residual β activity) (155)
CH	FED (77)	c.110C>T<br>c.991C>T	p.Thr37Met<br>p.Pro331Ser	Partial deficiency (partial α activity) (7)<br>ND	Partial deficiency (residual α/β activity) (7, 8)<br>ND	Partial deficiency	Complete deficiency (77)
CH	FLD (1)	c.131_155+3del<br>c.490C>T	p.Asn44LysfsTer7<br>p.Arg164Cys	Complete deficiency**<br>ND	ND<br>Complete deficiency (26)	Complete deficiency	Complete deficiency (1)
CH	FLD (1, 60)	c.141_145del<br>c.614G>A	p.Pro48HisfsTer53<br>p.Ser205Asn	Complete deficiency**<br>Complete deficiency (123)	ND<br>ND	Complete deficiency	Complete deficiency (1, 60)
CH	FLD (61)	c.154G>A<br>c.703G>A	p.Val52Met<br>p.Ala235Thr	ND<br>ND	ND<br>ND	ND	Complete deficiency (61)
CH	FLD (15)	c.167T>C<br>c.802C>T	p.Leu56Pro<br>p.Arg268Cys	Complete deficiency (131, 136)<br>ND (loss of α activity) (65)	ND<br>ND	ND	Complete deficiency (15)
CH	FLD (62)	c.193del<br>c.602T>A	p.Asp65ThrfsTer6<br>p.Leu201His	Complete deficiency**<br>NA	ND<br>ND	ND	ND
CH	FLD (63)	c.283G>C<br>c. 491G>A	p.Gly95Arg<br>p.Arg164His	Complete deficiency (63)<br>Complete deficiency (27, 63, 136)	ND<br>ND (loss of α activity) (27, 143)	ND	ND (loss of α activity) (63)
CH	Unclassified (64)	c.284-285del<br>c.476G>A	p.Val96ArgfsTer6<br>p.Arg159Gln	Complete deficiency**<br>Complete deficiency (59, 136)	ND<br>ND	Complete deficiency	Complete deficiency (64)
CH	FED (10, 65)	c.296G>C<br>c.440C>T	p.Trp99Ser<br>p.Thr147Ile	ND (loss of α activity) (65)<br>Partial deficiency (residual β activity) (130, 131, 138-142)	ND<br>Partial deficiency (residual β activity) (10, 22, 23)	Partial deficiency	ND (loss of α activity) (10, 65)

Carrier type	Carrier Clinical classification	Variant		Predicted effect on enzymatic activity			Observed effect on enzymatic activity
		DNA change	Protein change	Expression of recombinant protein in cell systems	Homozygotes patient's plasma	Estimated Cumulative effect	Compound heterozygous patient's plasma
CH	FLD (66)	c.301G>A<br>c.388A>G	p.Asp101Asn<br>p.Thr130Ala	ND<br>ND	ND<br>ND	ND	ND<br>(loss of α activity) (66)
CH	FED (156)	c.321C>A<br>c.367C>T	p.Tyr107Ter<br>p.Arg123Cys	Complete deficiency**<br>Complete deficiency (20)	Complete deficiency (17)<br>Partial deficiency (residual α/β activity) (20)	Partial deficiency	ND
CH	FLD (69)	c.321C>A<br>c.538T>A	p.Tyr107Ter<br>p.Tyr180Asn	Complete deficiency**<br>Complete deficiency (69, 130, 136)	Complete deficiency (17)<br>NA	Complete deficiency	Complete deficiency (69)
CH	FLD (1, 76)	c.321C>A<br>c.892A>G	p.Tyr107Ter<br>p.Thr298Ala	Complete deficiency**<br>ND	Complete deficiency (17)<br>ND	ND	Complete deficiency (1, 76)
CH	FLD (157)	c.321C>A<br>c.1034C>T	p.Tyr107Ter<br>p.Thr345Met	Complete deficiency**<br>Complete deficiency (70, 131)	Complete deficiency (17)<br>Complete deficiency (17, 53, 152-154)	Complete deficiency	Complete deficiency (157)
CH	FED (1)	c.343T>C<br>c.493G>A	p.Ser115Pro<br>p.Ala165Thr	Complete deficiency (123)<br>Partial deficiency (residual β activity) (123)	ND<br>ND	Partial deficiency	Partial deficiency (residual β activity) (1)
CH	FED (43)	c.382G>A<br>c.491G>A	p.Gly128Ser<br>p.Arg164His	ND<br>Complete deficiency (27, 63, 136)	ND<br>ND (loss of α activity) (27, 143)	ND	ND
CH	FED (67)	c.440C>T<br>c.503A>G	p.Thr147Ile<br>p.Tyr168Cys	Partial deficiency (residual β activity) (130, 131, 138-142)<br>ND	Partial deficiency (residual β activity) (10, 22, 23)<br>ND	Partial deficiency	Partial deficiency (residual β activity) (67)
CH	FED (68)	c.440C>T<br>c.524-22T>C [g.6504T>C]	p.Thr147Ile<br>NA (intronic)	Partial deficiency (residual β activity) (130, 131, 138-142)<br>Complete deficiency (68, 158, 159)	Partial deficiency (residual β activity) (10, 22, 23)<br>ND	Partial deficiency	Partial deficiency (residual β activity) (68)
CH	FED (65)	c.440C>T<br>c.945G>A	p.Thr147Ile<br>p.Trp315Ter	Partial deficiency (residual β activity) (130, 131, 138-142)<br>Complete deficiency (65)	Partial deficiency (residual β activity) (10, 22, 23)<br>ND	Partial deficiency	ND<br>(loss of α activity) (65)
CH	FED (10)	c.440C>T<br>c.997G>A	p.Thr147Ile<br>p.Val333Met	Partial deficiency (residual β activity) (130, 131, 138-142)<br>Complete deficiency (79, 123)	Partial deficiency (residual β activity) (10, 22, 23)<br>Complete deficiency (49, 50)	Partial deficiency	ND<br>(loss of α activity) (10)
CH	FED (47, 78)	c.440C>T<br>c.1112C>T	p.Thr147Ile<br>p.Thr371Met	Partial deficiency (residual β activity) (130, 131, 138-142)<br>Complete deficiency (130, 131, 136)	Partial deficiency (residual β activity) (10, 22, 23)<br>ND	Partial deficiency	Partial deficiency (residual β activity) (47, 78)
CH	FLD (17)	c.475C>T<br>c.1198insG	p.Arg159Trp<br>p.Gln400AlafsTer41	Complete deficiency (131, 136)<br>Complete deficiency**	ND<br>ND	Complete deficiency	Complete deficiency (17, 106, 135, 160)
CH	FLD (72)	c.511C>T<br>c.585T>G	p.Arg171Trp<br>p.Tyr195Ter	Complete deficiency (30, 123, 130, 131, 136)<br>Complete deficiency**	Complete deficiency (1, 29, 30, 104, 144)<br>ND	Complete deficiency	Complete deficiency (72)

Carrier type	Carrier Clinical classification	Variant		Predicted effect on enzymatic activity			Observed effect on enzymatic activity
		**DNA change**	**Protein change**	**Expression of recombinant protein in cell systems**	**Homozygotes patient's plasma**	**Estimated Cumulative effect**	**Compound heterozygous patient's plasma**
CH	FLD (70, 71)	c.576del<br>c.1034C>T	p.His192GlnfsTer72<br>p.Thr345Met	Complete deficiency** (70)<br>Complete deficiency (70, 131)	ND<br>Complete deficiency (17, 53, 152-154)	Complete deficiency	Complete deficiency (70)
CH	FLD (73)	c.634insG<br>c.794_801del	p.Leu212AlafsTer16<br>p.Glu265AlafsTer18	Partial deficiency (residual α activity) (73)<br>Partial deficiency (residual α activity) (73)	ND<br>ND	Complete deficiency	Complete deficiency (73)
CH	Unclassified (74)	c.694T>A*<br>c.1216T>G*<br>c.1034C>T	p.Ser232Thr*<br>Phe406Val*<br>p.Thr345Met	ND<br>ND (Loss of α activity) (142)<br>Complete deficiency (70, 131)	ND (benign variant) (35)<br>ND<br>Complete deficiency (17, 53, 152-154)	Complete deficiency	Complete deficiency (74)
CH	FLD (75)	c.803G>A<br>c.827T>A	p.Arg268His<br>p.Met276Lys	Partial deficiency (residual β activity) (123)<br>ND	ND<br>Complete deficiency (41, 148)	Partial deficiency	ND
CH	FED (47)	c.827T>A<br>c.1244A>G	p.Met276Lys<br>p.Asn415Ser	ND<br>Partial deficiency (residual β activity) (140, 142)	Complete deficiency (41, 148)<br>ND	ND	Partial deficiency (residual β activity) (47, 161)
CH	FED (79)	c.997G>A<br>c.1210A>G	p.Val333Met<br>p.Met404Val	Complete deficiency (79, 123)<br>Complete deficiency (79)	Complete deficiency (49, 50)<br>ND	ND	Complete deficiency (79)
CH	FED (65)	c.1012C>T<br>c.1039C>T	p.Leu338Phe<br>p.Arg347Cys	Partial deficiency (residual α activity) (65)<br>Partial deficiency (residual α activity) (65)	ND<br>ND	Partial deficiency	Partial deficiency (residual α activity) (65)

Abbreviations: CH compound heterozygous subject; FED indicates subjects with Fish-eye disease phenotype; FLD indicates subjects with Familial LCAT deficiency phenotype. Variants are described according to the guidelines of Human Genome Variation Society (55) and using (Reference GenBank numbers NG_009778, genomic sequence, NM_000229, coding sequence and their location in the protein sequence, 440 amino acids). Ter indicates termination codon. For frameshift mutations, the first amino acid changed in the mutant protein is indicated. The position of the translation termination codon in the new reading frame is calculated starting at the first amino acid changed. The predicted effect of each variant on the enzymatic activity and their estimated cumulative effect are indicated. All the predictions were made based on activity and protein levels obtained by expression of recombinant proteins in cell systems and observed in plasma from homozygotes patients carrying the single mutations. Observed effect on enzymatic activity was based on the measurement of LCAT activity and concentration in the plasma of CH subjects. For all assessments, activity and protein levels lower than 25% of control value were considered negligible. *The variants were identified on the same allele. **The activity of variants presenting a premature termination codon was estimated to be negligible.

Figure S1: Geographical distribution of FLD and FED cases included in this study.

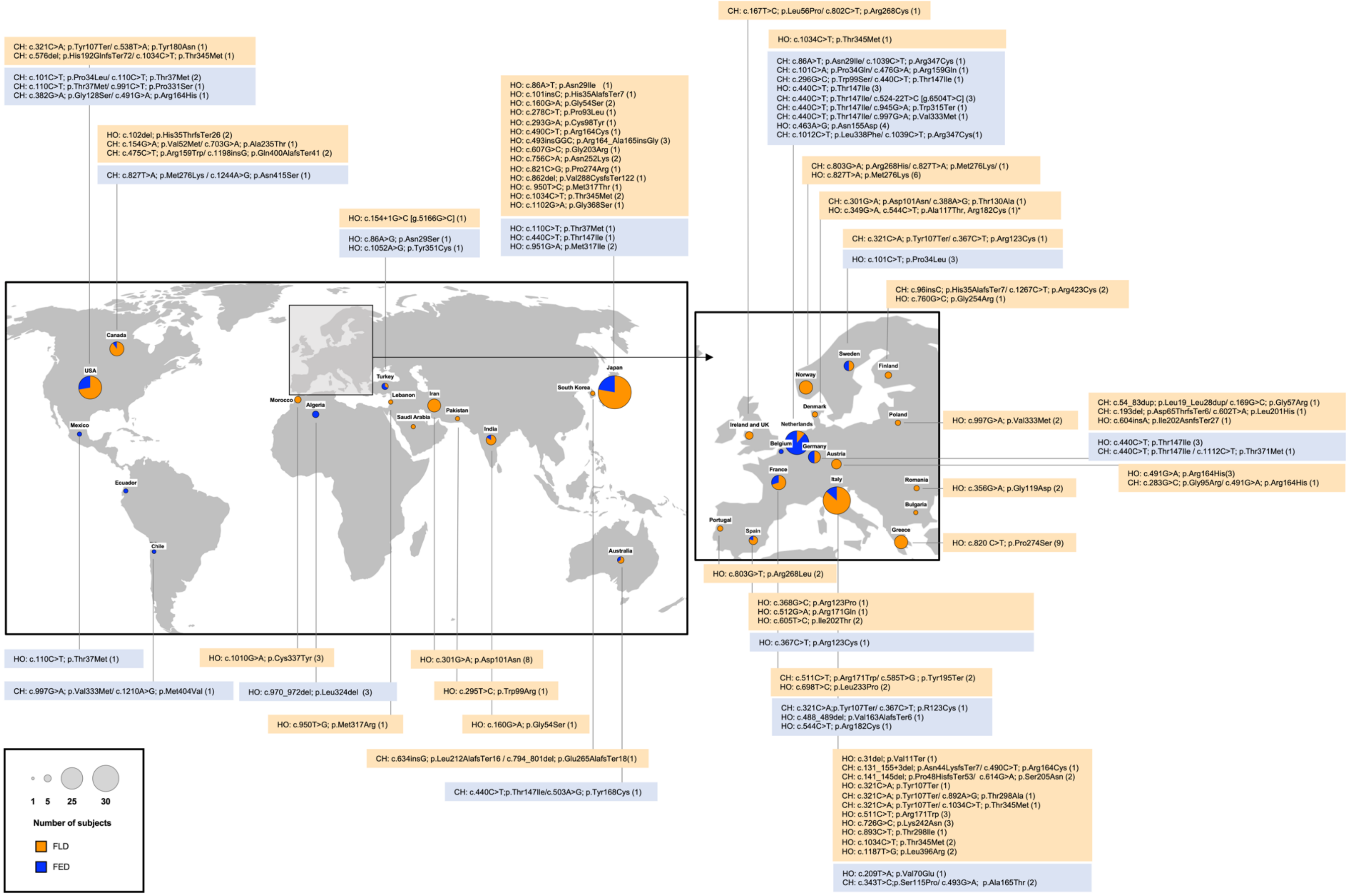

Cases were assigned to the country of provenience, or when non specified, the country where they were studied. Parts of whole graphs represent the proportion of the cases in each country. Orange color represents FLD cases, blue FED cases. Sphere size is proportional to the number of cases in each country. Genetic diagnosis is indicated as follows: Carrier status (HO: homozygous carrier, CH: compound heterozygous carrier), variant (consequence in coding sequence and protein sequence), number of carriers (indicated in parenthesis). Variants identified in FLD and FED patients are highlighted in orange and blue, respectively. *The variants were identified on the same allele.

Supplemental methods

1. Record search and selection

PubMed was systematically searched for key words related to LCAT deficiency from inception to April 22, 2020. Record screening and selection were performed by AB and CV, with conflicts resolved through discussion with MC.

The full list of terms used for the query was:

("Lecithin:Cholesterol Acyltransferase Deficiency"[All Fields] OR ("lecithin cholesterol acyltransferase deficiency"[MeSH Terms] OR ("lecithin"[All Fields] AND "cholesterol"[All Fields] AND "acyltransferase"[All Fields] AND "deficiency"[All Fields]) OR "lecithin cholesterol acyltransferase deficiency"[All Fields] OR ("acyltransferase"[All Fields] AND "deficiency"[All Fields] AND "lecithin"[All Fields] AND "cholesterol"[All Fields])) OR "alpha-Lecithin-Cholesterol Acyltransferase Deficiency"[All Fields] OR ("lecithin cholesterol acyltransferase deficiency"[MeSH Terms] OR ("lecithin"[All Fields] AND "cholesterol"[All Fields] AND "acyltransferase"[All Fields] AND "deficiency"[All Fields]) OR "lecithin cholesterol acyltransferase deficiency"[All Fields] OR ("norum"[All Fields] AND "disease"[All Fields])) OR ("lecithin cholesterol acyltransferase deficiency"[MeSH Terms] OR ("lecithin"[All Fields] AND "cholesterol"[All Fields] AND "acyltransferase"[All Fields] AND "deficiency"[All Fields]) OR "lecithin cholesterol acyltransferase deficiency"[All Fields] OR ("lcata"[All Fields] AND "deficiency"[All Fields])) OR ("lecithin cholesterol acyltransferase deficiency"[MeSH Terms] OR ("lecithin"[All Fields] AND "cholesterol"[All Fields] AND "acyltransferase"[All Fields] AND "deficiency"[All Fields]) OR "lecithin cholesterol acyltransferase deficiency"[All Fields] OR ("lcata"[All Fields] AND "deficiencies"[All Fields])) OR "alpha-Lecithin:Cholesterol Acyltransferase Deficiency"[All Fields] OR "LCAT Deficiency"[All Fields] OR ("lecithin cholesterol acyltransferase deficiency"[MeSH Terms] OR ("lecithin"[All Fields] AND "cholesterol"[All Fields] AND "acyltransferase"[All Fields] AND "deficiency"[All Fields]) OR "lecithin cholesterol acyltransferase deficiency"[All Fields] OR ("deficiency"[All Fields] AND "lcat"[All Fields])) OR "alpha-LCAT Deficiency"[All Fields] OR ("lecithin cholesterol acyltransferase deficiency"[MeSH Terms] OR ("lecithin"[All Fields] AND "cholesterol"[All Fields] AND "acyltransferase"[All Fields] AND "deficiency"[All Fields]) OR "lecithin cholesterol acyltransferase deficiency"[All Fields] OR ("deficiency"[All Fields] AND "alpha"[All Fields] AND "lcat"[All Fields])) OR "alpha LCAT Deficiency"[All Fields] OR "Fish-Eye Disease"[All Fields] OR "Fish Eye Disease"[All Fields] OR ("lecithin cholesterol acyltransferase deficiency"[MeSH Terms] OR ("lecithin"[All Fields] AND "cholesterol"[All Fields] AND "acyltransferase"[All Fields] AND "deficiency"[All Fields]) OR "lecithin cholesterol acyltransferase deficiency"[All Fields] OR ("dyslipoproteinemic"[All Fields] AND "corneal"[All Fields] AND "dystrophy"[All Fields]))) OR ("Lecithin:Cholesterol Acyltransferase Deficiency"[Other Term] OR "LCAT Deficiency"[Other Term] OR "Fish-Eye Disease"[Other Term] OR "Fish Eye Disease"[Other Term]) AND ((Case Reports[ptyp] OR Classical Article[ptyp] OR Clinical Study[ptyp] OR Clinical Trial[ptyp] OR Clinical Trial Protocol[ptyp] OR Clinical Trial, Phase I[ptyp] OR Clinical Trial, Phase II[ptyp] OR Clinical Trial, Phase III[ptyp] OR Clinical Trial, Phase IV[ptyp] OR Comparative Study[ptyp] OR Controlled Clinical Trial[ptyp] OR Corrected and Republished Article[sb] OR English Abstract[ptyp] OR Introductory Journal Article[ptyp] OR Journal Article[ptyp] OR Meta-Analysis[ptyp] OR Multicenter Study[ptyp] OR Observational Study[ptyp] OR Pragmatic Clinical Trial[ptyp] OR Published Erratum[sb] OR Randomized Controlled Trial[ptyp]) AND "loattrfull text"[sb] AND English[lang]) AND ((Case Reports[ptyp] OR Classical Article[ptyp] OR Clinical Study[ptyp] OR Clinical Trial[ptyp] OR Clinical Trial Protocol[ptyp] OR Clinical Trial, Phase I[ptyp] OR Clinical Trial, Phase II[ptyp] OR Clinical Trial, Phase III[ptyp] OR Clinical Trial, Phase IV[ptyp] OR Clinical Trial, Veterinary[ptyp] OR Comparative Study[ptyp] OR Controlled Clinical Trial[ptyp] OR Corrected and Republished Article[sb] OR Introductory Journal Article[ptyp] OR Journal Article[ptyp] OR Meta-Analysis[ptyp] OR Multicenter Study[ptyp] OR Observational Study[ptyp] OR Pragmatic Clinical Trial[ptyp] OR Published Erratum[sb] OR Randomized Controlled Trial[ptyp]) AND full text[sb] AND English[lang])

2. Data extraction.

Data were extracted according to the criteria and definitions listed below. When outcomes were determined more than once for a subject, we annotated both outcome and age at presentation. When possible, data presented in graphical form were retrieved using the WebPlotDigitizer software. Cumulative data referring to subjects with the same genetic background were included in the study and average values per group were extracted, accounting for the number of subjects within the group. For one record, primary data were provided by the author (KS)(39).

In order to limit confounding factors that could affect the accurate representation of results, eGFR collected after renal replacement therapy and data collected after transfusion or during pregnancy were not included in the analysis.

List of outcomes extracted and definitions

Data item	Definition
**Age at determination of outcome**	Age (years) was reported as indicated or determined based on the information indicated in the manuscripts (year of birth, year of occurrence of clinical trait, year or publication).<br>If repeated measures and observations were available for a singles subject, age at each outcome determination was extracted. The entries "childhood" and "adolescence/teenage" were converted into 7 and 15 y/o, respectively. The entry "puberty" was converted into 14 or 13 y/o (for males and females, respectively). These approximations were determined as the mean value of the following age ranges: childhood, 3-11 y/o, adolescence/teenage, 12-18 y/o (162), puberty, 9-14 for males and 8-13 for females (163).
**Gender**	As reported
**Region of provenience**	Region of provenience was determined based on the country of provenience or country of recruitment/description.<br>Region of provenience was categorized as follows:<br><br>Asia (Japan, Korea)<br>Australia<br>Canada/USA<br>Europe (UK, Ireland, Denmark, Netherlands, Finland, France, Belgium, Germany, Austria, Greece, Italy, Norway, Poland, Portugal, Bulgaria, Romania, Spain, Sweden)<br>Latin America (Mexico, Ecuador, Chile)<br>Middle East (Iran, Lebanon, Turkey)<br>North Africa (Morocco, Algeria)<br>South Asia (India, Pakistan)
**Plasma total cholesterol (TC)**<br>**HDL-cholesterol (HDL-C)**<br>**LDL-cholesterol (LDL-C) Triglycerides (TG)**<br>**Phospholipids (PL)**<br>**ApoA-I**<br>**ApoA-II**<br>**ApoB**	TC, LDL-C, HDL-C, ApoA-I, ApoA-II, ApoC-III, ApoC-II, ApoE and Lp(a) values were reported as mg/dL.<br>If in the source manuscript cholesterol, triglycerides and phospholipid levels were reported as mmol/L, they were converted into mg/dL by multiplying the values by 38.67, 88.57 and 30.86, respectively.

Data item	Definition
**ApoC-III**<br>**ApoE**<br>**Lp(a)**	
**Non-HDL-Cholesterol (non-HDL-C)**	Non-HDL-C was calculated as $TC_{(mg/dL)}-(HDL-C_{(mg/dL)})$.
**Unesterified cholesterol (UC)**	UC levels were reported as mg/dL.<br><br>If in the source manuscript UC levels were reported as mmol/L, they were converted into mg/dL by multiplying the values by 38.67.<br><br>If in the source manuscript UC/TC ratio and TC levels were provided, UC levels were calculated according to the following formula:<br>$UC_{(mg/dL)}=(UC/TC_{ratio})*TC_{(mg/dL)}$<br><br>If in the source manuscript EC%, and TC levels were provided, UC levels were calculated according to the following formula:<br>$UC_{(mg/dL)}= [(100-EC\%)/100]*TC_{(mg/dL)}$<br><br>If in the source manuscript UC/EC ratio and TC levels were provided, UC levels were calculated according to the following formula:<br>$UC_{(mg/dL)}=[TC_{(mg/dL)} / (1+UC/EC_{ratio})] *(UC/EC_{ratio})$
**Percentage of esterified cholesterol (EC%)**	The percentage of esterified cholesterol represents the relative amount of esterified cholesterol to total cholesterol (EC%).<br><br>If in the source manuscript EC and TC levels were provided, EC% levels were calculated according to the following formula:<br>$EC\%=(EC_{(mg/dL)}/TC_{(mg/dL)})*100$.<br><br>If in the source manuscript EC levels were reported as mmol/L, they were converted into mg/dL by multiplying the values by 38.67.<br><br>If in the source manuscript UC and TC levels were provided, EC% levels were calculated according to the following formula:<br>$EC\%=[(TC_{(mg/dL)}-UC_{(mg/dL)})/TC_{(mg/dL)}]*100$.<br><br>If in the source manuscript UC/TC ratio was provided, EC% levels were calculated according to the following formula:<br>$EC\%=[1-(UC/TC_{ratio})]*100$<br><br>If in the source manuscript UC/EC ratio was provided, EC% levels were calculated according to the following formula:<br>$EC\%=[1 / (1+UC/EC_{ratio})] *100$
**LpX**	Outcome was reported as presence or absence.
**LCAT enzymatic activity on endogenous, exogenous substrates and Plasma LCAT concentration**	We included in this analysis the determination of two types of LCAT enzymatic activity: LCAT activity on exogenous substrates (synthetic proteoliposomes and lipid substrates, α activity), and the LCAT activity on endogenous lipoproteins (whole autologous plasma, α and β activity).<br>Since the measurements of LCAT activity and concentration are highly method and laboratory-dependent, we could not provide one unique reference value for all the studies. For this reason, such parameters were normalized to the mean of the control values cited in the same paper (% of control values). Control values were defined as the mean of values determined in non-carriers of LCAT genetic variants or alternatively, the mean value of the reference range determined in non-carriers of LCAT genetic variants.<br>In order to limit method-method variability in the quantitative analysis, we did not include activity levels measured using non-autologous plasma.

Data item	Definition
**Corneal opacity**	Outcome was reported as presence or absence.
**Anemia**	Outcome was reported as presence or absence. Presence of anemia was defined based on clinical diagnosis or alternatively when hemoglobin levels were <13.5 g/dL (men) and 12.0 g/dL (women).
**Renal abnormalities**	The outcome was reported as presence or absence.<br>If the presence of renal disease or renal abnormalities was reported in the paper, we extracted this outcome as presence of renal disease.<br>We also defined as renal abnormalities the presence of proteinuria, or eGFR< 60 mL/min/1.73 m^2.
**Proteinuria**	Outcome was reported as presence or absence.
**Hematuria**	Outcome was reported as presence or absence.
**Glomerular Filtration Rate**	Glomerular Filtration Rate (GFR, mL/min/1.73 $m^{2,}$) was extracted as reported.<br>If creatinine levels, age, gender and ethnicity were reported, estimated GFR (eGFR) was calculated using the CKD-EPI creatinine equation (164, 165).<br>If renal function was reported as “normal” we estimated eGFR as 90 mL/min/1.73 m^2, based on the eGFR threshold for the definition of CKD stage 0-I.<br>If eGFR at the time of onset of ESRD was not reported, we estimated it as 14 mL/min/1.73 m^2, based on the eGFR threshold for the definition of CKD stage V.
**CKD stage**	CKD stage. Based on the eGFR value and presence or absence of proteinuria, the renal disease was graded into the following stages<br>CKD Stage — GFR, mL/min/1.73 m^2<br>0 — $\geq$90, absence of proteinuria<br>I — $\geq$90, presence of proteinuria<br>II — 60–89<br>III — 30–59<br>IV — 15–29<br>V — <15
**Presence of End-Stage Renal disease (ESRD)**	ESRD was defined as initiation of renal replacement therapy (dialysis or kidney transplant).
**Kidney Transplant**	Outcome was annotated as presence or absence
**Kidney Transplant Failure/Reoccurrence of disease**	Where available, follow up information on the outcome of kidney transplantation were recorded. Information was extracted as presence/absence of transplant failure and presence/absence recurrence of signs of renal damage.
**Histological assessment of renal damage**	Findings from kidney biopsies were reported as presence or absence of the following key histologic features:<br><br>Global and/or segmental glomerular sclerosis<br>Vacuolization of glomerular cells<br>Foam cell infiltration<br>Macrophage infiltration<br>Mesangial matrix expansion<br>Mesangial deposits<br>Vacuolization of mesangial area<br>Mesangial foam cell infiltration<br>Thickening of the GBM<br>Duplication of the GBM<br>Vacuolization of GBM

Data item	Definition
	Lipid deposits in GBM<br>Thickened capillary walls<br>Pericapillary depositions<br>Effacement of podocyte foot processes<br>Tubulo-interstitial changes<br>Tubular Atrophy<br>Interstitial Fibrosis<br>Tubular cell vacuolization<br>Thickening of TBM<br>TBM Vacuolization<br>Interstitial inflammation<br>Interstitial edema<br>Interstitial macrophages<br>Intimal thickening<br>Hyalinosis<br>Pseudo Thrombi<br>Subendothelial deposits<br>IgA (Immunofluorescence staining)<br>IgG (Immunofluorescence staining)<br>IgM (Immunofluorescence staining)<br>C3 (Immunofluorescence staining)<br>C4 (Immunofluorescence staining)<br>C1q (Immunofluorescence staining)
**Cardiovascular disease (CVD)**	CVD was categorized into three groups:<br><br>Clinically significant ASCVD (atherosclerotic CVD):<br>Myocardial infarction (MI)/unstable angina<br>Clinically significant coronary artery disease (CAD), defined as occlusions ≥ 50% based on imaging (CT angiography, catheterization) and/or intervention by PCI (stent placement, angioplasty) or bypass surgery<br>Clinically significant carotid disease, defined as occlusion ≥ 50% based on imaging and/or intervention (endarterectomy, stent, etc.)<br>Clinically significant peripheral arterial disease (PAD), defined as ankle-brachial index ABI <0.8 and/or intervention<br>Cerebrovascular accident (CVA/stroke) except for hemorrhagic stroke<br><br>Asymptomatic ASCVD:<br>CAC (coronary artery calcium) score above average/normal (>50th percentile, or as defined in the study) or >400<br>CIMT (carotid intima-media thickness) greater than average/above normal (>50th percentile, or as defined in the study).<br>Non-occlusive CAD found on imaging, e.g. CT angiography or catheterization<br><br>Other CVD<br>Valvular disease, e.g. aortic stenosis<br>Aortic aneurism<br>Heart failure<br>Hemorrhagic stroke/CVA<br>Arrhythmias
**Gene and protein Variant identification**	Genetic and protein variants were indicated according to the guidelines of Human Genome Variation Society (55). Variants were described using their location on the genomic or coding reference sequences (Reference GenBank numbers NG_009778 and NM_000229, respectively). The location of the variants in the LCAT protein was determined using the amino acid numbering that refers to the protein sequence inclusive of the 24 amino acid signal peptide (440 amino acids).
**I*n vitro* prediction of the effects of identified LCAT variants on enzymatic activity.**	In order to complement the clinical diagnosis of FLD and FED patients, we extracted data on the predicted effects of *LCAT* variants on enzymatic activity. These data were extracted from *in vitro* studies. The activities of recombinant proteins on HDL-like particles (α activity), isolated LDL particles (β activity) and plasma lipoproteins (α+β activity), were extracted and graded based on the relative activity compared to wild type protein. Approximate activity levels below 25% of WT were considered consistent with a

Data item	Definition
	complete enzymatic deficiency whereas levels above 25% were considered compatible with partial enzymatic deficiency.<br>For variants that induce premature termination of protein translation, the predicted effect was estimated to be complete enzymatic deficiency.

.

3. Synopsis of the subject classification protocol.

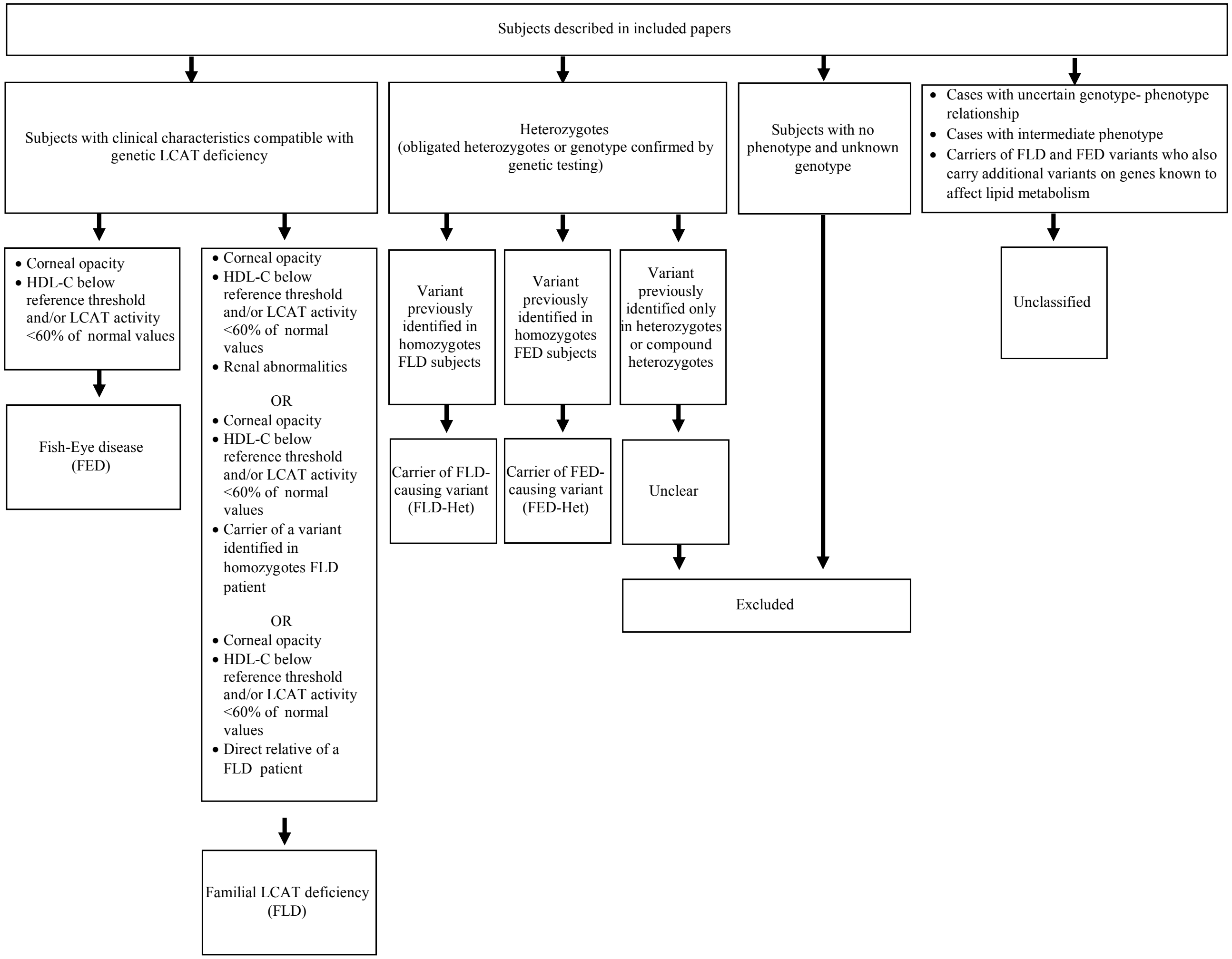

Subjects were categorized based on their clinical and genetic characteristics. For the purpose of this classification, “renal abnormalities” includes: presence of proteinuria/hematuria and/or eGFR <60 mL/min/1.73 m^2 and/or report of renal abnormalities.

5. Regression analyses.

Association of LCAT activity towards endogenous lipoproteins, exogenous substrate, UC and EC% with FLD phenotype after adjustment for LCAT concentration (Table S8).

The association was tested in FED and FLD patients. We performed a multivariate linear regression using levels of each parameter as the dependent variable and the presence of FLD phenotype (FLD) and plasma LCAT concentration (% of controls) as explanatory variables. For each subject, the levels of the parameters were determined as the average of all the available determinations.

Linear Regression of LCAT activity towards endogenous lipoproteins and LCAT activity towards exogenous substrate and EC% (Figure 2).

The association between EC% and LCAT activity measurements (LCAT activity towards endogenous lipoproteins and LCAT activity towards exogenous substrate), was assessed using logit and root square transformation, respectively followed by linear regression analysis.

For logit transformation of EC% values, an offset of 0.5 was used, determined as the minimum (non-null) observed value/2.

Multivariate Logistic Regression of severe CKD/ESRD odds in FLD subjects (Table 7).

The association between the prevalence of CKD stage III-V/ESRD and biomarkers of interest was determined by univariate and multivariate logistic regression using CKD stage III-V/ESRD as dependent variable and the levels of lipid biomarker (univariate analysis), lipid biomarker and age (model 1) or lipid biomarker, age and sex (model 2) as explanatory variables. For each subject, the presence or absence of either CKD stage III-V or ESRD was defined as composite outcome. Levels of lipid biomarkers were determined as the average of all the available determinations. For regression analysis, age refers to the age at time of first occurrence of CKD stage III-V/ESRD. Statistical significance was determined with Wald Chi-Square test.

Univariate and multivariate Linear Regression of eGFR change in FLD subjects (Table 7).

The association between annual rate of eGFR change and biomarkers of interest was determined by univariate and multivariate linear regression analysis using "eGFR change" as dependent variable and the

levels of lipid biomarker (univariate analysis), lipid biomarker and age (model 1) or lipid biomarker, age and sex (model 2) as explanatory variables. For each subject "eGFR change" was determined as the slope obtained by linear regression analysis of eGFR and age. All the eGFR assessments prior to renal replacement therapy were used. Levels of lipid biomarkers were determined as the average of all the available determinations. For regression analysis, age refers to the age at time of first eGFR determination

References (supplemental material)

1. Calabresi, L., L. Pisciotta, A. Costantin, I. Frigerio, I. Eberini, P. Alessandrini, M. Arca, G. B. Bon, G. Boscutti, G. Busnach, G. Frasca, L. Gesualdo, M. Gigante, G. Lupattelli, A. Montali, et al. (2005) The molecular basis of lecithin:cholesterol acyltransferase deficiency syndromes: a comprehensive study of molecular and biochemical findings in 13 unrelated Italian families. *Arterioscler Thromb Vasc Biol* **25**: 1972-1978.

2. Ustaoglu, M., N. Solmaz, B. Baser, H. K. Kurtulgan, and F. Onder. (2019) Ocular and Genetic Characteristics Observed in Two Cases of Fish-Eye Disease. *Cornea* **38**: 379-383.

3. Okubo, M., Y. Aoyama, H. Shio, J. J. Albers, and T. Murase. (1996) A novel missense mutation (Asn5-->Ile) in lecithin: cholesterol acyltransferase (LCAT) gene in a Japanese patient with LCAT deficiency. *Int J Clin Lab Res* **26**: 250-254.

4. Skretting, G., and H. Prydz. (1992) An amino acid exchange in exon I of the human lecithin: cholesterol acyltransferase (LCAT) gene is associated with fish eye disease. *Biochemical and biophysical research communications* **182**: 583-587.

5. Bujo, H., J. Kusunoki, M. Ogasawara, T. Yamamoto, Y. Ohta, T. Shimada, Y. Saito, and S. Yoshida. (1991) Molecular defect in familial lecithin:cholesterol acyltransferase (LCAT) deficiency: a single nucleotide insertion in LCAT gene causes a complete deficient type of the disease. *Biochemical and biophysical research communications* **181**: 933-940.

6. Baass, A., H. Wassef, M. Tremblay, L. Bernier, R. Dufour, and J. Davignon. (2009) Characterization of a new LCAT mutation causing familial LCAT deficiency (FLD) and the role of APOE as a modifier gene of the FLD phenotype. *Atherosclerosis* **207**: 452-457.

7. Miida, T., B. Zhang, K. Obayashi, U. Seino, Y. Zhu, T. Ito, Y. Nakamura, M. Okada, and K. Saku. (2004) T13M mutation of lecithin-cholesterol acyltransferase gene causes fish-eye disease. *Clinica chimica acta; international journal of clinical chemistry* **343**: 201-208.

8. Posadas-Sánchez, R., C. Posadas-Romero, W. A. Ocampo-Arcos, M. T. Villarreal-Molina, G. Vargas-Alarcón, E. Antúnez-Argüelles, E. Mendoza-Pérez, G. Cardoso-Saldaña, R. Martínez-Alvarado, A. Medina-Urrutia, and E. Jorge-Galarza. (2014) Premature and severe cardiovascular disease in a Mexican male with markedly low high-density-lipoprotein-cholesterol levels and a mutation in the lecithin:cholesterol acyltransferase gene: a family study. *Int J Mol Med* **33**: 1570-1576.

9. Ozkok, A., Y. Onal, I. Kilicaslan, S. Solakoglu, H. Bayramlar, and A. R. Odabas. (2017) The Case | Nephrotic syndrome with corneal opacities. *Kidney Int* **91**: 515-516.

10. Kuroda, M., A. G. Holleboom, E. S. Stroes, S. Asada, Y. Aoyagi, K. Kamata, S. Yamashita, S. Ishibashi, Y. Saito, and H. Bujo. (2014) Lipoprotein subfractions highly associated with renal damage in familial lecithin:cholesterol acyltransferase deficiency. *Arterioscler Thromb Vasc Biol* **34**: 1756-1762.

11. Yang, X. P., A. Inazu, A. Honjo, I. Koizumi, K. Kajinami, J. Koizumi, S. M. Marcovina, J. J. Albers, and H. Mabuchi. (1997) Catalytically inactive lecithin: cholesterol acyltransferase (LCAT) caused by a Gly 30 to Ser mutation in a family with LCAT deficiency. *J Lipid Res* **38**: 585-591.

12. Owen, J. S., H. Wiebusch, P. Cullen, G. F. Watts, V. L. Lima, H. Funke, and G. Assmann. (1996) Complete deficiency of plasma lecithin-cholesterol acyltransferase (LCAT) activity due to a novel homozygous mutation (Gly-30-Ser) in the LCAT gene. *Human mutation* **8**: 79-82.

13. Katayama, A., J. Wada, H. U. Kataoka, H. Yamasaki, S. Teshigawara, T. Terami, K. Inoue, M. Kanzaki, K. Murakami, A. Nakatsuka, H. Sugiyama, N. Koide, H. Bujo, and H. Makino. (2011) Two novel mutations of lecithin:cholesterol acyltransferase (LCAT) gene and the influence of APOE genotypes on clinical manifestations. *NDT plus* **4**: 299-302.

14. Naito, S., M. Kamata, M. Furuya, M. Hayashi, M. Kuroda, H. Bujo, and K. Kamata. (2013) Amelioration of circulating lipoprotein profile and proteinuria in a patient with LCAT deficiency due to a novel mutation (Cys74Tyr) in the lid region of LCAT under a fat-restricted diet and ARB treatment. *Atherosclerosis* **228**: 193-197.

15. Charlton-Menys, V., L. Pisciotta, P. N. Durrington, R. Neary, C. D. Short, L. Calabresi, S. Calandra, and S. Bertolini. (2007) Molecular characterization of two patients with severe LCAT deficiency.

Nephrology, dialysis, transplantation : official publication of the European Dialysis and Transplant Association - European Renal Association **22**: 2379-2382.

16. Oliaei, F., B. Batebi, R. Tabaripour, and H. Akhavan Niaki. (2018) Finding a very rare mutation in non-Caucasian LCAT patients from Southwest Asia for the first time. *Journal of cellular biochemistry*.

17. Funke, H., A. von Eckardstein, P. H. Pritchard, A. E. Hornby, H. Wiebusch, C. Motti, M. R. Hayden, C. Dachet, B. Jacotot, U. Gerdes, and et al. (1993) Genetic and phenotypic heterogeneity in familial lecithin: cholesterol acyltransferase (LCAT) deficiency. Six newly identified defective alleles further contribute to the structural heterogeneity in this disease. *The Journal of clinical investigation* **91**: 677-683.

18. Assmann, G., A. von Eckardstein, and H. Funke. (1991) Lecithin: cholesterol acyltransferase deficiency and fish-eye disease. *Current opinion in lipidology* **2**: 110-117.

19. Rial-Crestelo, D., I. Santos-Recuero, J. Julve, F. Blanco-Vaca, and M. Torralba. (2017) A novel homozygous mutation causing lecithin-cholesterol acyltransferase deficiency in a proband of Romanian origin with a record of extreme gestational hyperlipidemia. *J Clin Lipidol* **11**: 1475-1479.e1473.

20. Blanco-Vaca, F., S. J. Qu, C. Fiol, H. Z. Fan, Q. Pao, A. Marzal-Casacuberta, J. J. Albers, I. Hurtado, V. Gracia, X. Pinto, T. Marti, and H. J. Pownall. (1997) Molecular basis of fish-eye disease in a patient from Spain. Characterization of a novel mutation in the LCAT gene and lipid analysis of the cornea. *Arterioscler Thromb Vasc Biol* **17**: 1382-1391.

21. Morales, E., M. Alonso, B. Sarmiento, and M. Morales. (2018) LCAT deficiency as a cause of proteinuria and corneal opacification. *BMJ case reports* **2018**.

22. Kastelein, J. J., P. H. Pritchard, D. W. Erkelens, J. A. Kuivenhoven, J. J. Albers, and J. J. Frohlich. (1992) Familial high-density-lipoprotein deficiency causing corneal opacities (fish eye disease) in a family of Dutch descent. *Journal of internal medicine* **231**: 413-419.

23. Funke, H., A. von Eckardstein, P. H. Pritchard, J. J. Albers, J. J. Kastelein, C. Droste, and G. Assmann. (1991) A molecular defect causing fish eye disease: an amino acid exchange in lecithin-cholesterol acyltransferase (LCAT) leads to the selective loss of alpha-LCAT activity. *Proc Natl Acad Sci U S A* **88**: 4855-4859.

24. Kuivenhoven, J. A., E. J. van Voorst tot Voorst, H. Wiebusch, S. M. Marcovina, H. Funke, G. Assmann, P. H. Pritchard, and J. J. Kastelein. (1995) A unique genetic and biochemical presentation of fish-eye disease. *The Journal of clinical investigation* **96**: 2783-2791.

25. Teh, E. M., J. W. Chisholm, P. J. Dolphin, Y. Pouliquen, M. Savoldelli, J. L. de Gennes, and P. Benlian. (1999) Classical LCAT deficiency resulting from a novel homozygous dinucleotide deletion in exon 4 of the human lecithin: cholesterol acyltransferase gene causing a frameshift and stop codon at residue 144. *Atherosclerosis* **146**: 141-151.

26. Hirashio, S., K. Izumi, T. Ueno, T. Arakawa, T. Naito, T. Taguchi, and N. Yorioka. (2010) Point mutation (C to T) of the LCAT gene resulting in A140C substitution. *Journal of atherosclerosis and thrombosis* **17**: 1297-1301.

27. Steyrer, E., S. Haubenwallner, G. Horl, W. Giessauf, G. M. Kostner, and R. Zechner. (1995) A single G to A nucleotide transition in exon IV of the lecithin: cholesterol acyltransferase (LCAT) gene results in an Arg140 to His substitution and causes LCAT-deficiency. *Hum Genet* **96**: 105-109.

28. Gotoda, T., N. Yamada, T. Murase, M. Sakuma, N. Murayama, H. Shimano, K. Kozaki, J. J. Albers, Y. Yazaki, and Y. Akanuma. (1991) Differential phenotypic expression by three mutant alleles in familial lecithin:cholesterol acyltransferase deficiency. *Lancet (London, England)* **338**: 778-781.

29. Lucchi, T., L. Calabresi, A. Pinto, E. Benetti, B. Arosio, S. Simonelli, R. Ratiglia, and C. Vergani. (2012) A woman with low HDL cholesterol and corneal opacity. *Internal and emergency medicine* **7**: 533-537.

30. Taramelli, R., M. Pontoglio, G. Candiani, S. Ottolenghi, H. Dieplinger, A. Catapano, J. Albers, C. Vergani, and J. McLean. (1990) Lecithin cholesterol acyl transferase deficiency: molecular analysis of a mutated allele. *Hum Genet* **85**: 195-199.

31. Lamiquiz-Moneo, I., F. Civeira, D. Gomez-Coronado, F. Blanco-Vaca, H. M. Villafuerte-Ledesma, M. Gil, N. Amigo, R. Mateo-Gallego, and A. Cenarro. (2019) Lipid Profile Rather Than the LCAT Mutation Explains Renal Disease in Familial LCAT Deficiency. *Journal of clinical medicine* **8**.

32. Elkhalil, L., Z. Majd, R. Bakir, O. Perez-Mendez, G. Castro, P. Poulain, B. Lacroix, N. Duhal, J. C. Fruchart, and G. Luc. (1997) Fish-eye disease: structural and in vivo metabolic abnormalities of high-density lipoproteins. *Metabolism: clinical and experimental* **46**: 474-483.

33. Bender, B. U., T. Quaschning, H. P. Neumann, D. Schmidt, and A. Kraemer-Guth. (2007) A novel frameshift mutation of the lecithin:cholesterol acyltransferase (LCAT) gene associated with renal failure in familial LCAT deficiency. *Clinical chemistry and laboratory medicine* **45**: 483-486.

34. Wang, X. L., J. Osuga, F. Tazoe, K. Okada, S. Nagashima, M. Takahashi, T. Ohshiro, T. Bayasgalan, H. Yagyu, and S. Ishibashi. (2011) Molecular analysis of a novel LCAT mutation (Gly179 --> Arg) found in a patient with complete LCAT deficiency. *Journal of atherosclerosis and thrombosis* **18**: 713-719.

35. Haase, C. L., A. Tybjaerg-Hansen, A. A. Qayyum, J. Schou, B. G. Nordestgaard, and R. Frikke-Schmidt. (2012) LCAT, HDL cholesterol and ischemic cardiovascular disease: a Mendelian randomization study of HDL cholesterol in 54,500 individuals. *J Clin Endocrinol Metab* **97**: E248-256.

36. Dorval, I., P. Jezequel, C. Dubourg, B. Chauvel, P. Le Pogamp, and J. Y. Le Gall. (1994) Identification of the homozygous missense mutation in the lecithin:cholesterol-acyltransferase (LCAT) gene, causing LCAT familial deficiency in two French patients. *Atherosclerosis* **105**: 251-252.

37. Miettinen, H. E., H. Gylling, J. Tenhunen, J. Virtamo, M. Jauhiainen, J. K. Huttunen, I. Kantola, T. A. Miettinen, and K. Kontula. (1998) Molecular genetic study of Finns with hypoalphalipoproteinemia and hyperalphalipoproteinemia: a novel Gly230 Arg mutation (LCAT[Fin]) of lecithin:cholesterol acyltransferase (LCAT) accounts for 5% of cases with very low serum HDL cholesterol levels. *Arterioscler Thromb Vasc Biol* **18**: 591-598.

38. Castro-Ferreira, I., R. Carmo, S. E. Silva, O. Correa, S. Fernandes, S. Sampaio, R. P. Pedro, A. Praca, and J. P. Oliveira. (2018) Novel Missense LCAT Gene Mutation Associated with an Atypical Phenotype of Familial LCAT Deficiency in Two Portuguese Brothers. *JIMD reports* **40**: 55-62.

39. Fountoulakis, N., E. Lioudaki, D. Lygerou, E. K. Dermitzaki, I. Papakitsou, V. Kounali, A. G. Holleboom, S. Stratigis, C. Belogianni, P. Syngelaki, S. Stratakis, A. Evangeliou, H. Gakiopoulou, J. A.

Kuivenhoven, R. Wevers, et al. (2019) The P274S Mutation of Lecithin-Cholesterol Acyltransferase (LCAT) and Its Clinical Manifestations in a Large Kindred. *American journal of kidney diseases : the official journal of the National Kidney Foundation* **74**: 510-522.

40. Jimi, S., N. Uesugi, K. Saku, H. Itabe, B. Zhang, K. Arakawa, and S. Takebayashi. (1999) Possible induction of renal dysfunction in patients with lecithin:cholesterol acyltransferase deficiency by oxidized phosphatidylcholine in glomeruli. *Arterioscler Thromb Vasc Biol* **19**: 794-801.

41. Skretting, G., J. P. Blomhoff, J. Solheim, and H. Prydz. (1992) The genetic defect of the original Norwegian lecithin:cholesterol acyltransferase deficiency families. *FEBS letters* **309**: 307-310.

42. Moriyama, K., J. Sasaki, F. Arakawa, N. Takami, E. Maeda, A. Matsunaga, Y. Takada, K. Midorikawa, T. Yanase, G. Yoshino, and et al. (1995) Two novel point mutations in the lecithin:cholesterol acyltransferase (LCAT) gene resulting in LCAT deficiency: LCAT (G873 deletion) and LCAT (Gly344-->Ser). *J Lipid Res* **36**: 2329-2343.

43. Geller, A. S., E. Y. Polisecki, M. R. Diffenderfer, B. F. Asztalos, S. K. Karathanasis, R. A. Hegele, and E. J. Schaefer. (2018) Genetic and secondary causes of severe HDL deficiency and cardiovascular disease. *J Lipid Res* **59**: 2421-2435.

44. Roshan, B., O. P. Ganda, R. Desilva, R. B. Ganim, E. Ward, S. D. Haessler, E. Y. Polisecki, B. F. Asztalos, and E. J. Schaefer. (2011) Homozygous lecithin:cholesterol acyltransferase (LCAT) deficiency due to a new loss of function mutation and review of the literature. *J Clin Lipidol* **5**: 493-499.

45. Shamburek, R. D., R. Bakker-Arkema, B. J. Auerbach, B. R. Krause, R. Homan, M. J. Amar, L. A. Freeman, and A. T. Remaley. (2016) Familial lecithin:cholesterol acyltransferase deficiency: First-in-human treatment with enzyme replacement. *J Clin Lipidol* **10**: 356-367.

46. Maeda, E., Y. Naka, T. Matozaki, M. Sakuma, Y. Akanuma, G. Yoshino, and M. Kasuga. (1991) Lecithin-cholesterol acyltransferase (LCAT) deficiency with a missense mutation in exon 6 of the LCAT gene. *Biochemical and biophysical research communications* **178**: 460-466.

47. Rader, D. J., K. Ikewaki, N. Duverger, H. Schmidt, H. Pritchard, J. Frohlich, M. Clerc, M. F. Dumon, T. Fairwell, L. Zech, and et al. (1994) Markedly accelerated catabolism of apolipoprotein A-II

(ApoA-II) and high density lipoproteins containing ApoA-II in classic lecithin: cholesterol acyltransferase deficiency and fish-eye disease. *The Journal of clinical investigation* **93**: 321-330.

48. Klein, H. G., S. Santamarina-Fojo, N. Duverger, M. Clerc, M. F. Dumon, J. J. Albers, S. Marcovina, and H. B. Brewer, Jr. (1993) Fish eye syndrome: a molecular defect in the lecithin-cholesterol acyltransferase (LCAT) gene associated with normal alpha-LCAT-specific activity. Implications for classification and prognosis. *The Journal of clinical investigation* **92**: 479-485.

49. Idzior-Walus, B., J. Sieradzki, G. Kostner, M. T. Malecki, T. Klupa, T. Wesolowska, W. Rostworowski, J. Hartwich, M. Walus, A. D. Kiec, and M. Naruszewicz. (2006) Familial lecithin-cholesterol acyltransferase deficiency: biochemical characteristics and molecular analysis of a new LCAT mutation in a Polish family. *Atherosclerosis* **185**: 413-420.

50. Miarka, P., B. Idzior-Walus, M. Kuzniewski, M. Walus-Miarka, T. Klupa, and W. Sulowicz. (2011) Corticosteroid treatment of kidney disease in a patient with familial lecithin-cholesterol acyltransferase deficiency. *Clinical and experimental nephrology* **15**: 424-429.

51. Holleboom, A. G., J. A. Kuivenhoven, C. C. van Olden, J. Peter, A. W. Schimmel, J. H. Levels, R. M. Valentijn, P. Vos, J. C. Defesche, J. J. Kastelein, G. K. Hovingh, E. S. Stroes, and C. E. Hollak. (2011) Proteinuria in early childhood due to familial LCAT deficiency caused by loss of a disulfide bond in lecithin:cholesterol acyl transferase. *Atherosclerosis* **216**: 161-165.

52. Hirano, K., S. Kachi, C. Ushida, and M. Naito. (2004) Corneal and macular manifestations in a case of deficient lecithin: cholesterol acyltransferase. *Japanese journal of ophthalmology* **48**: 82-84.

53. Stoekenbroek, R. M., M. A. van den Bergh Weerman, G. K. Hovingh, B. J. Potter van Loon, C. E. Siegert, and A. G. Holleboom. (2013) Familial LCAT deficiency: from renal replacement to enzyme replacement. *The Netherlands journal of medicine* **71**: 29-31.

54. Gigante, M., E. Ranieri, G. Cerullo, L. Calabresi, A. Iolascon, G. Assmann, L. Morrone, L. Pisciotta, F. P. Schena, and L. Gesualdo. (2006) LCAT deficiency: molecular and phenotypic characterization of an Italian family. *J Nephrol* **19**: 375-381.

55. den Dunnen, J. T., R. Dalgleish, D. R. Maglott, R. K. Hart, M. S. Greenblatt, J. McGowan-Jordan, A. F. Roux, T. Smith, S. E. Antonarakis, and P. E. Taschner. (2016) HGVS Recommendations for the Description of Sequence Variants: 2016 Update. *Human mutation* **37**: 564-569.

56. Wiebusch, H., P. Cullen, J. S. Owen, D. Collins, P. S. Sharp, H. Funke, and G. Assmann. (1995) Deficiency of lecithin:cholesterol acyltransferase due to compound heterozygosity of two novel mutations (Gly33Arg and 30 bp ins) in the LCAT gene. *Hum Mol Genet* **4**: 143-145.

57. Miettinen, H., H. Gylling, I. Ulmanen, T. A. Miettinen, and K. Kontula. (1995) Two different allelic mutations in a Finnish family with lecithin:cholesterol acyltransferase deficiency. *Arterioscler Thromb Vasc Biol* **15**: 460-467.

58. Gylling, H., and T. A. Miettinen. (1992) Non-cholesterol sterols, absorption and synthesis of cholesterol and apolipoprotein A-I kinetics in a Finnish lecithin-cholesterol acyltransferase deficient family. *Atherosclerosis* **95**: 25-33.

59. Kuivenhoven, J. A., A. F. Stalenhoef, J. S. Hill, P. N. Demacker, A. Errami, J. J. Kastelein, and P. H. Pritchard. (1996) Two novel molecular defects in the LCAT gene are associated with fish eye disease. *Arterioscler Thromb Vasc Biol* **16**: 294-303.

60. Frasca, G. M., L. Soverini, E. Tampieri, G. Franceschini, L. Calabresi, L. Pisciotta, P. Preda, A. Vangelista, S. Stefoni, and S. Bertolini. (2004) A 33-year-old man with nephrotic syndrome and lecithin-cholesterol acyltransferase (LCAT) deficiency. Description of two new mutations in the LCAT gene. *Nephrology, dialysis, transplantation : official publication of the European Dialysis and Transplant Association - European Renal Association* **19**: 1622-1624.

61. Weber, C. L., J. Frohlich, J. Wang, R. A. Hegele, and C. Chan-Yan. (2007) Stability of lipids on peritoneal dialysis in a patient with familial LCAT deficiency. *Nephrology, dialysis, transplantation : official publication of the European Dialysis and Transplant Association - European Renal Association* **22**: 2084-2088.

62. Kettritz, R., S. Elitok, M. L. Koepke, J. Kuchenbecker, W. Schneider, and F. C. Luft. (2009) The case: the eyes have it! *Kidney Int* **76**: 465-466.

63. Horl, G., P. M. Kroisel, E. Wagner, B. Tiran, E. Petek, and E. Steyrer. (2006) Compound heterozygosity (G71R/R140H) in the lecithin:cholesterol acyltransferase (LCAT) gene results in an intermediate phenotype between LCAT-deficiency and fish-eye disease. *Atherosclerosis* **187**: 101-109.

64. Cirera, S., J. Julve, I. Ferrer, C. Mainou, R. Bonet, J. M. Martin-Campos, F. Gonzalez-Sastre, and F. Blanco-Vaca. (1998) Molecular diagnosis of lecithin: cholesterol acyltransferase deficiency in a presymptomatic proband. *Clinical chemistry and laboratory medicine* **36**: 443-448.

65. Holleboom, A. G., J. A. Kuivenhoven, F. Peelman, A. W. Schimmel, J. Peter, J. C. Defesche, J. J. Kastelein, G. K. Hovingh, E. S. Stroes, and M. M. Motazacker. (2011) High prevalence of mutations in LCAT in patients with low HDL cholesterol levels in The Netherlands: identification and characterization of eight novel mutations. *Human mutation* **32**: 1290-1298.

66. Aranda, P., P. Valdivielso, L. Pisciotta, I. Garcia, A. A. C. Garcã, S. Bertolini, N. R. G. Martã, L.-S. Gonzã, and S. Calandra. (2008) Therapeutic management of a new case of LCAT deficiency with a multifactorial long-term approach based on high doses of angiotensin II receptor blockers (ARBs). *Clinical nephrology* **69**: 213-218.

67. Contacos, C., D. R. Sullivan, K. A. Rye, H. Funke, and G. Assmann. (1996) A new molecular defect in the lecithin: cholesterol acyltransferase (LCAT) gene associated with fish eye disease. *J Lipid Res* **37**: 35-44.

68. Kuivenhoven, J. A., H. Weibusch, P. H. Pritchard, H. Funke, R. Benne, G. Assmann, and J. J. Kastelein. (1996) An intronic mutation in a lariat branchpoint sequence is a direct cause of an inherited human disorder (fish-eye disease). *The Journal of clinical investigation* **98**: 358-364.

69. Klein, H. G., P. Lohse, N. Duverger, J. J. Albers, D. J. Rader, L. A. Zech, S. Santamarina-Fojo, and H. B. Brewer, Jr. (1993) Two different allelic mutations in the lecithin:cholesterol acyltransferase (LCAT) gene resulting in classic LCAT deficiency: LCAT (tyr83-->stop) and LCAT (tyr156-->asn). *J Lipid Res* **34**: 49-58.

70. Miller, M., K. Zeller, P. C. Kwiterovich, J. J. Albers, and G. Feulner. (1995) Lecithin: cholesterol acyltransferase deficiency: identification of two defective alleles in fibroblast cDNA. *J Lipid Res* **36**: 931-938.

71. Ahmad, S. B., M. Miller, S. Hanish, S. T. Bartlett, W. Hutson, R. N. Barth, and J. C. LaMattina. (2016) Sequential kidney-liver transplantation from the same living donor for lecithin cholesterol acyl transferase deficiency. *Clinical transplantation* **30**: 1370-1374.

72. Guerin, M., C. Dachet, S. Goulinet, D. Chevet, P. J. Dolphin, M. J. Chapman, and M. Rouis. (1997) Familial lecithin:cholesterol acyltransferase deficiency: molecular analysis of a compound heterozygote: LCAT (Arg147 --> Trp) and LCAT (Tyr171 --> Stop). *Atherosclerosis* **131**: 85-95.

73. Park, C. W., M. H. Lim, D. Y. Youn, S. E. Jung, S. Chung, Y. S. Ahn, Y. S. Chang, and J. H. Lee. (2009) Two novel frame shift mutations in lecithin:cholesterol acyltransferase (LCAT) gene associated with a familial LCAT deficiency phenotype. *Atherosclerosis* **206**: 346-348.

74. Nanjee, M. N., J. Stocks, C. J. Cooke, H. O. Molhuizen, S. Marcovina, D. Crook, J. P. Kastelein, and N. E. Miller. (2003) A novel LCAT mutation (Phe382-->Val) in a kindred with familial LCAT deficiency and defective apolipoprotein B-100. *Atherosclerosis* **170**: 105-113.

75. Strom, E. H., S. Sund, M. Reier-Nilsen, C. Dorje, and T. P. Leren. (2011) Lecithin: Cholesterol Acyltransferase (LCAT) Deficiency: renal lesions with early graft recurrence. *Ultrastruct Pathol* **35**: 139-145.

76. Sessa, A., G. Battini, M. Meroni, G. Daidone, I. Carnera, P. L. Brambilla, G. Vigano, F. Giordano, F. Pallotti, L. Torri Tarelli, L. Calabresi, M. Rolleri, and S. Bertolini. (2001) Hypocomplementemic type II membranoproliferative glomerulonephritis in a male patient with familial lecithin-cholesterol acyltransferase deficiency due to two different allelic mutations. *Nephron* **88**: 268-272.

77. Argyropoulos, G., A. Jenkins, R. L. Klein, T. Lyons, B. Wagenhorst, J. St Armand, S. M. Marcovina, J. J. Albers, P. H. Pritchard, and W. T. Garvey. (1998) Transmission of two novel mutations in a pedigree with familial lecithin:cholesterol acyltransferase deficiency: structure-function relationships and studies in a compound heterozygous proband. *J Lipid Res* **39**: 1870-1876.

78. Klein, H. G., P. Lohse, P. H. Pritchard, D. Bojanovski, H. Schmidt, and H. B. Brewer, Jr. (1992) Two different allelic mutations in the lecithin-cholesterol acyltransferase gene associated with the fish eye syndrome. Lecithin-cholesterol acyltransferase (Thr123----Ile) and lecithin-cholesterol acyltransferase (Thr347----Met). *The Journal of clinical investigation* **89**: 499-506.

79. Tobar, H. E., L. R. Cataldo, T. Gonzalez, R. Rodriguez, V. Serrano, A. Arteaga, A. Alvarez-Mercado, C. F. Lagos, L. Vicuna, J. P. Miranda, A. Pereira, C. Bravo, C. M. Aguilera, S. Eyheramendy, R. Uauy, et al. (2019) Identification and functional analysis of missense mutations in the lecithin cholesterol acyltransferase gene in a Chilean patient with hypoalphalipoproteinemia. *Lipids in health and disease* **18**: 132.

80. Kasid, A., J. Rhyne, K. Zeller, H. Pritchard, and M. Miller. (2001) A novel TC deletion resulting in Pro(260)-->Stop in the human LCAT gene is associated with a dominant effect on HDL-cholesterol. *Atherosclerosis* **156**: 127-132.

81. Conca, P., S. Pileggi, S. Simonelli, E. Boer, G. Boscutti, L. Magnolo, P. Tarugi, S. Penco, G. Franceschini, L. Calabresi, and M. Gomaraschi. (2012) Novel missense variants in LCAT and APOB genes in an Italian kindred with familial lecithin:cholesterol acyltransferase deficiency and hypobetalipoproteinemia. *J Clin Lipidol* **6**: 244-250.

82. Shoji, K., H. Morita, Y. Ishigaki, C. J. Rivard, M. Takayasu, K. Nakayama, T. Nakayama, Y. Inoue, M. Ayaki, and A. Yoshimura. (2011) Lecithin-cholesterol acyltransferase (LCAT) deficiency without mutations in the coding sequence: a case report and literature review. *Clinical nephrology* **76**: 323-328.

83. Winder, A. F., J. S. Owen, P. H. Pritchard, D. Lloyd-Jones, D. T. Vallance, P. White, and R. Wray. (1999) A first British case of fish-eye disease presenting at age 75 years: a double heterozygote for defined and new mutations affecting LCAT structure and expression. *Journal of clinical pathology* **52**: 228-230.

84. Takata, K., G. Kajiyama, I. Horiuchi, T. Watanabe, H. Tokumo, and Y. Hirata. (1989) A new case of familial lecithin: cholesterol acyltransferase (LCAT) deficiency--paradoxical findings regarding LCAT mass and activity in 23 members of a family. *Japanese journal of medicine* **28**: 765-771.

85. Balwani, M. R., V. A. Ghodela, V. B. Kute, P. R. Shah, H. V. Patel, D. N. Gera, A. Vanikar, and H. L. Trivedi. (2017) An unusual presentation of LCAT deficiency as nephrotic syndrome with normal serum HDL-C level. *Journal of nephropharmacology* **6**: 23-26.

86. Pisciotta, L., L. Calabresi, G. Lupattelli, D. Siepi, M. R. Mannarino, E. Moleri, A. Bellocchio, A. Cantafora, P. Tarugi, S. Calandra, and S. Bertolini. (2005) Combined monogenic hypercholesterolemia and hypoalphalipoproteinemia caused by mutations in LDL-R and LCAT genes. *Atherosclerosis* **182**: 153-159.

87. Scarpioni, R., C. Paties, and G. Bergonzi. (2008) Dramatic atherosclerotic vascular burden in a patient with familial lecithin-cholesterol acyltransferase (LCAT) deficiency. *Nephrology, dialysis, transplantation : official publication of the European Dialysis and Transplant Association - European Renal Association* **23**: 1074;-author reply 1074-1075.

88. Borysiewicz, L. K., A. K. Soutar, D. J. Evans, G. R. Thompson, and A. J. Rees. (1982) Renal failure in familial lecithin: cholesterol acyltransferase deficiency. *The Quarterly journal of medicine* **51**: 411-426.

89. Gjone, E. (1981) Familial lecithin:cholesterol acyltransferase deficiency - a new metabolic disease with renal involvement. *Advances in nephrology from the Necker Hospital* **10**: 167-185.

90. Muthusethupathi, M. A., R. Padmanabhan, A. Date, M. Jayakumar, S. Rajendran, and R. Vijayakumar. (1999) Familial Lecithin:cholesterol acyltransferase deficiency with renal failure in two siblings. First case report from India. *Nephron* **81**: 89-93.

91. Ayyobi, A. F., S. H. McGladdery, S. Chan, G. B. John Mancini, J. S. Hill, and J. J. Frohlich. (2004) Lecithin: cholesterol acyltransferase (LCAT) deficiency and risk of vascular disease: 25 year follow-up. *Atherosclerosis* **177**: 361-366.

92. Horina, J. H., G. Wirnsberger, S. Horn, J. M. Roob, M. Ratschek, H. Holzer, H. Pogglitsch, and G. J. Krejs. (1993) Long-term follow-up of a patient with lecithin cholesterol acyltransferase deficiency syndrome after kidney transplantation. *Transplantation* **56**: 233-236.

93. Stokke, K. T., K. S. Bjerve, J. P. Blomhoff, B. Oystese, A. Flatmark, K. R. Norum, and E. Gjone. (1974) Familial lecithin:cholesterol acyltransferase deficiency. Studies on lipid composition and morphology of tissues. *Scand J Clin Lab Invest Suppl* **137**: 93-100.

94. Flatmark, A. L., T. Hovig, E. Myhre, and E. Gjone. (1977) Renal transplantation in patients with familial lecithin: cholesterol-acetyltransferase deficiency. *Transplant Proc* **9**: 1665-1671.

95. Liew, H., I. Simpson, J. Kanellis, and W. R. Mulley. (2016) Recurrent glomerulopathy in a renal allograft due to lecithin cholesterol acyltransferase deficiency. *Nephrology (Carlton)* **21**: 73-74.

96. Stoynovska, B. B. (1992) Successful renal transplantation in a patient with familial lecithin:cholesterol acyltransferase deficiency. *Transpl Int* **5**: 187.

97. Panescu, V., Y. Grignon, D. Hestin, G. Rostoker, L. Frimat, E. Renoult, J. Gamberoni, G. Grignon, and M. Kessler. (1997) Recurrence of lecithin cholesterol acyltransferase deficiency after kidney transplantation. *Nephrology, dialysis, transplantation : official publication of the European Dialysis and Transplant Association - European Renal Association* **12**: 2430-2432.

98. Tsuchiya, Y., Y. Ubara, R. Hiramatsu, T. Suwabe, J. Hoshino, K. Sumida, E. Hasegawa, M. Yamanouchi, N. Hayami, Y. Marui, N. Sawa, S. Hara, K. Takaichi, and K. Oohashi. (2011) A case of familial lecithin-cholesterol acyltransferase deficiency on hemodialysis for over 20 years. *Clinical nephrology* **76**: 492-498.

99. Iwamoto, A., C. Naito, T. Teramoto, H. Kato, M. Kako, T. Kariya, T. Shimizu, H. Oka, and T. Oda. (1978) Familial lecithin: cholesterol acyltransferase deficiency complicated with unconjugated hyperbilirubinemia and peripheral neuropathy. The first reported cases in the Far East. *Acta Med Scand* **204**: 219-227.

100. Lager, D. J., B. F. Rosenberg, H. Shapiro, and J. Bernstein. (1991) Lecithin cholesterol acyltransferase deficiency: ultrastructural examination of sequential renal biopsies. *Modern pathology : an official journal of the United States and Canadian Academy of Pathology, Inc* **4**: 331-335.

101. Homma, S., N. Murayama, I. Yoshida, E. Kusano, K. Kuriki, K. Saito, and Y. Asano. (2001) Marked atherosclerosis in a patient with familiar lecithin: cholesterol acyltransferase deficiency associated with end-stage renal disease and diabetes mellitus. *American journal of nephrology* **21**: 415-419.

102. Ohta, Y., S. Yamamoto, H. Tsuchida, S. Murano, Y. Saitoh, S. Tohjo, and M. Okada. (1986) Nephropathy of familial lecithin-cholesterol acyltransferase deficiency: report of a case. *American journal of kidney diseases : the official journal of the National Kidney Foundation* **7**: 41-46.

103. Sahay, M., P. S. Vali, K. Ismal, S. Gowrishankar, M. D. Padua, and M. Swain. (2016) An unusual case of nephrotic syndrome. *Indian journal of nephrology* **26**: 55-56.

104. Imbasciati, E., C. Paties, L. Scarpioni, and M. J. Mihatsch. (1986) Renal lesions in familial lecithin-cholesterol acyltransferase deficiency. Ultrastructural heterogeneity of glomerular changes. *American journal of nephrology* **6**: 66-70.

105. Magil, A., W. Chase, and J. Frohlich. (1982) Unusual renal biopsy findings in a patient with familial lecithin:cholesterol acyltransferase deficiency. *Human pathology* **13**: 283-285.

106. Frohlich, J., K. Hon, and R. McLeod. (1982) Detection of heterozygotes for familial lecithin: cholesterol acyltransferase (LCAT) deficiency. *Am J Hum Genet* **34**: 65-72.

107. Naghashpour, M., and H. Cualing. (2009) Splenomegaly with sea-blue histiocytosis, dyslipidemia, and nephropathy in a patient with lecithin-cholesterol acyltransferase deficiency: a clinicopathologic correlation. *Metabolism: clinical and experimental* **58**: 1459-1464.

108. Jahanzad, I., S. Amoueian, and A. Attaranzadeh. (2009) Familial lecithin-cholesterol acyltransferase deficiency. *Archives of Iranian medicine* **12**: 179-181.

109. Neumann, M. J., and S. Whisiker-Lewis. (1994) Lecithin-cholesterol acyltransferase deficiency: first report of case in a United States citizen. *The Journal of the American Osteopathic Association* **94**: 333-335.

110. Guerin, M., P. J. Dolphin, and M. J. Chapman. (1993) Familial lecithin:cholesterol acyltransferase deficiency: further resolution of lipoprotein particle heterogeneity in the low density interval. *Atherosclerosis* **104**: 195-212.

111. Gjone, E., and K. R. Norum. (1968) Familial serum cholesterol ester deficiency. Clinical study of a patient with a new syndrome. *Acta Med Scand* **183**: 107-112.

112. Myhre, E., E. Gjone, A. Flatmark, and T. Hovig. (1977) Renal failure in familial lecithin-cholesterol acyltransferase deficiency. *Nephron* **18**: 239-248.

113. Gjone, E. (1974) Familial lecithin:cholesterol acyltransferase deficiency--a clinical survey. *Scand J Clin Lab Invest Suppl* **137**: 73-82.

114. Gjone, E., J. P. Blomhoff, R. Holme, T. Hovig, B. Olaisen, A. J. Skarbovik, and P. Teisberg. (1981) Familial lecithin:cholesterol acyltransferase deficiency. Report of a fourth family from northwestern Norway. *Acta Med Scand* **210**: 3-6.

115. Hovig, T., and E. Gjone. (1973) Familial plasma lecithin: cholesterol acyltransferase (LCAT) deficiency. Ultrastructural aspects of a new syndrome with particular reference to lesions in the kidneys and the spleen. *Acta pathologica et microbiologica Scandinavica. Section A, Pathology* **81**: 681-697.

116. Mahapatra, H. S., S. Ramanarayanan, A. Gupta, and M. Bhardwaj. (2015) Co-existence of classic familial lecithin-cholesterol acyl transferase deficiency and fish eye disease in the same family. *Indian journal of nephrology* **25**: 362-365.

117. Althaf, M. M., H. Almana, A. Abdelfadiel, S. M. Amer, and T. O. Al-Hussain. (2015) Familial lecithin-cholesterol acyltransferase (LCAT) deficiency; a differential of proteinuria. *Journal of nephropathology* **4**: 25-28.

118. Rajpal, J. S., J. Mapel-Lentz, A. D. Mancera, R. C. Reed, Y. Kim, and B. M. Chavers. (2014) Familial LCAT deficiency in a child with nephrotic syndrome. *Clinical nephrology* **82**: 211-214.

119. Weber, P., J. S. Owen, K. Desai, and M. R. Clemens. (1987) Hereditary lecithin-cholesterol acyltransferase deficiency. Case report of a German patient. *Am J Clin Pathol* **88**: 510-516.

120. Kaur, A., and S. Sethi. (2016) Histiocytic and Nonhistiocytic Glomerular Lesions: Foam Cells and Their Mimickers. *American journal of kidney diseases : the official journal of the National Kidney Foundation* **67**: 329-336.

121. Chevet, D., M. P. Ramée, P. Le Pogamp, R. Thomas, M. Garré, and L. G. Alcindor. (1978) Hereditary lecithin cholesterol acyltransferase deficiency. Report of a new family with two afflicted sisters. *Nephron* **20**: 212-219.

122. Hovingh, G. K., B. A. Hutten, A. G. Holleboom, W. Petersen, P. Rol, A. Stalenhoef, A. H. Zwinderman, E. de Groot, J. J. Kastelein, and J. A. Kuivenhoven. (2005) Compromised LCAT function is associated with increased atherosclerosis. *Circulation* **112**: 879-884.

123. Calabresi, L., D. Baldassarre, S. Castelnuovo, P. Conca, L. Bocchi, C. Candini, B. Frigerio, M. Amato, C. R. Sirtori, P. Alessandrini, M. Arca, G. Boscutti, L. Cattin, L. Gesualdo, T. Sampietro, et al. (2009) Functional lecithin: cholesterol acyltransferase is not required for efficient atheroprotection in humans. *Circulation* **120**: 628-635.

124. Calabresi, L., E. Favari, E. Moleri, M. P. Adorni, M. Pedrelli, S. Costa, W. Jessup, I. C. Gelissen, P. T. Kovanen, F. Bernini, and G. Franceschini. (2009) Functional LCAT is not required for macrophage cholesterol efflux to human serum. *Atherosclerosis* **204**: 141-146.

125. Duivenvoorden, R., A. G. Holleboom, B. van den Bogaard, A. J. Nederveen, E. de Groot, B. A. Hutten, A. W. Schimmel, G. K. Hovingh, J. J. Kastelein, J. A. Kuivenhoven, and E. S. Stroes. (2011) Carriers of lecithin cholesterol acyltransferase gene mutations have accelerated atherogenesis as assessed by carotid 3.0-T magnetic resonance imaging [corrected]. *Journal of the American College of Cardiology* **58**: 2481-2487.

126. van den Bogaard, B., A. G. Holleboom, R. Duivenvoorden, B. A. Hutten, J. J. Kastelein, G. K. Hovingh, J. A. Kuivenhoven, E. S. Stroes, and B. J. van den Born. (2012) Patients with low HDL-cholesterol caused by mutations in LCAT have increased arterial stiffness. *Atherosclerosis* **225**: 481-485.

127. Tietjen, I., G. K. Hovingh, R. Singaraja, C. Radomski, J. McEwen, E. Chan, M. Mattice, A. Legendre, J. J. Kastelein, and M. R. Hayden. (2012) Increased risk of coronary artery disease in Caucasians with extremely low HDL cholesterol due to mutations in ABCA1, APOA1, and LCAT. *Biochimica et biophysica acta* **1821**: 416-424.

128. Gomaraschi, M., A. Ossoli, S. Castelnuovo, S. Simonelli, C. Pavanello, G. Balzarotti, M. Arca, A. Di Costanzo, T. Sampietro, G. Vaudo, D. Baldassarre, F. Veglia, G. Franceschini, and L. Calabresi. (2017) Depletion in LpA-I:A-II particles enhances HDL-mediated endothelial protection in familial LCAT deficiency. *J Lipid Res* **58**: 994-1001.

129. Oldoni, F., D. Baldassarre, S. Castelnuovo, A. Ossoli, M. Amato, J. van Capelleveen, G. K. Hovingh, E. De Groot, A. Bochem, S. Simonelli, S. Barbieri, F. Veglia, G. Franceschini, J. A. Kuivenhoven, A. G. Holleboom, et al. (2018) Complete and Partial Lecithin:Cholesterol Acyltransferase Deficiency Is Differentially Associated With Atherosclerosis. *Circulation* **138**: 1000-1007.

130. Klein, H. G., N. Duverger, J. J. Albers, S. Marcovina, H. B. Brewer, Jr., and S. Santamarina-Fojo. (1995) In vitro expression of structural defects in the lecithin-cholesterol acyltransferase gene. *The Journal of biological chemistry* **270**: 9443-9447.

131. Qu, S. J., H. Z. Fan, F. Blanco-Vaca, and H. J. Pownall. (1995) In vitro expression of natural mutants of human lecithin:cholesterol acyltransferase. *J Lipid Res* **36**: 967-974.

132. Carlson, L. A. (1982) Fish eye disease: a new familial condition with massive corneal opacities and dyslipoproteinaemia. *Eur J Clin Invest* **12**: 41-53.

133. Carlson, L. A., and L. Holmquist. (1985) Paradoxical esterification of plasma cholesterol in fish eye disease. *Acta Med Scand* **217**: 491-499.

134. Murano, S., K. Shirai, Y. Saito, S. Yoshida, Y. Ohta, H. Tsuchida, S. Yamamoto, G. Asano, C. H. Chen, and J. J. Albers. (1987) Impaired intermediate-density lipoprotein triglyceride hydrolysis in familial lecithin:cholesterol acyltransferase (LCAT) deficiency. *Scandinavian journal of clinical and laboratory investigation* **47**: 775-783.

135. Ohta, T., S. Hattori, R. Nakamura, S. Horiuchi, J. Frohlich, K. Takata, Y. Ikeda, Y. Saito, and I. Matsuda. (1994) Characterization of subspecies of apolipoprotein A-I-containing lipoprotein in homozygotes for familial lecithin:cholesterol acyltransferase deficiency. *Arteriosclerosis and thrombosis : a journal of vascular biology* **14**: 1137-1145.

136. Peelman, F., J. L. Verschelde, B. Vanloo, C. Ampe, C. Labeur, J. Tavernier, J. Vandekerckhove, and M. Rosseneu. (1999) Effects of natural mutations in lecithin:cholesterol acyltransferase on the enzyme structure and activity. *J Lipid Res* **40**: 59-69.

137. Hill, J. S., K. O, X. Wang, and P. H. Pritchard. (1993) Lecithin:cholesterol acyltransferase deficiency: identification of a causative gene mutation and a co-inherited protein polymorphism. *Biochimica et biophysica acta* **1181**: 321-323.

138. Adimoolam, S., L. Jin, E. Grabbe, J. J. Shieh, and A. Jonas. (1998) Structural and functional properties of two mutants of lecithin-cholesterol acyltransferase (T123I and N228K). *The Journal of biological chemistry* **273**: 32561-32567.

139. Fotakis, P., J. A. Kuivenhoven, E. Dafnis, D. Kardassis, and V. I. Zannis. (2015) The Effect of Natural LCAT Mutations on the Biogenesis of HDL. *Biochemistry* **54**: 3348-3359.

140. Vanloo, B., F. Peelman, K. Deschuymere, J. Taveirne, A. Verhee, C. Gouyette, C. Labeur, J. Vandekerckhove, J. Tavernier, and M. Rosseneu. (2000) Relationship between structure and biochemical phenotype of lecithin:cholesterol acyltransferase (LCAT) mutants causing fish-eye disease. *J Lipid Res* **41**: 752-761.

141. O, K., J. S. Hill, X. Wang, and P. H. Pritchard. (1993) Recombinant lecithin:cholesterol acyltransferase containing a Thr123-->Ile mutation esterifies cholesterol in low density lipoprotein but not in high density lipoprotein. *J Lipid Res* **34**: 81-88.

142. Manthei, K. A., J. Ahn, A. Glukhova, W. Yuan, C. Larkin, T. D. Manett, L. Chang, J. A. Shayman, M. J. Axley, A. Schwendeman, and J. J. G. Tesmer. (2017) A retractable lid in lecithin:cholesterol acyltransferase provides a structural mechanism for activation by apolipoprotein A-I. *The Journal of biological chemistry* **292**: 20313-20327.

143. Steyrer, E., S. Durovic, S. Frank, W. Giessauf, A. Burger, H. Dieplinger, R. Zechner, and G. M. Kostner. (1994) The role of lecithin: cholesterol acyltransferase for lipoprotein (a) assembly. Structural integrity of low density lipoproteins is a prerequisite for Lp(a) formation in human plasma. *The Journal of clinical investigation* **94**: 2330-2340.

144. Vergani, C., A. L. Catapano, P. Roma, and G. Giudici. (1983) A new case of familial LCAT deficiency. *Acta Med Scand* **214**: 173-176.

145. Albers, J. J., J. Adolphson, C. H. Chen, N. Murayama, S. Honma, and Y. Akanuma. (1985) Defective enzyme causes lecithin-cholesterol acyltransferase deficiency in a Japanese kindred. *Biochimica et biophysica acta* **835**: 253-257.

146. Murayama, N., Y. Asano, K. Kato, Y. Sakamoto, S. Hosoda, N. Yamada, T. Kodama, T. Murase, and Y. Akanuma. (1984) Effects of plasma infusion on plasma lipids, apoproteins and plasma enzyme activities in familial lecithin: cholesterol acyltransferase deficiency. *Eur J Clin Invest* **14**: 122-129.

147. Murayama, N., Y. Asano, S. Hosoda, M. Maesawa, M. Saito, F. Takaku, T. Sugihara, K. Miyashima, and Y. Yawata. (1984) Decreased sodium influx and abnormal red cell membrane lipids in a patient with familial plasma lecithin: cholesterol acyltransferase deficiency. *American journal of hematology* **16**: 129-137.

148. Albers, J. J., E. Gjone, J. L. Adolphson, C. H. Chen, P. Teisberg, and H. Torsvik. (1981) Familial lecithin-cholesterol acyltransferase deficiency in four Norwegian Families. Evidence for low levels of a functionally defective enzyme. *Acta Med Scand* **210**: 455-459.

149. Sakuma, M., Y. Akanuma, T. Kodama, N. Yamada, S. Murata, T. Murase, H. Itakura, and K. Kosaka. (1982) Familial plasma lecithin: cholesterol acyltransferase deficiency. A new family with partial LCAT activity. *Acta Med Scand* **212**: 225-232.

150. Albers, J. J., C. H. Chen, J. Adolphson, M. Sakuma, T. Kodama, and Y. Akanuma. (1982) Familial lecithin-cholesterol acyltransferase deficiency in a Japanese family: evidence for functionally defective enzyme in homozygotes and obligate heterozygotes. *Hum Genet* **62**: 82-85.

151. Clerc, M., M. F. Dumon, D. Sess, M. Freneix-Clerc, M. Mackness, and C. Conri. (1991) A 'Fish-eye disease' familial condition with massive corneal opacities and hypoalphalipoproteinaemia: clinical, biochemical and genetic features. *Eur J Clin Invest* **21**: 616-624.

152. Utermann, G., H. J. Menzel, P. Dieker, K. H. Langer, and G. Fiorelli. (1981) Lecithin-cholesterol-acyltransferase deficiency: autosomal recessive transmission in a large kindred. *Clinical genetics* **19**: 448-455.

153. Albers, J. J., and G. Utermann. (1981) Genetic control of lecithin-cholesterol acyltransferase (LCAT): measurement of LCAT mass in a large kindred with LCAT deficiency. *Am J Hum Genet* **33**: 702-708.

154. Naito, M., E. Maeda, G. Yoshino, M. Kasuga, A. Iguchi, and F. Kuzuya. (1994) Japanese family with a deficiency of lecithin:cholesterol acyltransferase (LCAT). *Internal medicine (Tokyo, Japan)* **33**: 677-682.

155. Dimick, S. M., B. Sallee, B. F. Asztalos, P. H. Pritchard, J. Frohlich, and E. J. Schaefer. (2014) A kindred with fish eye disease, corneal opacities, marked high-density lipoprotein deficiency, and statin therapy. *J Clin Lipidol* **8**: 223-230.

156. Savel, J., M. Lafitte, Y. Pucheu, V. Pradeau, A. Tabarin, and T. Couffinhal. (2012) Very low levels of HDL cholesterol and atherosclerosis, a variable relationship--a review of LCAT deficiency. *Vasc Health Risk Manag* **8**: 357-361.

157. Hanna, E. V., S. Simonelli, S. Chamney, A. Ossoli, and R. N. Mullan. (2018) Paradoxical fall in proteinuria during pregnancy in an LCAT-deficient patient-A case report. *J Clin Lipidol* **12**: 1151-1156.

158. Li, M., J. A. Kuivenhoven, A. F. Ayyobi, and P. H. Pritchard. (1998) T-->G or T-->A mutation introduced in the branchpoint consensus sequence of intron 4 of lecithin:cholesterol acyltransferase (LCAT) gene: intron retention causing LCAT deficiency. *Biochimica et biophysica acta* **1391**: 256-264.

159. Li, M., and P. H. Pritchard. (2000) Characterization of the effects of mutations in the putative branchpoint sequence of intron 4 on the splicing within the human lecithin:cholesterol acyltransferase gene. *The Journal of biological chemistry* **275**: 18079-18084.

160. Frohlich, J., R. McLeod, P. H. Pritchard, J. Fesmire, and W. McConathy. (1988) Plasma lipoprotein abnormalities in heterozygotes for familial lecithin:cholesterol acyltransferase deficiency. *Metabolism: clinical and experimental* **37**: 3-8.

161. Frohlich, J., G. Hoag, R. McLeod, M. Hayden, D. V. Godin, L. D. Wadsworth, J. D. Critchley, and P. H. Pritchard. (1987) Hypoalphalipoproteinemia resembling fish eye disease. *Acta Med Scand* **221**: 291-298.

162. National Center on Birth Defects and Developmental Disabilities, Centers for Disease Control and Prevention.

163. Eunice Kennedy Shriver National Institute of Child Health and Human Development (NICHD), National Institutes of Health (NIH).

164. Inker, L. A., C. H. Schmid, H. Tighiouart, J. H. Eckfeldt, H. I. Feldman, T. Greene, J. W. Kusek, J. Manzi, F. Van Lente, Y. L. Zhang, J. Coresh, and A. S. Levey. (2012) Estimating glomerular filtration rate from serum creatinine and cystatin C. *The New England journal of medicine* **367**: 20-29.

165. Levey, A. S., L. A. Stevens, C. H. Schmid, Y. L. Zhang, A. F. Castro, 3rd, H. I. Feldman, J. W. Kusek, P. Eggers, F. Van Lente, T. Greene, J. Coresh, and E. P. I. Ckd. (2009) A new equation to estimate glomerular filtration rate. *Ann Intern Med* **150**: 604-612.
